# Orthogonal Stability and Reactivity of Aryl Germanes Enables Rapid and Selective (Multi)Halogenations

**DOI:** 10.1002/anie.202008372

**Published:** 2020-08-20

**Authors:** Christoph Fricke, Kristina Deckers, Franziska Schoenebeck

**Affiliations:** ^1^ Institute of Organic Chemistry RWTH Aachen University Landoltweg 1 52074 Aachen Germany

**Keywords:** chemoselectivity, germanium, halogenation, reaction mechanisms, synthetic methods

## Abstract

While halogenation is of key importance in synthesis and radioimaging, the currently available repertoire is largely designed to introduce a single halogen per molecule. This report makes the selective introduction of several different halogens accessible. Showcased here is the privileged stability of nontoxic aryl germanes under harsh fluorination conditions (that allow selective fluorination in their presence), while displaying superior reactivity and functional‐group tolerance in electrophilic iodinations and brominations, outcompeting silanes or boronic esters under rapid and additive‐free conditions. Mechanistic experiments and computational studies suggest a concerted electrophilic aromatic substitution as the underlying mechanism.

## Graphical abstract

Nontoxic aryl germanes are shown to be of privileged stability under harsh fluorination conditions. They display superior reactivity and functional‐group tolerance in electrophilic iodinations and brominations, outcompeting silanes or boronic esters under rapid and additive‐free conditions.

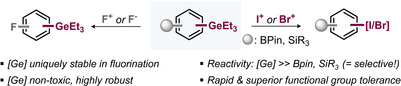

## Introduction

While aryl halides are of utmost importance as key functionalities to enable selective metal‐catalyzed C−C or C–heteroatom bond formations,^1^ they are also of importance beyond synthesis, impacting the activities of drugs,^2^, ^3^ material properties (e.g. solubility of nanoribbons)^4^ and (supramolecular) self‐assembly by halogen bonding.^5^ Moreover, the use of radioactive isotopes, especially ^18^F and ^123^I, allows for in vivo radioimaging via PET and SPECT techniques in the study of biological and physiological processes.^6^ Consequently, there is a significant interest in devising new halogenation strategies that satisfy the needs for efficiency, selectivity, nontoxicity, functional‐group tolerance as well as rapid speed.^7^ Impressive synthetic advances have been made in recent years, involving approaches of direct C−H functionalization via metal‐catalyzed or metal‐free (photoredox) halogenation strategies,^8^ halogen exchange (e.g. ArX to ArF)^9^ or the halogenation of suitable precursor functionalities, that is, boronic acid derivatives,^10^ silanes^11^ and stannanes.^12^

**Figure 1:**
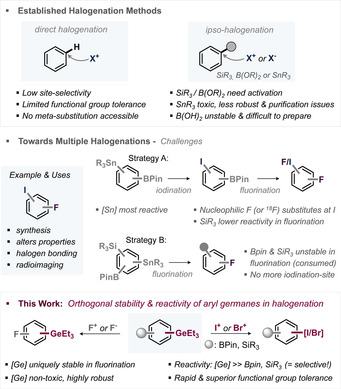
Challenges in halogenation approaches and this work.

However, the currently available synthetic repertoire was primarily developed for the introduction of a single halogen per precursor molecule. By contrast, the development of halogenated materials or drugs would greatly benefit form the ability to introduce multiple halogens late in a synthesis, since especially iodinated building blocks suffer from incompatibility of the C−I bonds with most metal‐catalyzed coupling chemistry, which in turn is powerful to connect building blocks to larger molecules.^13^ Moreover, in a radio‐halogenation context, the presence of more than one halogen in a molecule, especially I and F, could enable applications as a multifunctional radiotracer for SPECT and PET imaging via the introduction of the respective isotope [^123^I] or [^18^F] (Figure 1).^14^

Unselective halogenations cause challenges in separation of mixtures. In this context, both halogens are ideally introduced rapidly and with positional selectivity, as the size and electronic effects of the halogens impact the fate and binding efficiency^2^, ^3^ of a halogenated drug. Consequently, ideally, two separate and chemically orthogonal handles are employed, which allow for fully independent halogenations, irrespective of their relative positioning or the presence of additional functionality.

However, of the currently available “handles”, that is, B(OH)_2_, boronic esters, SiMe_3_ and SnBu_3_, the boronic acids are difficult to install, relatively unstable and challenging to purify.^15^ Stannanes show high reactivities in halogenation and find usage in commercial radiotracers;6d however, they are also toxic and purification of the toxic by‐products is frequently challenging. While aryl boronic esters or aryl silanes display comparatively greater stability,^16^ this comes at the expense of their reactivities in *ipso*‐iodination and bromination, which often require nucleophilic activation or metal catalysis to facilitate the reaction.9b, 10d, 10e, 11a, 11b, 17 This in turn negatively impacts scope and functional group tolerance.

**Figure 2:**
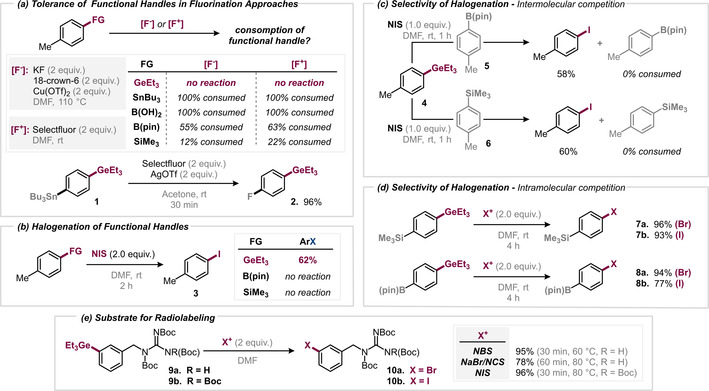
Organogermanes are uniquely stable in fluorination (a) and rapidly reactive in selective iodination/bromination reactions (b–e).

However, in the context of fluorination, all these functional groups are reactive (i.e. mostly unproductively consumed due to their instability), especially in the established radio‐fluorination methodologies based on KF/cryptand[2.2.2]/Cu(OTf)_2_ 18 or Selectfluor (see Figure 2 a). An initial fluorination, followed by iodination is therefore not accessible. Conversely, if there is initial iodination at the SnR_3_‐site, then Bpin or SiMe_3_ are not suitable to achieve efficient fluorination thereafter: while for SiMe_3_, fluorination is inefficient,^20^ the ^18^F installation at BPin is achieved via nucleophilic strategies (e.g. Cu(OTf)_2_(py)_4_ with KF at >100 °C), which lead to competing C−I substitution and overall product mixtures (see Figure 1).

As such, there is a need for a new and orthogonal functional group, and we targeted the trialkyl germanium functionality. This nontoxic^21^ class of reagents offers high stability against moisture, air, acids and bases,22b and has shown promise in reactions with molecular halogens.^19^ We recently uncovered that aryl germanes displayed privileged and orthogonal reactivities in metal catalysis over alternative functionalities,^22^, ^23^ and therefore envisioned that potentially orthogonal halogenations might also be feasible, which might unleash access to selective multi‐halogenation of molecules.

### Results and Discussion

We initially subjected 4‐tolyl germane to the established nucleophilic or electrophilic fluorination methods using KF, cryptand[2.2.2] and Cu(OTf)_2_ or Selectfluor (see Figure 2 a). Interestingly, the aryl germane fully tolerated these conditions: we recovered 4‐tolyl triethyl germane in >99 % yield. In stark contrast, the corresponding boron, silane and stannane compounds were fully consumed under these conditions (largely unproductively). There is hence a remarkable stability associated with germanes with respect to harsh fluorination conditions, which uniquely allow for fluorination in their presence. To investigate this further, we prepared the Sn/Ge‐containing bifunctional substrate **1** (Figure 2 a), and subjected it to fluorination. Remarkably, the application of the Selectfluor/AgOTf‐mediated fluorination^24^ protocol resulted in the fully selective fluorination of the SnBu_3_‐site in the presence of the GeEt_3_‐site. There was no consumption of the Ge‐functionality.

Conversely, the C–GeEt_3_ proved to be highly reactive in electrophilic iodination: the iodination of 4‐tolyl germane in DMF with *N*‐iodosuccinimide (NIS) occurred in good yield within 2 h at room temperature to give **3** (62 %), without the need for additives or metal catalysts (Figure 2 b).^25^ The corresponding bromination of 4‐methoxyphenyl germane proved to be even more facile and was complete within 15 min at room temperature (see SI for details). Consequently, this now offers the opportunity to introduce fluorine and subsequently iodine (or bromine) fully selectively.

We next explored our protocol in the halogenation towards *meta*‐iodobenzyl guanidine (MIBG) derivatives, which are used (as their stannane analogues) as tumor therapeutics or for diagnostic imaging via SPECT tomography (see Figure 2 e).^26^ To our delight, the iodination with NIS, and bromination with NBS proceeded in high yields and short reaction times. We also performed a halogenation with a mixture of *N*‐chlorosuccinimide (NCS) and NaBr (as radiolabeling generally relies on nucleophilic isotopes). Under these conditions, we successfully obtained [Br]MIBG **10 a** in 78 % yield after 60 min at 80 °C.

We next investigated the potential chemoselectivity of aryl germanes relative to boronic esters and silanes. Interestingly, despite its privileged robustness towards harsh fluorination conditions, the germane is much more reactive in iodination and bromination than the corresponding boronic ester derivatives or silanes, which are unreactive when employing similarly mild reaction conditions (see Figure 2 c/d). Our intermolecular competitions of halogenating aryl germanes versus traditional reagents showcased high chemoselectivity for germane functionalization over boronic acids, boronic esters and silanes (see Figure 2 c). Even substrates containing competing boronic ester (Bpin) or silane (SiMe_3_) functionalities showed exclusive and high reactivity at the C–GeEt_3_ bond, leaving the Bpin or SiMe_3_ moieties untouched (see Figure 2 d; **7 a**/**b** and **8 a**/**b**).

As such, the germanes show privileged robustness in fluorination, but superior reactivity in iodination and bromination, which allows for the selective introductions of the halogen couples I/Br, or F/I or F/Br.

To facilitate wider applications, knowledge about functional‐group tolerance in the respective iodination and halogenation of the germanium functionality is imperative. We therefore next assessed the scope of halogenation of organogermanes.

To our delight, a broad range of electron‐rich aryl germanes was halogenated in good to excellent yields (**3**, **11**–**15**, see Table 1). Especially trimethoxy‐phenyl‐ (**14**) or naphtyl‐derivatives (**15**) are often challenging to halogenate selectively, as they are prone to undergo multiple—and often unselective—competing direct C−H‐halogenation reactions.

**Table 1:** Scope of the halogenation.

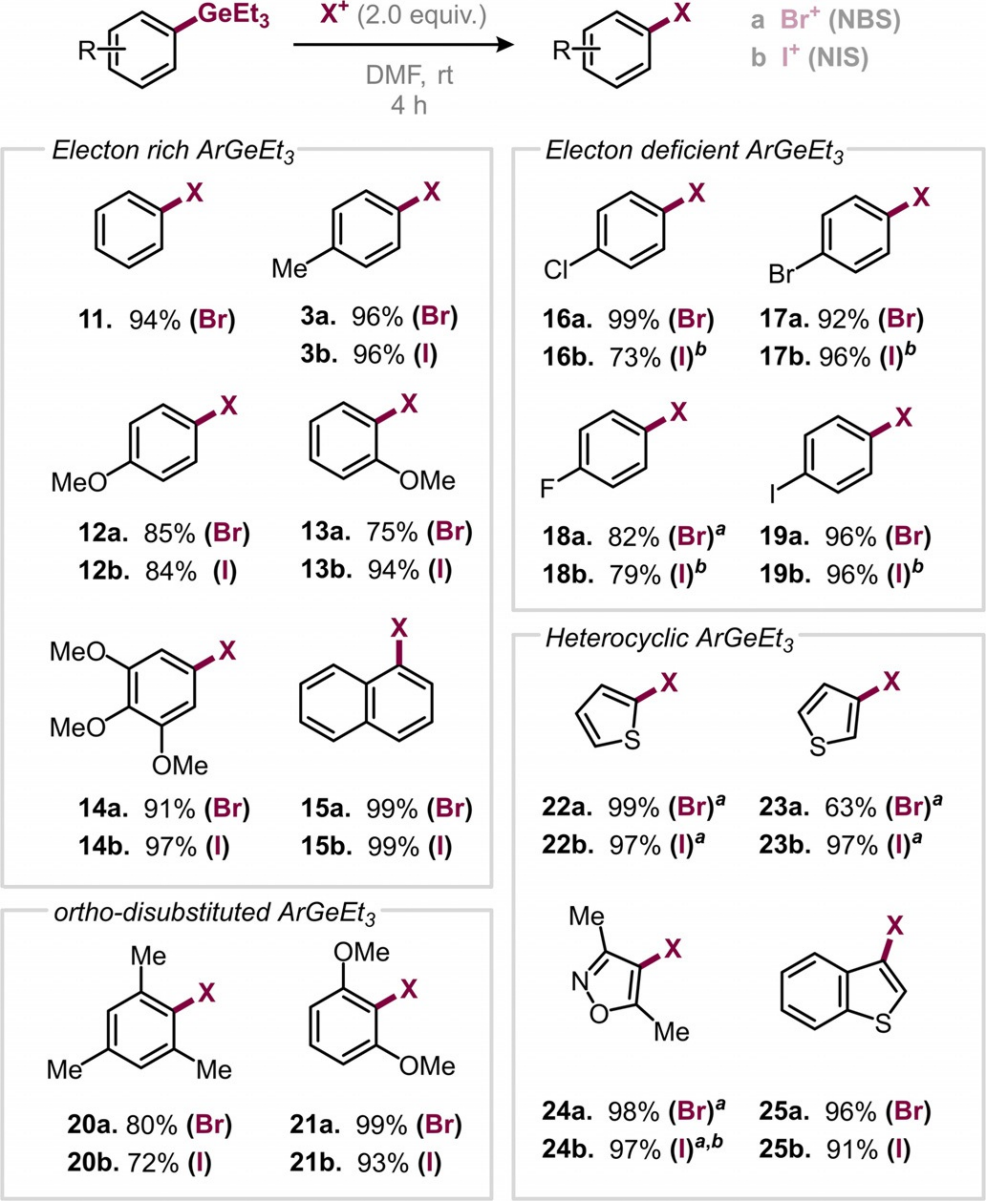

Yields of isolated products are given. [a] Yields determined by quantitative ^1^H NMR spectroscopy using mesitylene as internal standard. [b] Performed at 50 °C and with prolonged reaction time (see SI for details).

Pleasingly, also electron deficient aryl germanes, such as pre‐halogenated substrates were halogenated in excellent yields (**16**–**19**). This feature allows for the construction of multiply halogenated scaffolds for diverse synthetic purposes. Even sterically demanding *o*,*o*‐disubstituted (**20** and **21**) and heterocyclic scaffolds (**22**–**25**), whose boronic acid derivatives show a high tendency to decompose,^15^ underwent halogenation in high yields. Especially pleasing is the selective halogenation of 3‐thiophenyl substrates (**23** and **25**), as the 2‐thiophenyl‐position is the more nucleophilic site and hence usually preferentially targeted in reactions with electrophiles.

**Figure 3:**
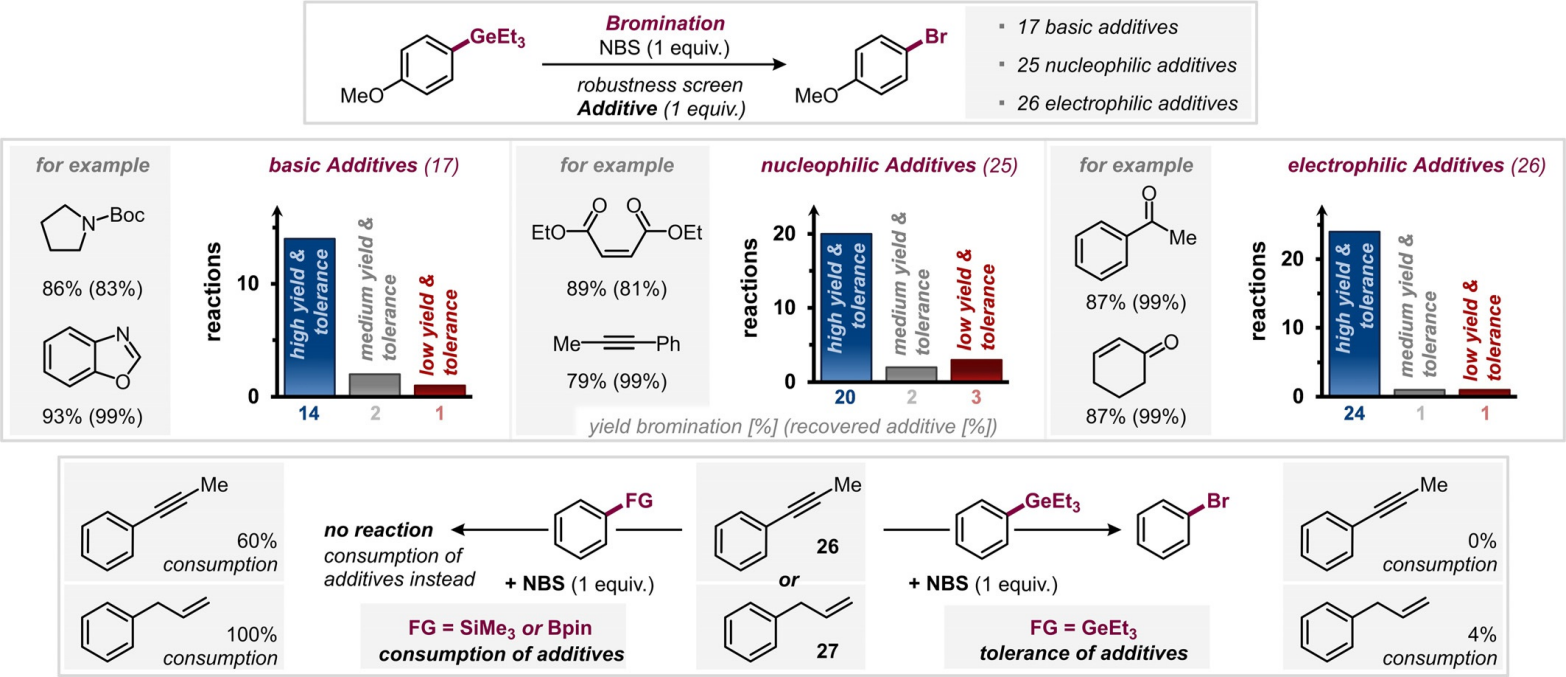
Additive screen to test functional‐group tolerance. Number of reactions with high yield (>66 %), medium yield (34–66 %) and low yield (<34 %).

Since electrophilic halogenation strategies can suffer from drawbacks such as strong oxidizing behavior or high affinity to react with for example, alkenes, alkynes^27^ or α‐acidic ketones^28^ in competing pathways,^30^ we further investigated the generality of our method with an additive screen.^31^ We tested a variety of potentially sensitive additives (Figure 3, top; see SI for details) and pleasingly found that owing to the privileged reactivity of the Ge‐functionality, numerous basic, nucleophilic and electrophilic additives were fully tolerated in the halogenation.

We tested 52 additives in total—including 16 heterocyclic, 24 carbonyl‐containing, and 11 N‐ or O‐protected compounds; 44 thereof were not affected by the halogenation and recovered after the reaction (in >66 %).

Most heterocycles, amides, lactones, and even activated alkenes, alkynes, or α‐acidic ketones as additives gave high yields of the desired halogenation on the germanium site, leaving the additive untouched. However, additives containing silyl‐protected alcohols or acidic protons (e.g. R‐OTMS or R‐NH_2_) underwent side reactions with the electrophilic halogenation reagent thus substantially lowering the yields.

In stark contrast, when we performed an analogous additive compatibility test with PhSiMe_3_ and PhBpin (see Figure 3, bottom), we found that while PhGeEt_3_ fully tolerated alkyne **26** and alkene **27** as additives in bromination with NBS, these functionalities were consumed in the corresponding reactions with PhBpin and PhSiMe_3_, and no bromination of BPin or SiMe_3_ took place.

**Figure 4:**
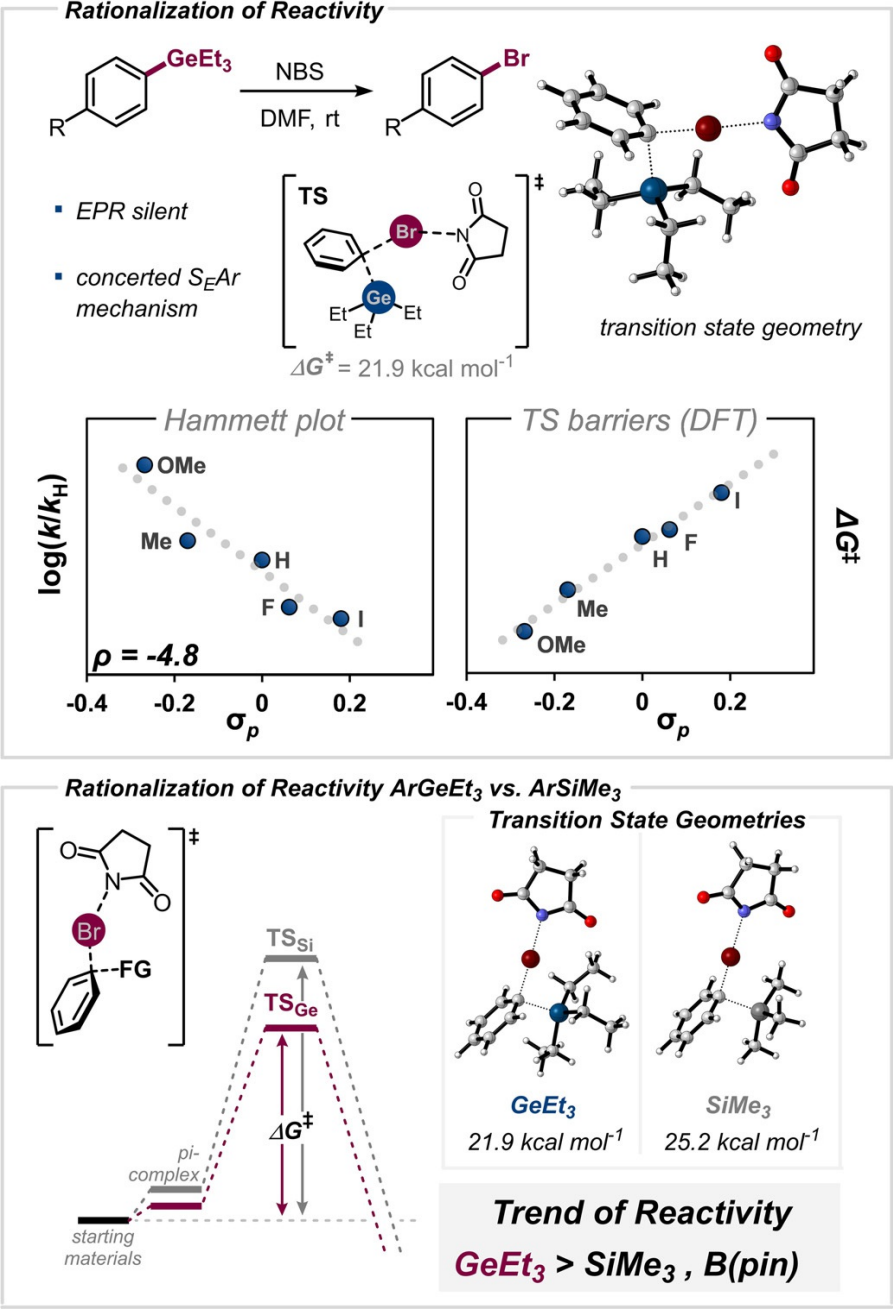
a) Experimental LFER analysis and computational study^29^ of the bromination using NBS. b) Comparison of transition‐state energies for aryl germanes and aryl silanes. Free energies in (a) and (b) computed at the CPCM (DMF) M06/6–311++G(d,p) (SDD)//ωB97XD/def2SVP level of theory. To account for charged intermediates, geometry optimizations were performed with an implicit solvent model. Free energies are given in kcal mol^−1^.

To gain insight on the origins of high reactivity of Ge in halogenation we next performed mechanistic investigations, combining a set of experimental and computational investigations (Figure 4). A linear free energy relationship (LFER) analysis of the reaction with a *ρ*_*σ*_ value of −4.8 indicated the build‐up of a positive charge in the transition state and hence supported the hypothesized pathway via S_*E*_Ar activation of the C−Ge bond.

In line with this, our computational studies indicated that germanium is halogenated in a *concerted* electrophilic aromatic substitution (in the gas‐phase and under implicit solvation optimization using CPCM solvation model). The generally assumed Wheland intermediate was not observed.^32^ An activation free energy barrier of Δ*G* ^≠^=21.9 kcal mol^−1^ was calculated^29^ for PhGeEt_3_, which is in line with the high experimental reactivity observed for ArGeEt_3_ at room temperature. For comparison, the corresponding aryl silane is predicted to react with a barrier of *ΔG* ^≠^=25.2 kcal mol^−1^, in line with the exclusive selectivity for Ge‐functionalization.

We further determined the activation barriers for other substituted germanes and plotted these against the corresponding σ‐parameter. A linear correlation between the electronic σ‐parameter^33^ of the *para*‐substituent and the corresponding activation barrier for the concerted transition state was observed.

In conclusion, we developed an operationally simple, rapid and widely applicable halogenation method for the selective introduction of iodine and bromine via concerted electrophilic aromatic substitution at germanium. While the aryl germane is superior in reactivity over silanes or boronic esters, it displays unique robustness towards fluorination conditions, which unleashes the possibility for chemoselective and orthogonal introduction of multiple different halogens (i.e. F/I, I/Br or F/Br) with complete positional control and wide functional‐group tolerance.

## Conflict of interest

The authors declare no conflict of interest.

## Supporting information

As a service to our authors and readers, this journal provides supporting information supplied by the authors. Such materials are peer reviewed and may be re‐organized for online delivery, but are not copy‐edited or typeset. Technical support issues arising from supporting information (other than missing files) should be addressed to the authors.

Supplementary
