# Supplementary material for: Orthogonal Stability and Reactivity of Aryl Germanes Enables Rapid and Selective (Multi)Halogenations
Source: Angew Chem Int Ed Engl. 2020 Aug 20;59(42):18717–22. doi: 10.1002/anie.202008372 (PMC7590071; doi:10.1002/anie.202008372)
Supplement: Supplementary file 1 — Supplementary [file ANIE-59-18717-s001.pdf]

Angewandte Chemie
Eine Zeitschrift der Gesellschaft Deutscher Chemiker

Supporting Information

Orthogonal Stability and Reactivity of Aryl Germanes Enables Rapid and Selective (Multi)Halogenations

*Christoph Fricke, Kristina Deckers, and Franziska Schoenebeck**

anie_202008372_sm_miscellaneous_information.pdf

Table of Contents

General Information 3

Bromination 5

Iodination 10

Synthesis of Aryl Germanes 15

Halolabeling of Physiologically Relevant Target Molecule 24

Tolerance and Orthogonality of Halogenation Approaches 27

Tolerance of Aryl Iodides in Fluorination Approaches 27

Compatibility of Functional Handles in Halogenation Approaches 27

Tolerance of Functional Handles in Fluorination Approaches 28

Selective Fluorination of $SnBu_3$ vs. $GeEt_3$ 29

Intermolecular Competition in Iodination 29

Individual Bromination and Iodination 30

Intramolecular Competition 31

Mechanistic Investigation 33

Linear Free Energy Relationship Analysis (Hammett Plot) 33

Robustness Screen 35

Robustness Screen for ArB(pin) and $ArSiMe_3$ 38

EPR Analysis 39

Reaction Monitoring 41

Computational Details 42

Influence of para-Substituents on the Transition State Barrier (DFT) 43

Comparison of Aryl Germanes with Aryl Silanes 44

XYZ Coordinates and Energies 45

NMR Spectra 57

References 82

General Information

Reagents and Solvents

Unless otherwise stated, all reagents and starting materials were commercially available and used as received. Solvents used in work up and purification were distilled prior to use. Anhydrous and degassed dimethylformamide (DMF) and acetone were purchased from Sigma Aldrich. Dry and anhydrous THF was obtained from an SP-105 solvent drying system from LC Technology Solutions Inc..

Experimental Techniques

All reactions involving air- or moisture-sensitive reagents or intermediates were carried out in dried glassware under an argon atmosphere and were performed either in an argon-filled glovebox or by using standard *Schlenk* techniques unless otherwise stated.
Column chromatography was carried out using silica gel (35–70 mesh; 60 Å). Thin layer chromatography (TLC) was performed on Merck silica gel 60 F254 aluminum plates; detection either under UV light or by dipping into a solution of $KMnO_4$ (1.5 g) and $NaHCO_3$ (5.0 g) in H_2O (400 mL) followed by heating. Preparative HPLC was performed on a Gilson-Abimed HPLC (employing UV detector model 117) using a Merck LiChrosorb Si60 column (porosity 7 μm, 250 x 25 mm).
All ^{1}H, ^{13}C and ^{19}F NMR spectra were recorded at ambient temperature on a Varian V NMRS 600 or a Varian V NMRS 400 spectrometer. Chemical shifts (δ) are reported in parts per million (ppm) relative to $SiMe_4$ and referenced to either the residual solvent peak for ^{1}H (7.26 ppm for $CDCl_3$ and 2.92 ppm for DMF-d_7) and ^{13}C NMR spectra (77.2 ppm for $CDCl_3$ and 163.2 ppm for DMF-d_7) or internally by the instrument after locking and shimming to the deuterated solvent (for ^{19}F). Coupling constants (*J*) are given in Hertz (Hz).
High-resolution mass spectrometry (HRMS) was performed using a Thermo Scientific LTQ Orbitrap XL (ESI) or an Finnigan MAT 95 (EI, 70 eV). IR spectra were recorded on a Perkin Elmer Spectrum 100 spectrometer with an UATR Diamond/KRS-5 crystal with attenuated total reflectance (ATR). Relative intensities are given in parentheses (w = weak, m = medium, s = strong).
Low-resolution mass spectrometry and reaction monitoring were performed with an Agilent Technologies 5975 series MSD mass spectrometer under electron ionization (EI) mode coupled with an Agilent Technologies 7820A gas chromatograph employing an Agilent HP-5MS column (30 m × 0.25 mm inner diameter × 0.25 μm (5% phenyl)-methylpolysiloxane film) or an Agilent CP-Sil8-CB column (30 m × 0.25 mm inner diameter × 1.00 μm (5% phenyl)-

methylpolysiloxane film). Operating with a constant He-flow of 1.2 mL min^{-1}, injector temperature 250 °C, detector-line temperature 280 °C.

EPR analysis was performed with a Miniscope MS400 spectrometer from Freiberg Instruments with a FC 400 frequency generator.

Bromination

General Procedure 1 (GP 1)

Aryl germane (1.0 equiv.) and *N*-bromosuccinimide (NBS; 2.0 equiv.) were added to the reaction vial in air, dissolved in DMF (0.3 M) and stirred at room temperature for 4 h. After completion of reaction, the reaction was quenched by addition of aqueous solution of $Na_2S_2O_3$ (sat.), the organic phase was separated and the aqueous phase was extracted with DCM (3x). The combined organic phases were dried over $MgSO_4$, the solvent was removed under reduced pressure and the crude product mixture was purified by silica column chromatography. Some of the brominated compounds are not isolable due to their high volatility. In these cases, the yield was determined by ^{1}H NMR (quant., ethylene carbonate as internal standard) or ^{19}F NMR (quant., 1,4-difluorobiphenyl as internal standard) and the further analysis for unambiguous characterization was performed using the crude reaction mixture.

Bromobenzene

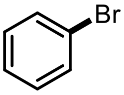

Prepared according to GP 1. The yield was determined by quantitative ^{1}H NMR (94%). **^{1}H NMR** (600 MHz, DMF-d_7) δ/ ppm = 7.64 – 7.59 (m, 2H), 7.45 – 7.41 (m, 1H), 7.41 – 7.36 (m, 2H). **^{13}C NMR** (151 MHz, DMF-d_7) δ/ ppm = 132.5, 131.7, 128.5, 123.1. **MS** (70 eV, EI): *m/z* (%): 158.0 (62) $[M]^+$ (^{81}Br), 156.0 (64) $[M]^+$ (^{79}Br), 77.0 (100), 74.0 (21), 51.0 (42).
Due to its high volatility, the title compound was not isolated.

1-Bromo-4-methylbenzene

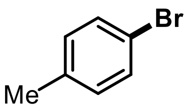

Prepared according to GP 1. The yield was determined by quantitative ^{1}H NMR (96%).
^{1}H NMR (600 MHz, DMF-d_7) δ/ ppm = 7.47 (d, *J* = 8.3 Hz, 2H), 7.20 (d, *J* = 8.3 Hz, 2H), 2.30 (s, 3H). **^{13}C NMR** (151 MHz, DMF-d_7) δ/ ppm = 138.3, 132.3, 132.2, 119.6, 21.2. **MS** (70 eV, EI): *m/z* (%): 172.0 (36) $[M]^+$ (^{81}Br), 170.0 (63) $[M]^+$ (^{79}Br), 91.1 (100), 89.1 (18), 65.0 (20), 63.0 (20).
Due to its high volatility, the title compound was not isolated.

5-Bromo-1,2,3-trimethoxybenzene

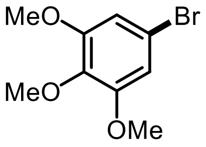

Prepared according to GP 1. The title product was obtained after purification by column chromatography (*n*-pentane/Et_2O, 5:1) as a white solid (68.4 mg, 0.277 mmol, 91%).

R_f = 0.51 (*n*-pentane/Et_2O, 5:1). **^{1}H NMR** (600 MHz, $CDCl_3$) δ/ ppm = 6.71 (s, 2H), 3.84 (s, 6H), 3.81 (s, 3H). **^{13}C NMR** (151 MHz, $CDCl_3$) δ/ ppm = 154.0, 137.4, 116.3, 109.0, 61.0, 56.4. **HRMS** (EI) calculated for $C_9H_{11}O_3{}^{79}Br$: 268.9784 $[M+Na]^+$, found: 268.9784.
These data are in agreement with those reported previously in the literature.[1]

1-Bromonaphthalene

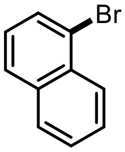

Prepared according to GP 1. The title product was obtained after purification by column chromatography (*n*-pentane) as a colorless oil (62.0 mg, 0.300 mmol, 99%). **R_f** = 0.80 (*n*-pentane). **^{1}H NMR** (600 MHz, $CDCl_3$) δ/ ppm = 8.25 (d, *J* = 8.5 Hz, 1H), 7.86 – 7.78 (m, 3H), 7.61 (dd, *J* = 8.5, 6.8 Hz, 1H), 7.54 (dd, *J* = 8.0, 6.8 Hz, 1H), 7.33 (dd, *J* = 8.0, 7.4 Hz, 1H). **^{13}C NMR** (151 MHz, $CDCl_3$) δ/ ppm = 134.7, 132.1, 130.0, 128.4, 128.1, 127.5, 127.2, 126.8, 126.3, 123.0. **HRMS** (EI) calculated for $C_{10}H_7{}^{79}Br$: 205.9726 $[M]^+$, found: 205.9728.
These data are in agreement with those reported previously in the literature.[2]

1-Bromo-4-methoxybenzene

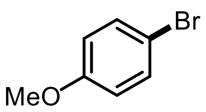

Prepared according to GP 1. The title product was obtained after purification by column chromatography (*n*-pentane) as a colorless oil (48.4 mg, 0.259 mmol, 85%).

R_f = 0.90 (*n*-pentane). **^{1}H NMR** (600 MHz, $CDCl_3$) δ/ ppm = 7.38 (d, *J* = 8.9 Hz, 2H), 6.78 (d, *J* = 8.9 Hz, 2H), 3.78 (d, *J* = 1.0 Hz, 3H). **^{13}C NMR** (151 MHz, $CDCl_3$) δ/ ppm = 158.8, 132.4, 115.8, 112.9, 55.6. **HRMS** (EI) calculated for $C_7H_7O^{79}Br$: 185.9675 $[M]^+$, found: 185.9670.
These data are in agreement with those reported previously in the literature.[3]

1-Bromo-2-methoxybenzene

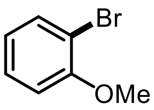

Prepared according to GP 1. The title product was obtained after purification by column chromatography (*n*-pentane) as a colorless oil (42.4 mg, 0.227 mmol, 75%).

R_f = 0.68 (*n*-pentane). **^{1}H NMR** (600 MHz, $CDCl_3$) δ/ ppm = 7.54 (dd, *J* = 7.9, 1.6 Hz, 1H), 7.32 – 7.25 (m, 1H), 6.91 (d, *J* = 8.2 Hz, 1H), 6.87 – 6.80 (m, 1H), 3.90 (s, 3H). **^{13}C NMR** (151 MHz, $CDCl_3$)

δ/ ppm = 156.0, 133.5, 128.6, 121.9, 112.1, 111.8, 56.3. **HRMS** (EI) calculated for $C_7H_7O^{79}Br$: 185.9675 [M]$^+$, found: 185.9674.

These data are in agreement with those reported previously in the literature.[4]

2-Bromo-1,3,5-trimethylbenzene

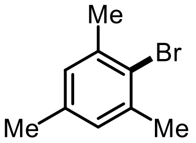

Prepared according to GP 1. The title product was obtained after purification by column chromatography (*n*-pentane) as a colorless oil (48.4 mg, 0.243 mmol, 80%).

R$_f$ = 0.90 (*n*-pentane). **^{1}H NMR** (600 MHz, $CDCl_3$) δ/ ppm = 6.90 (s, 2H), 2.38 (s, 6H), 2.24 (s, 3H). **^{13}C NMR** (151 MHz, $CDCl_3$) δ/ ppm = 138.0, 136.4, 129.2, 124.3, 23.9, 20.8. **HRMS** (EI) calculated for $C_9H_{11}{}^{79}Br$: 198.0039 [M]$^+$, found: 198.0034.

These data are in agreement with those reported previously in the literature.[3]

2-Bromo-1,3-dimethoxybenzene

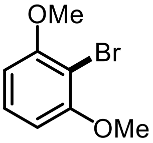

Prepared according to GP 1. The title product was obtained after purification by column chromatography (pentane/Et_2O, 5:1) as a white solid (67.0 mg, 0.309 mmol, 99%).

R$_f$ = 0.65 (pentane/Et_2O, 8:1). **^{1}H NMR** (600 MHz, $CDCl_3$) δ/ ppm = 7.23 (t, *J* = 8.3 Hz, 1H), 6.58 (d, *J* = 8.3 Hz, 2H), 3.90 (s, 6H). **^{13}C NMR** (151 MHz, $CDCl_3$) δ/ ppm = 157.3, 128.4, 104.8, 101.0, 56.6. **HRMS** (EI) calculated for $C_8H_9O_2{}^{79}Br$: 215.9780 [M]$^+$, found: 215.9782.

These data are in agreement with those reported previously in the literature.[5]

1-Bromo-4-iodobenzene

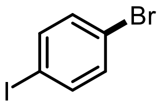

Prepared according to GP 1. The title product was obtained after purification by column chromatography (*n*-pentane) as a white solid (83.4 mg, 0.295 mmol, 96%). **R$_f$** = 0.90 (*n*-pentane). **^{1}H NMR** (600 MHz, $CDCl_3$) δ/ ppm = 7.54 (d, *J* = 8.5 Hz, 2H), 7.23 (d, *J* = 8.5 Hz, 2H). **^{13}C NMR** (151 MHz, $CDCl_3$) δ/ ppm = 139.2, 133.6, 122.3, 92.2. **HRMS** (EI) calculated for $C_6H_4{}^{79}BrI$: 281.8536 [M]$^+$, found: 281.8523.

These data are in agreement with those reported previously in the literature.[6]

1,4-Dibromobenzene

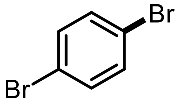

Prepared according to GP 1. The title product was obtained after purification by column chromatography (*n*-pentane) as a white solid (64.8 mg, 0.275 mmol, 92%).

R$_f$ = 0.90 (*n*-pentane). **^{1}H NMR** (600 MHz, $CDCl_3$) δ/ ppm = 7.36 (s, 4H). **^{13}C NMR** (151 MHz, $CDCl_3$) δ/ ppm = 133.3, 121.2. **HRMS** (EI) calculated for $C_6H_4{}^{79}Br_2$: 233.8674 $[M]^+$, found: 233.8673.

These data are in agreement with those reported previously in the literature.[7]

1-Bromo-4-chlorobenzene

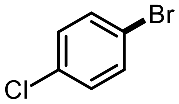

Prepared according to GP 1. The yield was determined by quantitative ^{1}H NMR (99%). The title product was obtained after purification by column chromatography (*n*-pentane) as a white solid.

R$_f$ = 0.87 (*n*-pentane). **^{1}H NMR** (600 MHz, $CDCl_3$) δ/ ppm = 7.42 (d, *J* = 8.7 Hz, 2H), 7.21 (d, *J* = 8.7 Hz, 2H). **^{13}C NMR** (151 MHz, $CDCl_3$) δ/ ppm = 133.4, 132.9, 130.3, 120.4. **HRMS** (EI) calculated for $C_6H_4{}^{79}Br^{35}Cl$: 289.9179 $[M]^+$, found: 289. 9181.

Due to its high volatility, the title compound was not isolated in a representative yield.

These data are in agreement with those reported previously in the literature.[7]

1-Bromo-4-fluorobenzene

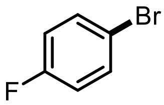

Prepared according to GP 1. The yield was determined by quantitative ^{1}H NMR (82%).

^{1}H NMR (600 MHz, DMF-d_7) δ/ ppm = 7.69 – 7.62 (m, 2H), 7.28 – 7.22 (m, 2H). **^{19}F NMR** (564 MHz, DMF-d_7) δ/ ppm = -116.06 – -116.34 (m). **MS** (70 eV, EI): *m/z* (%): 176.0 (90) $[M]^+$ (^{81}Br), 174.0 (91) $[M]^+$ (^{79}Br), 95.0 (100), 94 (11), 75 (46), 74 (15).

Due to its high volatility, the title compound was not isolated. The resolution of the NMR made it impossible to assign the correct ^{13}C NMR shifts.

4-Bromo-3,5-dimethylisoxazole

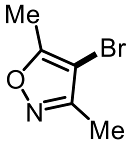

Prepared according to GP 1. The yield was determined by quantitative ^{1}H NMR (98%).

^{1}H NMR (600 MHz, DMF-d_7) δ/ ppm = 2.43 (s, 3H), 2.24 (s, 3H). **^{13}C NMR** (151 MHz, DMF-d_7) δ/ ppm = 167.9, 160.2, 93.2, 11.6, 10.9. **MS** (70 eV, EI): *m/z* (%): 177.0 (21) $[M]^+$ (^{81}Br),

175.0 (22) [M]$^+$ (^{79}Br), 161.9 (28), 133.9 (43), 132.9 (81), 131.9 (56), 118.9 (18), 107.9 (59), 90.0 (29), 81.0 (34), 79.9 (16), 68.1 (27), 64.0 (16), 62.0 (43), 55.0 (13), 54.0 (100), 52.0 (80), 51.0 (67).
Due to its high volatility, the title compound was not isolated.

3-Bromobenzo[b]thiophene

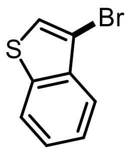

Prepared according to GP 1. The title product was obtained after purification by column chromatography (*n*-pentane) as a colorless oil (62.5 mg, 0.293 mmol, 96%). **R$_f$** = 0.82 (pentane). **^{1}H NMR** (600 MHz, $CDCl_3$) δ/ ppm = 7.85 (dd, *J* = 7.3, 7.3 Hz, 2H), 7.51 – 7.39 (m, 3H). **^{13}C NMR** (151 MHz, $CDCl_3$) δ/ ppm = 138.7, 137.6, 125.4, 125.1, 123.6, 123.2, 122.8, 107.8. **HRMS** (EI) calculated for $C_8H_5{}^{79}BrS$: 211.9290 [M]$^+$, found: 211.9282. These data are in agreement with those reported previously in the literature.[8]

2-Bromothiophene

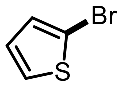

Prepared according to GP 1. The yield was determined by quantitative ^{1}H NMR (99%). **^{1}H NMR** (400 MHz, DMF-d$_7$) δ/ ppm = 7.66 – 7.59 (m, 1H), 7.29 – 7.22 (m, 1H), 7.06 – 6.99 (m, 1H). **^{13}C NMR** (151 MHz, DMF-d$_7$) δ/ ppm = 156.0, 130.7, 128.5, 111.4. **MS** (70 eV, EI): *m/z* (%): 163.9 (84) [M]$^+$ (^{81}Br), 161.9 (79) [M]$^+$ (^{79}Br), 117.0 (12), 83.0 (100), 82.0 (30), 81.0 (49), 78.9 (25), 58.0 (13), 57.0 (54).
Due to its high volatility, the title compound was not isolated.

3-Bromothiophene

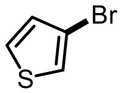

Prepared according to GP 1. The yield was determined by quantitative ^{1}H NMR (63%). **^{1}H NMR** (600 MHz, DMF-d$_7$) δ/ ppm = 7.81 (d, *J* = 5.7 Hz, 1H), 7.72 – 7.68 (m, 1H), 7.20 – 7.15 (m, 1H). **MS** (70 eV, EI): *m/z* (%):163.9 (100) [M]$^+$ (^{81}Br), 161.9 (94) [M]$^+$ (^{79}Br), 118.9 (12), 116.9 (12), 83.0 (90), 82.0 (40), 81.0 (61), 80.0 (11), 78.9 (27), 57.0 (30), 57.0 (54).
Due to its high volatility, the title compound was not isolated. The resolution of the NMR spectrum made it impossible to assign the correct ^{13}C NMR shifts.

Iodination

General Procedure 2 (GP 2)

Aryl germane (1.0 equiv.) and *N*-iodosuccinimide (NIS; 1.0 equiv.) were added to the reaction vial in air, dissolved in DMF (0.3 M) and stirred at room temperature or at 50 °C for 4 h. After completion of reaction (monitored by GC-MS or TLC), the reaction was quenched by addition of aqueous solution of $Na_2S_2O_3$ (sat.), the organic phase was separated and the aqueous phase was extracted with DCM (3x). The combined organic phases were dried with $MgSO_4$, the solvent was removed under reduced pressure and the crude product mixture was purified over silica column chromatography. Some of the iodinated compounds are not isolable due to their high volatility. An ^{1}H NMR (quant.) or ^{19}F NMR (quant.) yield is given in those cases and the further analysis was performed using the crude reaction mixture.

1-Iodo-4-methylbenzene

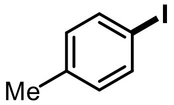

Prepared according to GP 2 at room temperature. The yield was determined by quantitative ^{1}H NMR (96%).

^{1}H NMR (600 MHz, DMF-d_7) δ/ ppm = 7.64 (d, *J* = 7.9 Hz, 2H), 7.06 (d, *J* = 7.9 Hz, 2H). **^{13}C NMR** (151 MHz, DMF-d_7) δ/ ppm = 156.9, 138.8, 138.3, 132.6, 66.3. **MS** (70 eV, EI): *m/z* (%): 218.0 (100) $[M]^+$, 126.9 (19), 91.1 (73), 89.1 (14), 65.1 (29), 63 (13).

Due to its high volatility, the title compound was not isolated.

5-Iodo-1,2,3-trimethoxybenzene

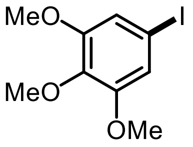

Prepared according to GP 2 at room temperature. The title product was obtained after purification by column chromatography (*n*-pentane/Et_2O, 5:1) as a white solid (85.4 mg, 0.290 mmol, 97%).

R_f = 0.40 (*n*-pentane/Et_2O, 7:1). **^{1}H NMR** (600 MHz, $CDCl_3$) δ/ ppm = 6.88 (d, *J* = 1.3 Hz, 2H), 3.83 (d, *J* = 1.5 Hz, 6H), 3.81 (d, *J* = 1.3 Hz, 3H). **^{13}C NMR** (151 MHz, $CDCl_3$) δ/ ppm = 154.1, 138.3, 115.1, 86.3, 61.0, 56.4. **HRMS** (EI) calculated for $C_9H_{11}O_3I$: 293.9748 $[M]^+$, found: 293.9749.

These data are in agreement with those reported previously in the literature.[9]

1-Iodonaphthalene

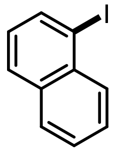

Prepared according to GP 2 at room temperature. The title product was obtained after purification by column chromatography (*n*-pentane) as a colorless oil (77.4 mg, 0.305 mmol, 99%).

R_f = 0.62 (*n*-pentane). **^{1}H NMR** (600 MHz, $CDCl_3$) δ/ ppm = 8.10 (ddd, *J* = 7.3, 7.3, 1.6 Hz, 2H), 7.84 (d, *J* = 8.1 Hz, 1H), 7.78 (d, *J* = 8.1 Hz, 1H), 7.59 (ddd, *J* = 8.4, 6.9, 1.3 Hz, 1H), 7.53 (ddd, *J* = 8.1, 6.9, 1.3 Hz, 1H), 7.22 – 7.14 (m, 1H). **^{13}C NMR** (151 MHz, $CDCl_3$) δ/ ppm = 137.6, 134.5, 134.3, 132.3, 129.1, 128.7, 127.9, 127.0, 126.9, 99.7. **HRMS** (EI) calculated for $C_{10}H_7I$: 253.9587 $[M]^+$, found: 253.9580.

These data are in agreement with those reported previously in the literature.[10]

1-Iodo-4-methoxybenzene

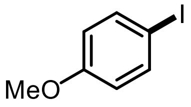

Prepared according to GP 2 at room temperature. The title product was obtained after purification by column chromatography (*n*-pentane) as a white solid (59.0 mg, 0.252 mmol, 84%).

R_f = 0.30 (*n*-pentane). **^{1}H NMR** (600 MHz, $CDCl_3$) δ/ppm = 7.56 (d, *J* = 9.0 Hz, 2H), 6.68 (d, *J* = 9.0 Hz, 2H), 3.78 (s, 3H). **^{13}C NMR** (151 MHz, $CDCl_3$) δ/ppm = 159.6, 138.3, 116.5, 82.8, 55.4. **HRMS** (EI) calculated for C_7H_7OI: 233.9536 $[M]^+$, found: 233.9544.

These data are in agreement with those reported previously in the literature.[6]

1-Iodo-2-methoxybenzene

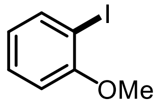

Prepared according to GP 2 at room temperature. The title product was obtained after purification by column chromatography (*n*-pentane) as a colorless oil (66.2 mg, 0.283 mmol, 94%).

R_f = 0.46 (*n*-pentane). **^{1}H NMR** (600 MHz, $CDCl_3$) δ/ ppm = 7.77 (dd, *J* = 7.8, 1.6 Hz, 1H), 7.31 (ddd, *J* = 8.7, 7.4, 1.6 Hz, 1H), 6.83 (dd, *J* = 8.1, 1.3 Hz, 1H), 6.71 (ddd, *J* = 7.4, 7.4, 1.3 Hz, 1H), 3.88 (s, 3H). **^{13}C NMR** (151 MHz, $CDCl_3$) δ/ ppm = 158.2, 139.6, 129.7, 122.6, 111.1, 86.1, 56.4. **HRMS** (EI) calculated for C_7H_7OI: 233.9536 $[M]^+$, found: 233.9530.

These data are in agreement with those reported previously in the literature.[3]

2-Iodo-1,3,5-trimethylbenzene

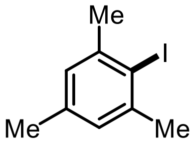

Prepared according to GP 2 at room temperature. The title product was obtained after purification by column chromatography (*n*-pentane) as a white solid (54.4 mg, 0.221 mmol, 72%).

R$_f$ = 0.77 (*n*-pentane). **^{1}H NMR** (600 MHz, $CDCl_3$) δ/ ppm = 6.89 (s, 2H), 2.43 (s, 6H), 2.24 (s, 3H). **^{13}C NMR** (151 MHz, $CDCl_3$) δ/ ppm = 141.9, 137.5, 128.1, 104.4, 29.6, 20.8. **HRMS** (EI) calculated for $C_9H_{11}I$: 245.9900 $[M]^+$, found: 245.9900.

These data are in agreement with those reported previously in the literature.[3]

2-Iodo-1,3-dimethoxybenzene

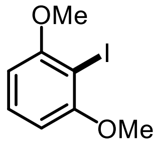

Prepared according to GP 2 at room temperature. The title product was obtained after purification by column chromatography (*n*-pentane/Et_2O, 7:1) as a yellow solid (74.7 mg, 0.283 mmol, 93%).

R$_f$ = 0.61 (*n*-pentane/Et_2O, 7:1). **^{1}H NMR** (600 MHz, $CDCl_3$) δ/ ppm = 7.27 (td, *J* = 8.1, 1.4 Hz, 1H), 6.52 (dd, *J* = 8.1, 1.4 Hz, 2H), 3.90 (d, *J* = 1.4 Hz, 6H). **^{13}C NMR** (151 MHz, $CDCl_3$) δ/ ppm = 159.7, 130.0, 104.2, 77.8, 56.7. **HRMS** (EI) calculated for $C_7H_6O_2I$: 248.9407 $[M-Me]^+$, found: 248.9403.

These data are in agreement with those reported previously in the literature.[5]

1,4-Diiodobenzene

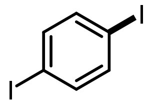

Prepared according to GP 2 at 50 °C; reaction time 24 h. The title product was obtained after purification by column chromatography (*n*-pentane) as a white solid (94.3 mg, 0.286 mmol, 96%).

R$_f$ = 0.76 (*n*-pentane). **^{1}H NMR** (600 MHz, $CDCl_3$) δ/ ppm = 7.41 (s, 4H). **^{13}C NMR** (151 MHz, $CDCl_3$) δ/ ppm = 139.5, 93.5. **HRMS** (EI) calculated for $C_6H_4I_2$: 329.8397 $[M]^+$, found: 329.8398.

These data are in agreement with those reported previously in the literature.[11]

1-Iodo-4-bromobenzene

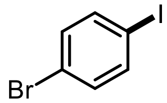

Prepared according to GP 2 at 50 °C; reaction time 24 h. The title product was obtained after purification by column chromatography (pentane) as a white solid (81.3 mg, 0.287 mmol, 96%).

R$_f$ = 0.90 (*n*-pentane). **^{1}H NMR** (600 MHz, $CDCl_3$) δ/ ppm = 7.54 (d, *J* = 8.5 Hz, 2H), 7.23 (d, *J* = 8.5 Hz, 2H). **^{13}C NMR** (151 MHz, $CDCl_3$) δ/ppm = 139.2, 133.6, 122.3, 92.2. **HRMS** (EI) calculated for $C_6H_4{}^{79}BrI$: 281.8536 $[M]^+$, found: 281.8522.

These data are in agreement with those reported previously in the literature.[11]

1-Iodo-4-chlorobenzene

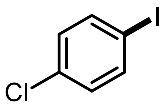

Prepared according to GP 2 at 50 °C; reaction time 24 h. The title product was obtained after purification by column chromatography (*n*-pentane) as a white solid (52.0 mg, 0.218 mmol, 73%).

R_f = 0.79 (pentane). **^{1}H NMR** (600 MHz, $CDCl_3$) δ/ ppm = 7.61 (d, *J* = 8.6 Hz, 2H), 7.09 (d, *J* = 8.6 Hz, 2H). **^{13}C NMR** (151 MHz, $CDCl_3$) δ/ppm = 138.9, 134.4, 130.7, 91.3. **HRMS** (EI) calculated for $C_6H_4{}^{35}ClI$: 237.9041 $[M]^+$, found: 237.9044.

These data are in agreement with those reported previously in the literature.[11]

1-Iodo-4-fluorobenzene

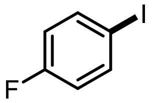

Prepared according to GP 2 at 50 °C; reaction time 24 h. The yield was determined by quantitative ^{1}H NMR (79%).

^{1}H NMR (600 MHz, DMF-d_7) δ/ ppm = 7.85 – 7.79 (m, 2H), 7.11 (dd, *J* = 8.3 Hz, 2H). **^{13}C NMR** (151 MHz, DMF-d_7) δ/ ppm = 163.02 (d), 139.60 (d, *J* = 8.0 Hz), 127.20, 118.19 (d, *J* = 22.2 Hz). **^{19}F NMR** (564 MHz, DMF-d_7) δ/ ppm = -115.6. **MS** (70 eV, EI): *m/z* (%): 221.9 (100), 126.9 (19), 95.0 (58), 75.0 (35), 74.0 (10).

Due to its high volatility, the title compound was not isolated.

4-Iodo-3,5-dimethylisoxazole

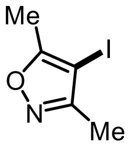

Prepared according to GP 2 at 50 °C; reaction time 24 h. The yield was determined by quantitative ^{1}H NMR (97%).

^{1}H NMR (600 MHz, DMF-d_7) δ/ppm = 2.45 (s, 3H), 2.22 (s, 3H). **MS** (70 eV, EI): *m/z* (%): 223.0 (100) $[M]^+$, 207.9 (20), 180.9 (31), 179.98 (14), 53.9 (11), 126.9 (37), 96.0 (25), 81.0 (14), 63.0 (12), 54.0 (44), 52.0 (15).

Due to its high volatility, the title compound was not isolated. The resolution of the NMR made it impossible to assign the correct ^{13}C NMR shifts.

3-Iodobenzo[b]thiophene

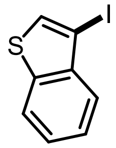

Prepared according to GP 2 at room temperature. The title product was obtained after purification by column chromatography (*n*-pentane) as a colorless oil (70.8 mg, 0.272 mmol, 91%).

$\mathbf{R_f}$ = 0.80 (*n*-pentane). **^{1}H NMR** (600 MHz, $CDCl_3$) δ/ ppm = 7.86 (d, *J* = 8.2 Hz, 1H), 7.77 (dd, *J* = 8.2 Hz, 1H), 7.62 (s, 1H), 7.48 (dd, *J* = 7.2 Hz, 1H), 7.40 (dd, *J* = 7.2 Hz, 1H). **^{13}C NMR** (151 MHz, $CDCl_3$) δ/ ppm = 140.4, 138.5, 129.3, 125.4, 125.3, 122.6, 78.4. **HRMS** (EI) calculated for C_8H_5IS: 259.9151 $[M]^+$, found: 259.9149.

These data are in agreement with those reported previously in the literature.[10]

1 C atom missing in the ^{13}C NMR. This is in line with literature reports.

2-Iodothiophene

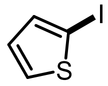

Prepared according to GP 2 at room temperature. The yield was determined by quantitative ^{1}H NMR (97%).

^{1}H NMR (600 MHz, DMF-d_7) δ/ ppm = 7.71 (dd, *J* = 5.4, 1.2 Hz, 1H), 7.39 (dd, *J* = 3.6, 1.2 Hz, 1H), 6.92 (dd, *J* = 5.4, 3.6 Hz, 1H). **^{13}C NMR** (151 MHz, DMF-d_7) δ/ ppm = 156.9, 138.3, 133.4, 130.4. **MS** (70 eV, EI): *m/z* (%): 209.9 (100) $[M]^+$, 126.9 (21), 83.0 (27), 57 (12).

Due to its high volatility, the title compound was not isolated.

3-Iodothiophene

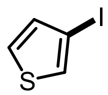

Prepared according to GP 2 at room temperature. The yield was determined by quantitative ^{1}H NMR (97%).

^{1}H NMR (600 MHz, DMF-d_7) δ/ ppm = 7.86 – 7.80 (m, 1H), 7.61 – 7.55 (m, 1H), 7.23 (dd, *J* = 5.0, 1.2 Hz, 1H). **^{13}C NMR** (151 MHz, DMF-d_7) δ/ ppm = 156.9, 135.9, 130.5, 129.6. **MS** (70 eV, EI): *m/z* (%): 209.9 (100) $[M]^+$, 126.9 (10), 83.0 (21).

Due to its high volatility, the title compound was not isolated.

Synthesis of Aryl Germanes

General Procedure 3 (GP 3)

Triethylgermanium chloride (1.05 equiv.) and the corresponding aryl iodide or aryl bromide (1.0 equiv.) were dissolved in anhydrous and degassed THF (0.2 M) under argon, *i*PrMgCl (1.2 M in THF; 1.2 equiv.) was added slowly and the reaction was stirred for 3 h at room temperature (ArI) or for 12 h at 60 °C (ArBr). The reaction was quenched by addition of aqueous solution of NH_4Cl (sat.), the organic phase was separated and the aqueous phase was extracted with DCM (3x). The combined organic phases were dried over $MgSO_4$, the solvent was removed under reduced pressure and the crude product mixture was purified by silica column chromatography.

General Procedure 4 (GP 4)

Triethylgermanium chloride (1.0 equiv.) and the corresponding aryl Grignard reagent (1.1 equiv.) were dissolved in anhydrous and degassed THF (0.2 M) under argon and stirred for 3 h at room temperature. The reaction was quenched by addition of aqueous solution of NH_4Cl (sat.), the organic phase was separated and the aqueous phase was extracted with DCM (3x). The combined organic phases were dried over $MgSO_4$, the solvent was removed under reduced pressure and the crude product mixture was purified by silica column chromatography.

***Note:** Our group meanwhile developed a formal C-H germylation strategy. Synthesis from pre-functionalized arenes (as followed herein) is hence not strictly necessary.*

Triethyl(phenyl)germane

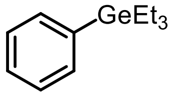

Prepared according to GP 2. The title product was obtained after purification by column chromatography (*n*-pentane) as a colorless oil (629 mg, 2.65 mmol, 89%).

R_f = 0.90 (*n*-pentane). **^{1}H NMR** (600 MHz, $CDCl_3$) δ/ ppm = 7.45 (dd, *J* = 7.6, 1.8 Hz, 2H), 7.37 – 7.30 (m, 3H), 1.12 – 1.03 (m, 9H), 1.03 – 0.95 (m, 6H). **^{13}C NMR** (151 MHz, $CDCl_3$) δ/ ppm = 140.0, 134.1, 128.2, 128.0, 9.1, 4.3. **HRMS** (EI) calculated for $C_{12}H_{20}{}^{74}Ge$: 238.0771 $[M]^+$, found: 238.0772. **IR** (neat): *ν*/ cm^{-1} = 3061 (m), 2948 (s), 2873 (m), 2332 (w), 1458 (m), 1429 (m), 1378 (w), 1304 (w), 1230 (w), 1091 (m), 1014 (s), 966 (m), 695 (s).

Triethyl(*p*-tolyl)germane

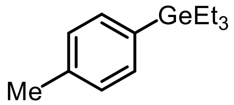

Prepared according to GP 4. The title product was obtained after purification by column chromatography (*n*-pentane) as a colorless oil (575 mg, 2.29 mmol, 76%).

R_f = 0.85 (pentane). **^{1}H NMR** (600 MHz, $CDCl_3$) δ/ ppm = 7.35 (d, *J* = 7.6 Hz, 2H), 7.18 (d, *J* = 7.6 Hz, 2H), 2.36 (s, 3H), 1.11 – 1.03 (m, 9H), 1.01 – 0.95 (m, 6H). **^{13}C NMR** (151 MHz, $CDCl_3$) δ/ ppm = 137.8, 136.0, 133.9, 128.7, 21.4, 8.9, 4.2. **HRMS** (EI) calculated for $C_{13}H_{22}{}^{74}Ge$: 252.0933 $[M]^+$, found: 252.0926. **IR** (neat): *ν*/ cm^{-1} = 3016 (m), 2945 (s), 2732 (w), 2328 (m), 2087 (w), 1897 (m), 1738 (w), 1599 (w), 1455 (s), 1384 (m), 1228 (w), 1188 (w), 1086 (m), 1014 (s), 965 (m), 794 (s), 697 (s).

These data are in agreement with those reported previously in the literature.[12]

Triethyl(4-fluorophenyl)germane

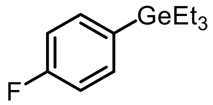

Prepared according to GP 4. The title product was obtained after purification by column chromatography (*n*-pentane) as a colorless oil (732 mg, 2.87 mmol, 96%).

R_f = 0.85 (*n*-pentane). **^{1}H NMR** (600 MHz, $CDCl_3$) δ/ ppm = 7.39 (dd, *J* = 7.9, 1.6 Hz, 2H), 7.05 (dd, *J* = 8.8, 8.8 Hz, 2H), 1.12 – 1.01 (m, 9H), 1.01 – 0.92 (m, 6H). **^{13}C NMR** (151 MHz, $CDCl_3$) δ/ ppm = 163.4 (d, *J* = 246.4 Hz), 135.7 (d, *J* = 6.9 Hz), 135.0 (d, *J* = 3.7 Hz), 115.1 (d, *J* = 19.1 Hz), 9.0, 4.4. **^{19}F NMR** (564 MHz, $CDCl_3$) δ/ ppm = -113.75 – -113.84 (m). **HRMS** (EI) calculated for $C_{12}H_{19}F^{74}Ge$: 256.0683 $[M]^+$, found: 256.0673. **IR** (neat): *ν*/ cm^{-1} = 3029 (w), 2949 (s), 2875 (s), 2332 (w), 2162 (w), 2092 (w), 1890 (w), 1752 (w), 1636 (w), 1584 (s), 1495 (s), 1459 (m), 1380 (w), 1304 (w), 1226 (s), 1160 (s), 1082 (m), 1014 (s), 965 (m), 816 (s), 698 (s).

These data are in agreement with those reported previously in the literature.[12]

Triethyl(4-methoxyphenyl)germane

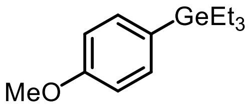

Prepared according to GP 4. The title product was obtained after purification by column chromatography (*n*-pentane) as a colorless oil (785 mg, 2.94 mmol, 98%).

R$_f$ = 0.42 (*n*-pentane). **^{1}H NMR** (600 MHz, CDCl$_3$) δ/ ppm = 7.36 (d, *J* = 8.6 Hz, 2H), 6.91 (d, *J* = 8.6 Hz, 2H), 3.81 (s, 3H), 1.10 – 1.02 (m, 9H), 1.00 – 0.93 (m, 6H). **^{13}C NMR** (151 MHz, CDCl$_3$) δ/ ppm = 159.9, 135.2, 130.5, 113.8, 55.1, 9.1, 4.4. **HRMS** (EI) calculated for $C_{13}H_{22}{}^{74}GeO$: 268.0882 [M]$^+$, found: 268.0890. **IR** (neat): *ν*/ cm^{-1} = 2946 (s), 2326 (w), 2073 (w), 1887 (w), 1731 (w), 1590 (s), 1497 (s), 1457 (s), 1387 (w), 1276 (s), 1243 (s), 1178 (s), 1091 (s), 1024 (s), 964 (m), 810 (m), 697 (s).

These data are in agreement with those reported previously in the literature.[12]

Triethyl(thiophen-2-yl)germane

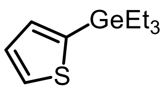

Prepared according to GP 4. The title product was obtained after purification by column chromatography (*n*-hexane/EtOAc, 5:1) as a colorless oil (589 mg, 2.43 mmol, 81%).

R$_f$ = 0.91 (*n*-hexane/ EtOAc, 5:1). **^{1}H NMR** (400 MHz, CDCl$_3$) δ/ ppm = 7.59 (dd, *J* = 4.6, 0.9 Hz, 1H), 7.22 (dd, *J* = 4.6, 3.3 Hz, 1H), 7.18 (dd, *J* = 3.3, 0.9 Hz, 1H), 1.14 – 0.99 (m, 15H). **^{13}C NMR** (101 MHz, CDCl$_3$) δ/ ppm = 137.6, 133.4, 129.7, 127.9, 9.0, 5.7. **HRMS** (EI) calculated for $C_{10}H_{18}{}^{74}GeS$: 244.0336 [M]$^+$, found: 244.0342. **IR** (neat): *ν*/ cm^{-1} = 3070 (w), 2951 (s), 2907 (s), 2872 (s), 2330 (w), 2101 (w), 1593 (w), 1497 (w), 1458 (m), 1425 (m), 1404 (w), 1379 (w), 1323 (w), 1212 (m), 1078 (w), 1016 (s), 965 (s), 846 (m), 823 (m), 746 (w), 698 (s).

These data are in agreement with those reported previously in the literature.[12]

Triethyl(4-iodophenyl)germane

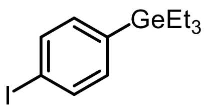

Prepared according to GP 3. The title product was obtained after purification by column chromatography (*n*-pentane) as a colorless oil. (963 mg, 2.65 mmol, 88%).

R$_f$ = 0.90 (*n*-pentane). **^{1}H NMR** (600 MHz, CDCl$_3$) δ/ ppm = 7.67 (d, *J* = 8.0 Hz, 2H), 7.16 (d, *J* = 8.0 Hz, 2H), 1.09 – 1.01 (m, 9H), 1.01 – 0.93 (m, 6H). **^{13}C NMR** (151 MHz, CDCl$_3$) δ/ ppm = 139.4, 137.0, 135.9, 95.0, 9.0, 4.2. **HRMS** (EI) calculated for $C_{12}H_{19}{}^{74}GeI$: 363.9738 [M]$^+$, found: 363.9744. **IR** (neat): *ν*/ cm^{-1} = 3060 (w), 2948 (s), 2872 (s), 2734 (w), 2329 (w), 2097 (w), 1900 (w), 1741 (w), 1631 (w), 1561 (m), 1463 (m), 1426 (m), 1372 (m), 1228 (w), 1051 (w), 1014 (s), 966 (m), 797 (s), 696 (s).

These data are in agreement with those reported previously in the literature.[13]

Triethyl(4-bromophenyl)germane

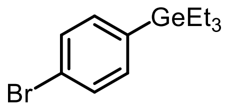

Prepared according to GP 3. The title product was obtained after purification by column chromatography (*n*-pentane) as a colorless oil (820 mg, 2.61 mmol, 87%).

R_f = 0.90 (*n*-pentane). **^{1}H NMR** (600 MHz, $CDCl_3$) δ/ ppm = 7.47 (d, *J* = 8.1 Hz, 2H), 7.29 (d, *J* = 8.1 Hz, 2H), 1.09 – 1.01 (m, 9H), 1.01 – 0.94 (m, 6H). **^{13}C NMR** (151 MHz, $CDCl_3$) δ/ ppm = 138.8, 135.7, 131.1, 123.0, 9.0, 4.3. **HRMS** (EI) calculated for $C_{12}H_{19}{}^{79}Br^{74}Ge$: 315.9882 $[M]^+$, found: 315.9867. **IR** (neat): *ν*/ cm^{-1} = 3068 (w), 2949 (s), 2872 (s), 2328 (w), 2113 (w), 1993 (w), 1900 (w), 1742 (w), 1633 (w), 1566 (m), 1464 (s), 1427 (m), 1374 (m), 1228 (w), 1061 (m), 1009 (s), 866 (m), 801 (s), 701 (s).

These data are in agreement with those reported previously in the literature.[13]

3,5-Dimethyl-4-(triethylgermyl)isoxazole

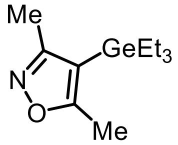

Prepared according to GP 3. The title product was obtained after purification by column chromatography (DCM) as a colorless oil (638 mg, 2.49 mmol, 83%).

R_f = 0.83 (DCM). **^{1}H NMR** (400 MHz, $CDCl_3$) δ/ ppm = 2.36 (s, 3H), 2.23 (s, 3H), 1.08 – 0.94 (m, 15H). **^{13}C NMR** (101 MHz, $CDCl_3$) δ/ ppm = 173.0, 163.8, 105.2, 13.2, 12.8, 8.9, 4.7. **HRMS** (EI) calculated for $C_{11}H_{21}{}^{74}GeNONa$: 280.0727 $[M+Na]^+$, found: 280.0725. **IR** (neat): *ν*/ cm^{-1} = 3467 (w), 2953 (s), 2873 (s), 2735 (w), 2184 (w), 1578 (s), 1457 (s), 1397 (s), 1351 (s), 1239 (m), 1093 (m), 1014 (s), 971 (m), 901 (w), 803 (w), 754 (m), 705 (s), 579 (s).

Triethyl(4-chlorophenyl)germane

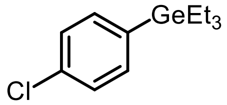

Prepared according to GP 3. The title product was obtained after purification by column chromatography (*n*-pentane) as a colorless oil (797 mg, 2.94 mmol, 98%).

R_f = 0.87 (*n*-pentane). **^{1}H NMR** (600 MHz, $CDCl_3$) δ/ ppm = 7.36 (d, *J* = 8.2 Hz, 2H), 7.31 (d, *J* = 8.2 Hz, 2H), 1.08 – 1.02 (m, 9H), 1.01 – 0.94 (m, 6H). **^{13}C NMR** (151 MHz, $CDCl_3$) δ/ ppm = 138.2, 135.4, 134.5, 128.2, 9.0, 4.3. **HRMS** (EI) calculated for $C_{12}H_{19}{}^{35}Cl^{74}Ge$: 272.0387 $[M]^+$, found: 272.0385. **IR** (neat): *ν*/ cm^{-1} = 3070 (w), 2949 (s), 2873 (s), 2332 (w), 2150 (w), 2091 (w), 2030 (w), 1900 (w) 1637 (w), 1572 (m), 1468 (s), 1428 (m), 1378 (m), 1304 (w), 1229 (w), 1075 (s), 1012 (s), 966 (m), 806 (s), 699 (s).

These data are in agreement with those reported previously in the literature.[13]

Triethyl(2-methoxyphenyl)germane

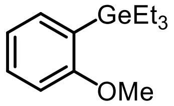

Prepared according to GP 3. The title product was obtained after purification by column chromatography (*n*-pentane) as a colorless oil (689 mg, 2.58 mmol, 86%). $\mathbf{R_f}$ = 0.68 (*n*-pentane). **^{1}H NMR** (600 MHz, $CDCl_3$) δ/ ppm = 7.34 – 7.29 (m, 2H), 6.95 (dd, *J* = 7.2, 7.2 Hz, 1H), 6.83 (d, *J* = 8.4 Hz, 1H), 3.79 (s, 3H), 1.09 – 0.97 (m, 15H). **^{13}C NMR** (151 MHz, $CDCl_3$) δ/ ppm = 163.6, 135.3, 129.9, 127.7, 120.6, 109.5, 55.1, 9.2, 4.7. **HRMS** (EI) calculated for $C_{13}H_{22}{}^{74}GeO$: 268.0882 $[M]^+$, found: 268.0873. **IR** (neat): *ν*/ cm^{-1} = 2945 (s), 2871 (m), 2834 (m), 2331 (w), 2159 (w), 1739 (w), 1578 (s), 1458 (s), 1427 (s), 1235 (s), 1169 (w), 1014 (m), 966 (w), 840 (w), 769 (s), 707 (s).
These data are in agreement with those reported previously in the literature.[14]

Triethyl(mesityl)germane

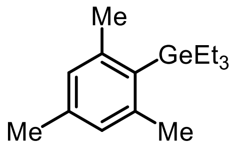

Prepared according to GP 3. The title product was obtained after purification by column chromatography (DCM) as a colorless oil (839 mg, 3.00 mmol, 99%).
$\mathbf{R_f}$ = 0.99 (DCM). **^{1}H NMR** (600 MHz, $CDCl_3$) δ/ ppm = 6.81 (s, 2H), 2.38 (s, 6H), 2.25 (s, 3H), 1.14 – 1.07 (m, 6H), 1.07 – 1.02 (m, 9H). **^{13}C NMR** (151 MHz, $CDCl_3$) δ/ ppm = 144.4, 137.8, 134.2, 128.8, 24.5, 21.0, 9.4, 8.0. **HRMS** (EI) calculated for $C_{15}H_{26}{}^{74}Ge$: 280.1241 $[M]^+$, found: 280.1241. **IR** (neat): *ν*/ cm^{-1} = 3468 (w), 2952 (s), 2871 (s), 2730 (w), 2395 (w), 2182 (w), 1720 (w), 1603 (m), 1550 (m), 1456 (s), 1381 (s), 1288 (w), 1236 (w), 1011 (s), 970 (m), 846 (m), 706 (s), 569 (s).

Triethyl(3,4,5-trimethoxyphenyl)germane

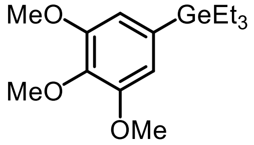

Prepared according to GP 3. The title product was obtained after purification by column chromatography (*n*-pentane/Et_2O 5:1) as a yellow oil (970 mg, 2.97 mmol, 99%).
$\mathbf{R_f}$ = 0.64 (*n*-pentane/Et_2O 5:1). **^{1}H NMR** (600 MHz, $CDCl_3$) δ/ ppm = 6.61 (s, 2H), 3.88 (s, 6H), 3.86 (s, 3H), 1.08 (t, *J* = 8.4 Hz, 9H), 1.02 – 0.95 (m, 6H). **^{13}C NMR** (151 MHz, $CDCl_3$) δ/ ppm = 153.1, 138.4, 135.1, 110.5, 60.9, 56.3, 9.1, 4.5. **HRMS** (EI) calculated for $C_{15}H_{26}{}^{74}GeO_3$: 351.0986 $[M+Na]^+$, found: 351.0996. **IR** (neat): *ν*/ cm^{-1} = 2945 (s), 2833 (w), 1735 (w), 1570 (s),

1498 (m), 1457 (m), 1392 (s), 1300 (s), 1239 (m), 1180 (w), 1118 (s), 1011 (s), 967 (w), 922 (w), 822 (m), 769 (w), 698 (s).

Triethyl(naphthalen-1-yl)germane

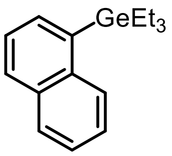

Prepared according to GP 3. The title product was obtained after purification by column chromatography (*n*-hexane) as a colorless oil (743 mg, 2.59 mmol, 86%). **R$_f$** = 0.88 (*n*-hexane). **^{1}H NMR** (400 MHz, $CDCl_3$) δ/ ppm = 8.00 – 7.95 (m, 1H), 7.89 – 7.80 (m, 2H), 7.60 (dd, *J* = 6.7, 1.3 Hz, 1H), 7.53 – 7.41 (m, 3H), 1.25 – 1.14 (m, 6H), 1.13 – 1.02 (m, 9H). **^{13}C NMR** (101 MHz, $CDCl_3$) δ/ ppm = 138.5, 137.6, 133.7, 133.3, 129.1, 128.9, 128.3, 125.7, 125.4, 125.3, 9.3, 5.7. **HRMS** (EI) calculated for $C_{16}H_{22}{}^{74}Ge$: 288.0928 $[M]^+$, found: 288.0931. **IR** (neat): *ν*/ cm^{-1} = 3850 (w), 3052 (m), 2948 (s), 2872 (s), 2731 (w), 2660 (w), 2325 (m), 2103 (w), 1994 (w), 1930 (w), 1809 (w), 1588 (w), 1504 (m), 1457 (m), 1379 (m), 1322 (w), 1221 (w), 1138 (m), 1013 (s), 967 (s), 855 (w), 785 (s), 701 (s).
These data are in agreement with those reported previously in the literature.[12]

Triethyl(4-(4,4,5,5-tetramethyl-1,3,2-dioxaborolan-2-yl)phenyl)germane

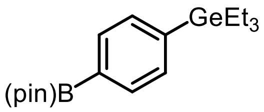

Triethyl(4-iodophenyl)germane (1.09 g, 3.0 mmol, 1.0 equiv.) was added to a round bottom flask and dissolved in anhydrous and degassed THF (20 mL) under argon. *i*PrMgCl (2.0 M in THF, 1.8 mL, 3.6 mmol, 1.2 equiv.) was added dropwise at 0 °C and the reaction was stirred for 30 min. Tris *iso*-propylborate (1.0 M in THF, 3.3 mL, 3.3 mmol, 1.2 equiv.) was added and the reaction was stirred at room temperature for 12 h. The solvent was removed *in vacuo* and anhydrous toluene (20 mL) and pinacol (1.42 g, 12.0 mmol, 4.0 equiv.) were added. The reaction was stirred for 8 h under reflux. It was quenched by addition of aqueous solution of NH_4Cl (sat.), the organic phase was separated and the aqueous phase was extracted with DCM (3x20 mL). The combined organic phases were dried over $MgSO_4$ and the solvent was removed under reduced pressure. The title product was obtained after purification by column chromatography (*n*-hexane/ EtOAc, 50:1) as a white solid (496 mg, 1.34 mmol, 45%).
R$_f$ = 0.47 (*n*-hexane/ EtOAc, 50:1). **M.p.** = 55.6 °C. **^{1}H NMR** (600 MHz, $CDCl_3$) δ/ ppm = 7.77 (d, *J* = 7.7 Hz, 2H), 7.45 (d, *J* = 7.7 Hz, 2H), 1.34 (s, 12H), 1.08 – 1.02 (m, 9H), 1.01 – 0.95 (m, 6H). **^{13}C NMR** (151 MHz, $CDCl_3$) δ/ ppm = 144.1, 136.4, 134.0, 133.5, 83.8, 25.0, 9.1, 4.2. **HRMS** (EI) calculated for $C_{18}H_{31}B^{74}GeO_2$: 364.1623 $[M]^+$, found: 364.1636. **IR** (neat): *ν*/ cm^{-1} = 3057 (w), 2953 (m), 2875 (m), 1597 (m), 1503 (w), 1459 (w), 1355 (s), 1298 (m), 1270 (w), 1212 (w), 1143 (s), 1107 (w), 1064 (s), 1017 (m), 962 (m), 857 (s), 818 (m), 736 (w), 696 (s), 659 (m).

These data are in agreement with those reported previously in the literature.[12]

Trimethyl(4-(triethylgermyl)phenyl)silane

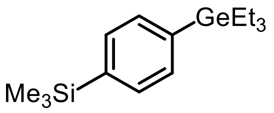

Triethyl(4-iodophenyl)germane (1.09 g, 3.0 mmol, 1.0 equiv.) was added to a round bottom flask and dissolved in anhydrous and degassed THF (20 mL) under argon. *i*PrMgCl (2.0 M in THF, 1.8 mL, 3.6 mmol, 1.2 equiv.) was added dropwise at 0 °C and the reaction was stirred for 30 min. Tetramethyl orthosilicate (899 µL, 6.0 mmol, 2.0 equiv.) was added and the mixture was stirred at room temperature for 12 h. The reaction was quenched by addition of aqueous solution of NH_4Cl (sat.), the organic phase was separated and the aqueous phase was extracted with DCM (3x20 mL). The combined organic phases were dried over $MgSO_4$ and the solvent was removed under reduced pressure. The title product was obtained after purification by column chromatography (*n*-hexane) as a colorless oil (576 mg, 1.86 mmol, 62%).

R_f = 0.89 (*n*-hexane). **^{1}H NMR** (600 MHz, $CDCl_3$) δ/ ppm = 7.49 (d, *J* = 7.7 Hz, 2H), 7.43 (d, *J* = 7.7 Hz, 2H), 1.10 – 1.04 (m, 9H), 1.02 – 0.94 (m, 6H), 0.26 (s, 9H). **^{13}C NMR** (151 MHz, $CDCl_3$) δ/ ppm = 141.1, 137.9, 133.6, 133.5, 9.1, 4.3, -2.3. **HRMS** (EI) calculated for $C_{15}H_{28}Si^{74}Ge$: 310.1172 $[M]^+$, found: 310.1180. **IR** (neat): *ν*/ cm^{-1} = 3048 (w), 2951 (s), 2873 (m), 2328 (w), 2113 (w), 1991 (w), 1914 (w), 1584 (w), 1458 (m), 1426 (w), 1377 (m), 1247 (s), 1122 (m), 1014 (m), 967 (m), 839 (s), 800 (m), 754 (m), 697 (s).

These data are in agreement with those reported previously in the literature.[12]

Triethyl(4-(tributylstannyl)phenyl)germane

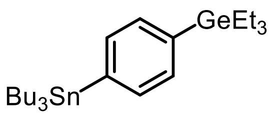

Triethyl(4-iodophenyl)germane (276 mg, 0.76 mmol, 1.0 equiv.) was added to a round bottom flask and dissolved in anhydrous and degassed THF (3 mL) under argon. *n*BuLi (2.5 M in toluene, 0.46 mL, 1.14 mmol, 1.5 equiv.) was added dropwise at -78 °C and the reaction was stirred for 30 min. Tributyltin chloride (227 µL, 0.836 mmol, 1.1 equiv.) was added and the mixture was stirred while warming to room temperature for 12 h. The reaction was quenched by addition of aqueous solution of NH_4Cl (sat.), the organic phase was separated and the aqueous phase was extracted with DCM (3x20 mL). The combined organic phases were dried over $MgSO_4$ and the solvent was removed under reduced pressure. The title product was obtained after purification by column chromatography (*n*-hexane) as a colorless oil (132 mg, 0.251 mmol, 33%).

R_f = 0.90 (*n*-hexane). **^{1}H NMR** (600 MHz, $CDCl_3$) δ/ ppm = 7.45 – 7.42 (m, 2H), 7.41 – 7.37 (m, 2H), 1.59 – 1.52 (m, 6H), 1.38 – 1.30 (m, 6H), 1.10 – 1.03 (m, 15H), 1.02 – 0.96 (m, 6H), 0.90 (t, *J* = 7.4 Hz,

9H). **^{13}C NMR** (151 MHz, $CDCl_3$) δ/ ppm = 141.9, 139.5, 136.1, 133.6, 29.3, 27.6, 13.8, 9.7, 9.1, 4.3. **MS** (70 eV, EI): *m/z* (%): 473.1 (14), 472.0 (10), 471.1 (40), 470.1 (25) $[M\text{-}Et]^+$, 469.1 (50), 468.1 (27), 467.1 (46), 466.1 (16), 465.1 (22), 415.0 (11), 413.0 (13), 411.0 (12), 360.9 (13), 358.9 (36), 358 (19), 356.9 (48), 355.9 (24), 355 (50), 353.9 (14), 352.9 (26), 350.9 (11), 270.8 (12), 268.8 (13), 266.8 (12), 211.0 (12), 209.0 (54), 208.0 (28), 207.0 (100), 205.0 (26), 196.9 (12), 194.9 (10), 192.9 (19), 191.9 (13l), 190.9 (18), 163.9 (12), 162.8 (15), 154.9 (12), 152.9 (59), 151.9 (19), 150.9 (79), 149.9 (24), 148.9 (66), 148.0 (11), 146.9 (29), 135.0 (11), 132.9 (10), 73.0 (15).

Triethyl(thiophen-3-yl)germane

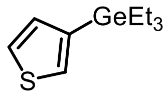

Prepared according to GP 3. The title product was obtained after purification by column chromatography (DCM) as a colorless oil (625 mg, 2.57 mmol, 86%).

R_f = 0.95 (DCM). **^{1}H NMR** (400 MHz, $CDCl_3$) δ/ ppm = 7.41 (dd, *J* = 4.8, 2.6 Hz, 1H), 7.33 (dd, *J* = 2.6, 1.1 Hz, 1H), 7.14 (dd, *J* = 4.8, 1.1 Hz, 1H), 1.12 – 1.03 (m, 9H), 1.03 – 0.93 (m, 6H). **^{13}C NMR** (101 MHz, $CDCl_3$) δ/ ppm = 138.4, 131.7, 129.9, 125.2, 9.1, 5.0. **HRMS** (EI) calculated for $C_{10}H_{18}{}^{74}GeS$: 244.0336 $[M]^+$, found: 244.0335. **IR** (neat): ν/ cm^{-1} = 3063 (w), 2950 (s), 2907 (s), 2872 (s), 2829 (m), 2733 (w), 2327 (w), 2169 (w), 2099 (w), 1755 (w), 1570 (w), 1458 (s), 1426 (m), 1375 (m), 1337 (w), 1229 (w), 1199 (m), 1089 (s), 1015 (s), 967 (m), 846 (s), 798 (w), 765 (s), 695 (s).

These data are in agreement with those reported previously in the literature.[12]

Benzo[b]thiophen-3-yltriethylgermane

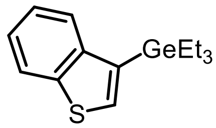

Prepared according to GP 3. The title product was obtained after purification by column chromatography (DCM) as a colorless oil (638 mg, 2.49 mmol, 83%).

R_f = 0.83 (DCM). **^{1}H NMR** (400 MHz, $CDCl_3$) δ/ ppm = 7.98 – 7.92 (m, 1H), 7.89 – 7.84 (m, 1H), 7.42 (s, 1H), 7.40 – 7.30 (m, 2H), 1.20 – 1.05 (m, 15H). **^{13}C NMR** (101 MHz, $CDCl_3$) δ/ ppm = 144.0, 141.4, 131.7, 125.4, 124.5, 124.0, 123.9, 122.8, 9.2, 5.1. **HRMS** (EI) calculated for $C_{14}H_{20}{}^{74}GeS$: 294.0497 $[M]^+$, found: 294.0492. **IR** (neat): ν/ cm^{-1} = 3057 (w), 2950 (s), 2907 (s), 2871 (s), 2828 (w), 2733 (w), 2329 (w), 2086 (w), 1571 (w), 1455 (s), 1415 (s), 1379 (m), 1307 (m), 1253 (m), 1172 (w), 1134 (w), 1062 (m), 1015 (s), 966 (m), 938 (m), 850 (w), 810 (m), 782 (m), 752 (s), 725 (s), 699 (s).

(2,6-Dimethoxyphenyl)triethylgermane

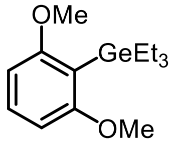

Prepared according to GP 3. The title product was obtained after purification by column chromatography (*n*-hexane) as a colorless oil (451 mg, 1.52 mmol, 51%). $\mathbf{R_f}$ = 0.35 (*n*-hexane). **^{1}H NMR** (600 MHz, $CDCl_3$) δ/ ppm = 7.24 (t, *J* = 8.1 Hz, 1H), 6.49 (d, *J* = 8.1 Hz, 2H), 3.74 (s, 6H), 1.06 – 0.98 (m, 15H). **^{13}C NMR** (151 MHz, $CDCl_3$) δ/ ppm = 165.0, 130.6, 114.4, 103.4, 55.3, 9.4, 6.7. **HRMS** (ESI) calculated for $C_{14}H_{24}{}^{74}GeO_2$: 299.1061 $[M+H]^+$, found: 299.1054. **IR** (neat): ν/ cm^{-1} = 3085 (w), 2946 (m), 2870 (m), 2833 (w), 2329 (w), 1578 (s), 1458 (s), 1426 (s), 1376 (w), 1297 (w), 1236 (s), 1171 (w), 1020 (s), 1013 (m), 970 (w), 837 (w), 770 (s), 742 (m), 709 (s).

Halolabeling of Physiologically Relevant Target Molecule

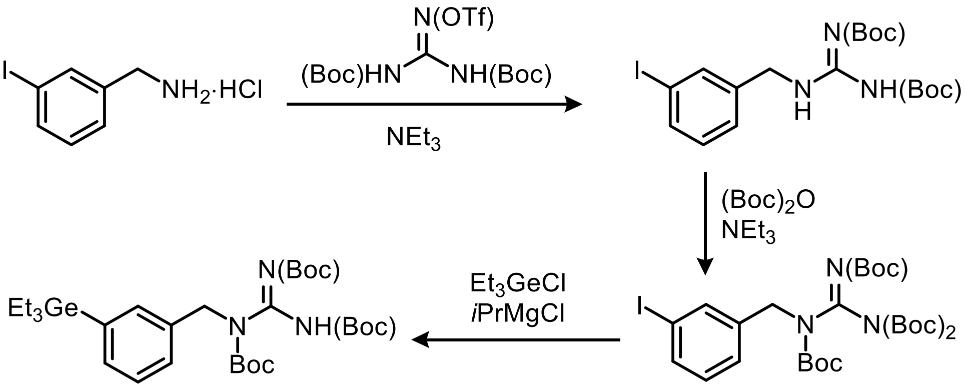

tert-Butyl{(Z)-[(3-iodobenzyl)amino][(tert-butoxycarbonyl)amino]methylidene} carbamate

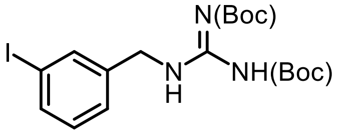

3-Iodobenzylamine hydrochloride (472 mg, 1.75 mmol, 1.0 equiv.) and NEt_3 (0.97 mL, 7.00 mmol, 4.0 equiv.) were dissolved in DCM (17.5 mL) and and stirred for 5 minutes. *N*,*N'*-bis(*tert*-butoxycarboyl)-*N''*-triflylguanidine (685 mg, 1.75 mmol, 1.0 equiv.) was added and the reaction was stirred for further 30 minutes. The solvent was removed *in vacuo*, H_2O was added and the organic phase was separated. After extracting the aqueous phase with DCM (3x), the combined organic layers were washed with water and brine before drying over $MgSO_4$. The solvent was removed *in vacuo* to give the title product as a white solid (827 mg, 1.74 mmol, 99%).
^{1}H NMR (600 MHz, $CDCl_3$) δ/ ppm = 11.53 (s, 1H), 8.58 (s, 1H), 7.66 (s, 1H), 7.64 – 7.59 (m, 1H), 7.30 – 7.24 (m, 1H), 7.08 (dd, *J* = 7.8 Hz, 1H), 4.57 (d, *J* = 5.3 Hz, 2H), 1.51 (s, 9H), 1.49 (s, 9H). **^{13}C NMR** (151 MHz, $CDCl_3$) δ/ ppm = 163.7, 156.3, 153.3, 139.9, 137.1, 136.9, 130.6, 127.2, 94.7, 83.5, 79.7, 44.2, 28.4, 28.2. **HRMS** (ESI) calculated for $C_{16}H_{26}IN_3O_4Na$: 498.0860 $[M+Na]^+$, found: 498.0852.
These data are in agreement with those reported previously in the literature.[15]

tert-Butyl-N-[(1Z)-{bis[(tert-butoxy)carbonyl]amino}({[(tert-butoxy)carbonyl][(3-iodophenyl) methyl]amino})methylidene]carbamate

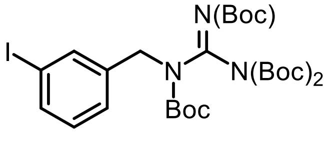

Di-*tert*-butyl dicarbonate (1.50 g, 6.68 mmol, 4.0 equiv.), dimethyl amino pyridine (612 mg, 5.01 mmol, 3.0 equiv.), NEt_3 (0.93 mL, 6.68 mmol, 4.0 equiv.) and *tert*- butyl {(Z)-[(3-iodobenzyl) amino] [(*tert*-butoxy- carbonyl)amino] methylidene} carbamate (794 mg, 1.67 mmol, 1.0 equiv.) were dissolved in THF (25 mL) and stirred for 17 h at room temperature. The solvent was

removed *in vacuo* and the title product was purified by column chromatography (*n*-pentane/EtOAc 7:1 to 4:1) as a colorless oil (801 mg, 1.19 mmol, 71%).

R_f = 0.13 (*n*-pentane/EtOAc 9:1). **^{1}H NMR** (600 MHz, $CDCl_3$) δ/ ppm = 7.74 (s, 1H), 7.58 (d, *J* = 7.9 Hz, 1H), 7.39 (d, *J* = 7.8 Hz, 1H), 7.02 (dd, *J* = 7.8 Hz, 1H), 4.96 (s, 2H), 1.49 (s, 9H), 1.46 (s, 18H), 1.41 (s, 9H). **^{13}C NMR** (151 MHz, $CDCl_3$) δ/ ppm = 157.4, 151.2, 147.4, 144.5, 140.0, 136.9, 136.4, 130.1, 127.4, 94.1, 84.2, 83.9, 82.2, 49.5, 28.1, 28.03, 28.00. **HRMS** (ESI) calculated for $C_{28}H_{42}IN_3O_8Na$: 986.1909 $[M+Na]^+$, found: 698.1906.

These data are in agreement with those reported previously in the literature.[15]

tert-Butyl-N-[(1Z)-{[(_tert_-butoxy)carbonyl]amino}({[(_tert_-butoxy)carbonyl][(3-(triethylgermanium)phenyl) methyl]amino})methylidene]carbamate

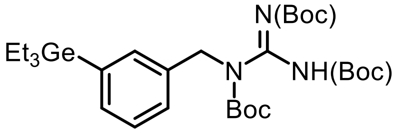

Prepared according to GP 3. The title product was obtained after purification by column chromatography (*n*-pentane/EtOAc 6:1 to 3:1) as a colorless oil (359 mg, 0.590 mmol, 50%).

R_f = 0.45 (*n*-pentane/EtOAc 9:1). **M.p.** = 103-104 °C. **^{1}H NMR** (600 MHz, DMF-d_7) δ/ ppm = 10.56 (s, 1H), 7.56 – 7.51 (m, 1H), 7.39 – 7.30 (m, 3H), 4.90 (s, 2H), 1.47 (s, 9H), 1.46 (s, 9H), 1.38 (s, 9H), 1.08 – 0.97 (m, 15H). **^{13}C NMR** (151 MHz, DMF-d_7) δ/ ppm = 153.3, 150.9, 149.7, 139.4, 138.1, 133.4, 132.9, 128.0, 127.7, 82.9, 81.6, 79.5, 51.0, 27.9, 27.6, 8.8, 4.1. **HRMS** (ESI) calculated for $C_{29}H_{49}O_6N_3GeK$: 648.2465 $[M+K]^+$, found: 648.2459.

tert-Butyl{(Z)-[(3-bromobenzyl)amino][(_tert_-butoxycarbonyl)amino]methylidene} carbamate ([Br]MIBG)

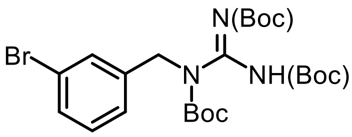

Prepared according to GP 1 at 60°C for 2 h. The yield was determined by quantitative ^{1}H NMR (95%). The title product was obtained by column chromatography (*n*-pentane/Et_2O 8:1 to 4:1) as a white solid (155 mg, 0.294 mmol, 81%).

R_f = 0.31 (*n*-pentane/Et_2O 8:1 to 4:1). **^{1}H NMR** (600 MHz, $CDCl_3$) δ/ ppm = 10.67 (s, 1H), 7.54 (s, 1H), 7.36 (d, *J* = 8.0 Hz, 1H), 7.32 (d, *J* = 7.8 Hz, 1H), 7.16 (dd, *J* = 7.8 Hz, 1H), 4.97 (s, 2H), 1.50 (s, 18H), 1.37 (s, 9H). **^{13}C NMR** (151 MHz, $CDCl_3$) δ/ ppm = 153.3, 152.7, 140.4, 131.1, 130.3, 130.0, 126.6, 122.3, 84.0, 50.3, 28.2, 27.9. **HRMS** (ESI) calculated for $C_{23}H_{34}BrN_3O_6Na$: 550.1523 $[M+Na]^+$, found: 550.1530.

Not all quaternary carbon atoms are fully visible in the ^{13}C NMR analysis.

Performing the bromination according to GP 3 at 60 °C for 30 min yielded 95% of the title compound (quantified by ^{1}H NMR analysis using ethylene carbonate as internal standard).
Performing the bromination according to GP 3 at 80 °C for 60 min with NaBr (10.3 mg, 0.1 mmol, 2.0 equiv.) and NCS (20.0 mg, 0.1 mmol, 2.0 equiv.) to form Br^{+} *in situ* yielded 78% of the title compound (quantified by ^{1}H NMR analysis using ethylene carbonate as internal standard).

tert-Butyl-N-[(1Z)-{bis-[(tert-butoxy)carbonyl]amino}({[(tert-butoxy)carbonyl][(3-(triethylgermanium)-phenyl) methyl]amino})methylidene]carbamate

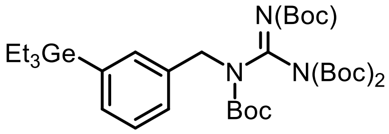

Di-*tert*-butyl dicarbonate (392 mg, 1.80 mmol, 3.0 equiv.), dimethyl amino pyridine (183 mg, 1.50 mmol, 2.5 equiv.), NEt_3 (0.25 mL, 1.80 mmol, 3.0 equiv.) and *tert*-butyl-N-[(1Z)-{[(*tert*-butoxy) carbonyl] amino}({[(*tert*-butoxy) carbonyl] [(3-(triethylgermanium) phenyl) methyl] amino}) methylidene] carbamate (364 mg, 0.60 mmol, 1.0 equiv.) were dissolved in THF (15 mL) and stirred for 17 h at room temperature. The solvent was removed *in vacuo* and the title product was purified by column chromatography (*n*-hexane/Et_2O 4:1) as a colorless oil (993 mg, 1.40 mmol, 78%).
R_f = 0.41 (*n*-hexane /Et_2O 4:1). **^{1}H NMR** (600 MHz, $CDCl_3$) δ/ ppm = 7.47 (s, 1H), 7.42 (d, *J* = 7.4 Hz, 1H), 7.29 (d, *J* = 7.0 Hz, 1H), 7.24 (dd, *J* = 7.4 Hz, 1H), 5.03 (s, 2H), 1.48 (s, 9H), 1.42 (s, 18H), 1.40 (s, 9H), 1.07 – 0.94 (m, 15H). **^{13}C NMR** (151 MHz, $CDCl_3$) δ/ ppm = 157.7, 151.5, 147.5, 144.5, 139.6, 136.8, 134.2, 133.0, 128.0, 127.8, 83.71, 83.69, 82.0, 50.3, 28.1, 28.00, 27.95, 9.1, 4.3. **HRMS** (ESI) calculated for $C_{34}H_{57}N_3O_8GeNa$: 732.3250 $[M+Na]^+$, found: 732.3257.

tert-Butyl-N-[(1Z)-{bis-[(tert-butoxy)carbonyl]amino}({[(tert-butoxy)carbonyl][(3-iodo-phenyl) methyl]amino})methylidene]carbamate

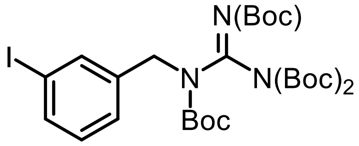

Prepared according to GP 2 at 60°C for 1 h. The yield was determined by quantitative ^{1}H NMR (96%). The title product was obtained by column chromatography (*n*-pentane/EtOAc 7:1 to 4:1) as a colorless oil (57.2 mg, 0.085 mmol, 85%).
The characterization data matches the one previously reported in this manuscript (see S25).

Tolerance and Orthogonality of Halogenation Approaches

Tolerance of Aryl Iodides in Fluorination Approaches

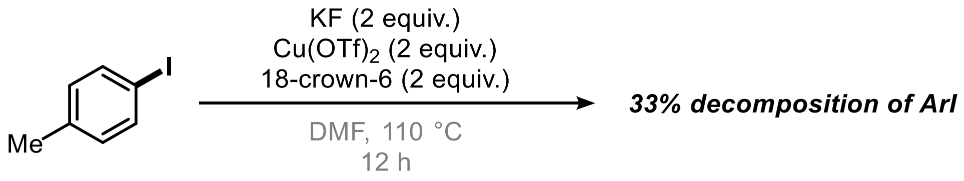

1-Iodo-4-methylbenzene (6.5 µL, 0.05 mmol, 1 equiv.), KF (5.8 mg, 0.10 mmol, 2 equiv.), copper(II) triflate (36.2 mg, 0.10 mmol, 2 equiv.) and 18-crown-6 (26.3 mg, 0.10 mmol, 2 equiv.) were dissolved in DMF (0.5 mL) and stirred for 12 h at 110 °C. The consumption of aryl iodide was determined by calibrated GC-MS analysis (using mesitylene as internal standard).

Compatibility of Functional Handles in Halogenation Approaches

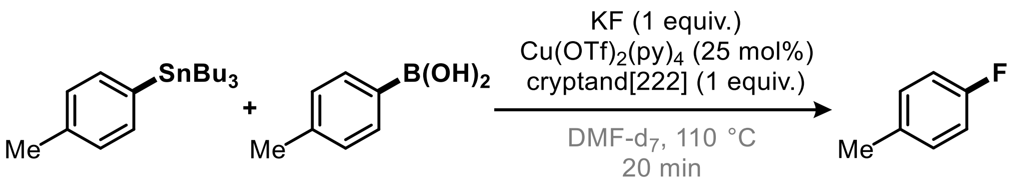

To test the intermolecular tolerance of the $SnBu_3$- and $B(OH)_2$-site towards an established fluorination approach, tributyl(*p*-tolyl)stannane (19.1 mg, 0.05 mmol, 1 equiv.), *p*-tolylboronic acid (6.8 mg, 0.05 mmol, 1 equiv.), KF (2.9 mg, 0.05 mmol, 1 equiv.), cryptand[222] (18.8 mg, 0.05 mmol, 1 equiv.) and copper(II) triflate (8.5 mg, 0.0125 mmol, 0.25 equiv.) were dissolved in DMF-d_7 (0.3 mL) and stirred for 20 min at 110 °C. The reaction mixture was analyzed by quantitative ^{1}H NMR (using 1,4-difluorobenzene as internal standard). The analysis revealed full consumption of both reaction partners.

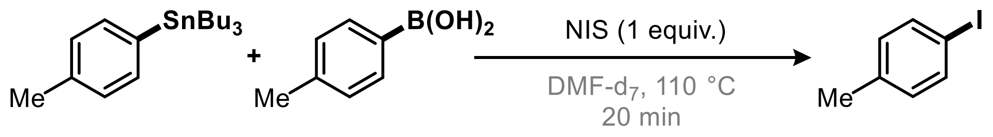

To test the intermolecular tolerance of the $SnBu_3$- and $B(OH)_2$-site towards an established iodination approach, tributyl(*p*-tolyl)stannane (19.1 mg, 0.05 mmol, 1 equiv.), *p*-tolylboronic acid (6.8 mg, 0.05 mmol, 1 equiv.) and NIS (11.2 mg, 0.05 mmol, 1 equiv.) were dissolved in DMF-d_7 (0.3 mL) and stirred for 20 min at 110 °C. The reaction mixture was analyzed by quantitative ^{1}H NMR (using 1,4-difluorobenzene as internal standard). The analysis revealed full consumption of aryl stannane and 38% consumption of aryl boronic acid.

Tolerance of Functional Handles in Fluorination Approaches

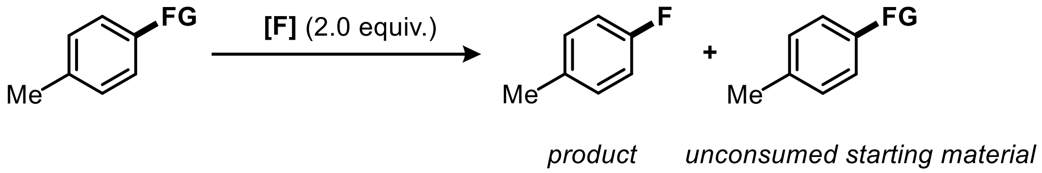

To test the tolerance of the $GeEt_3$-, $SnBu_3$-, $B(OH)_2$-, B(pin)- and $SiMe_3$-site towards established fluorination approaches, the corresponding starting materials (0.03 mmol, 1 equiv.) and the corresponding reagents (for details see Table S1) were dissolved in the DMF-d_7 and stirred under reaction conditions as specified. The procedures were adopted from literature.[16] The reaction mixtures were analyzed by calibrated GC-MS and quantitative ^1H NMR (using mesitylene as internal standard). The results are shown in Table S1.

Table S1: Consumption of functional handles in established fluorination approaches.

	**$GeEt_3$**	**$SnBu_3$**	**$B(OH)_2$**	**B(pin)**	**$SiMe_3$**
KF (2 equiv.)<br>18-crown-6 (2 equiv.)<br>DMF-d_7; 110 °C, 1 h	0%	100%	100%	20%	8%
KF (2 equiv.)<br>18-crown-6 (2 equiv.)<br>[$Cu(OTf)_2$] (2 equiv.)<br>DMF-d_7; 110 °C, 1 h	1%	100%	100%	55%	12%
Selectfluor (2 equiv.)<br>DMF-d_7, 80 °C, 1 h	2%	100%	100%	63%	22%
Selectfluor (2 equiv.)<br>$AgBF_4$ (2 equiv.)<br>DMF-d_7, 80 °C, 1 h	0%	100%	100%	82%	0%

Consumption determined by calibrated GC-MS analysis or by quantitative ^1H NMR analysis using mesitylene as internal standard.

Selective Fluorination of $SnBu_3$ *vs.* $GeEt_3$

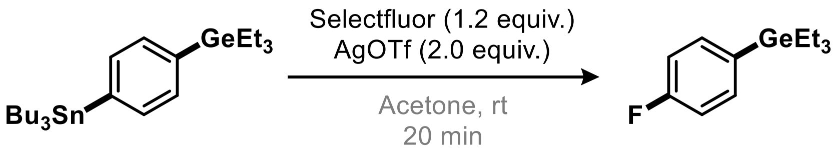

Triethyl(4-(tributylstannyl)phenyl)germane (26.3 mg, 0.05 mmol, 1 equiv.), Selectfluor (17.7 mg, 0.05 mmol, 1 equiv.) and AgOTf (25.7 mg, 0.10 mmol, 2 equiv.) were dissolved in acetone (1 mL) and stirred for 20 min at rt. The solvent was removed *in vacuo* and the residue dissolved in hexane. The suspension was filtered over a plug of silica and concentrated *in vacuo*. The mixture was subsequently analyzed by quantitative ^{1}H and ^{19}F NMR (using 1,4-difluorobenzene as internal standard). The analysis revealed full consumption of the starting material and 96% conversion to the desired fluorinated product. The analysis data matches the one previously reported in this manuscript (see S6).

Intermolecular Competition in Iodination

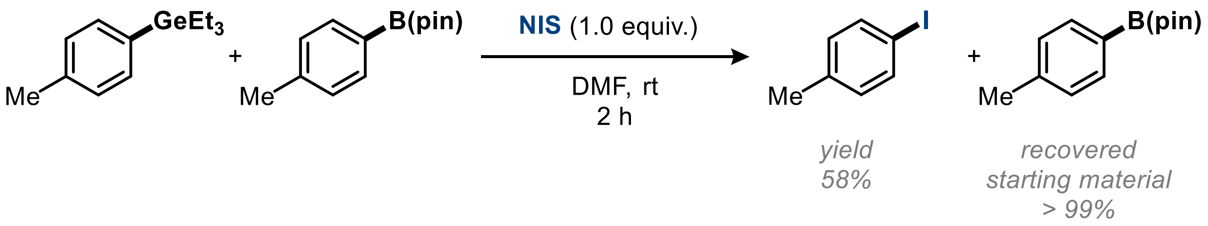

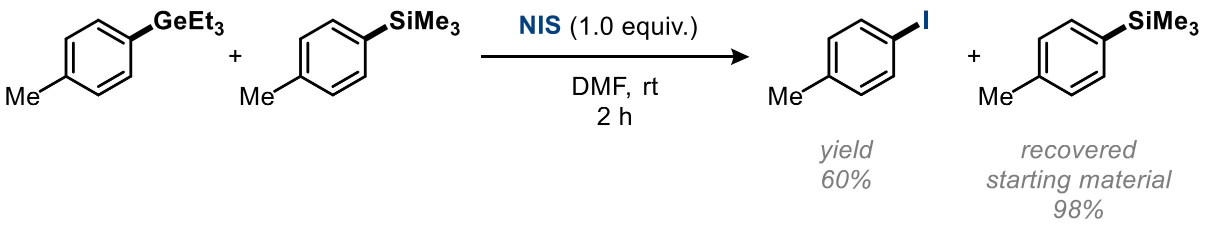

Aryl germane (25.1 mg, 0.1 mmol, 1.0 equiv.) and Ar-FG (0.1 mmol, 1.0 equiv.; FG = B(pin); FG = $SiMe_3$) were dissolved in DMF (1 mL) and NIS (17.8 mg, 0.1 mmol, 1.0 equiv.) was added in one portion. The reaction was stirred at room temperature and quenched after 2 h by dilution with EtOAc and subsequently analyzed by calibrated GC-MS (using mesitylene as internal standard) regarding consumption of starting material and formation of desired halogenated product.

Individual Bromination and Iodination

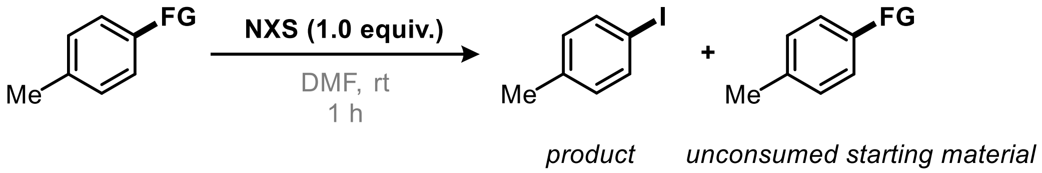

Ar-FG (0.1 mmol, 1.0 equiv.; **(1)** FG = $GeEt_3$; **(2)** FG = B(pin); **(3)** FG = $SiMe_3$) was dissolved in DMF (1 mL) and NXS (NBS or NIS; 0.1 mmol, 1.0 equiv.) was added in one portion. The reaction was stirred at room temperature and quenched after 1 h by dilution with EtOAc and subsequently analyzed by calibrated GC-MS (using mesitylene as internal standard) regarding consumption of starting material and formation of desired halogenated product. The results are shown in Table S2 and S3.

Table S2: Bromination of different functional handles.

entry	FG	product [%][a]	recovered ArFG [%][a]
1	$GeEt_3$	96	0
2	B(pin)	0	> 99
3	$SiMe_3$	0	> 99

[a]Determined by calibrated GC-MS analysis using mesitylene as internal standard.

Table S3: Iodination of different functional handles.

entry	FG	product [%][a]	recovered ArFG [%][a]
1	$GeEt_3$	62	30
2	B(pin)	0	> 99
3	$SiMe_3$	0	> 99

[a]Determined by calibrated GC-MS analysis using mesitylene as internal standard.

Intramolecular Competition

2-(4-Bromophenyl)-4,4,5,5-tetramethyl-1,3,2-dioxaborolane

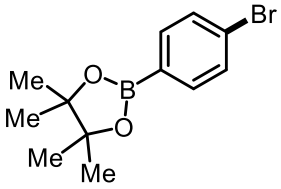

Prepared according to GP 1 The title product was obtained after purification by column chromatography (pentane/Et_2O, 7:1) as a colorless oil (41.3 mg, 0.282 mmol, 94%).

R$_f$ = 0.73 (pentane/Et_2O, 9:1). **^{1}H NMR** (600 MHz, $CDCl_3$) δ/ ppm = 7.66 (d, *J* = 8.3 Hz, 2H), 7.50 (d, *J* = 8.3 Hz, 2H), 1.34 (s, 12H). **^{13}C NMR** (151 MHz, $CDCl_3$) δ/ ppm = 136.4, 131.1, 126.4, 84.2, 25.0. $C_{12}H_{16}O_2B^{79}Br$: 282.0421 [M]$^+$, found: 282.0422.

1 C atom missing in the ^{13}C NMR. This is in line with literature reports.

These data are in agreement with those reported previously in the literature.[17]

(4-Bromophenyl)trimethylsilane

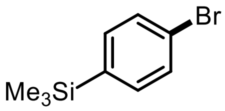

Prepared according to GP 1. The title product was obtained after purification by column chromatography (pentane) as a colorless oil (66.8 mg, 0.288 mmol, 96%).

R$_f$ = 0.84 (pentane). **^{1}H NMR** (600 MHz, $CDCl_3$) δ/ ppm = 7.49 (d, *J* = 8.2 Hz, 2H), 7.38 (d, *J* = 8.2 Hz, 2H), 0.26 (d, *J* = 0.8 Hz, 9H). **^{13}C NMR** (151 MHz, $CDCl_3$) δ/ ppm = 139.4, 135.1, 131.0, 123.7, -1.1. **MS** (70 eV, EI): *m/z* (%): 230.0 (12) [M]$^+$ (^{81}Br), 228.0 (11) [M]$^+$ (^{79}Br), 215.0 (100), 213.0 (99), 119.0 (11), 106.9 (10), 91.1 (20).

These data are in agreement with those reported previously in the literature.[18]

2-(4-Iodophenyl)-4,4,5,5-tetramethyl-1,3,2-dioxaborolane

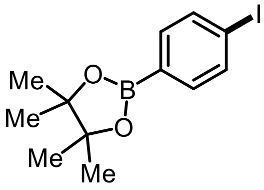

Prepared according to GP 2. The title product was obtained after purification by column chromatography (pentane/Et_2O, 7:1) as a yellow solid (38.7 mg, 0.231 mmol, 77%).

R$_f$ = 0.72 (pentane/Et_2O, 9:1). **^{1}H NMR** (600 MHz, $CDCl_3$) δ/ppm = 7.72 (d, *J* = 8.1 Hz, 2H), 7.51 (d, *J* = 8.1 Hz, 2H), 1.33 (s, 12H). **^{13}C NMR** (151 MHz, $CDCl_3$) δ/ppm = 137.1, 136.4, 99.0, 84.2, 29.8, 25.0. **HRMS** (EI) calculated for $C_{11}H_{13}O_2B$: 315.0048 [M-Me]$^+$, found: 315.0051.

These data are in agreement with those reported previously in the literature.[19]

(4-Iodophenyl)trimethylsilane

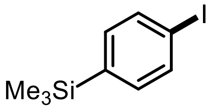

Prepared according to GP 2. The title product was obtained after purification by column chromatography (pentane) as a colorless oil (40.6 mg, 0.279 mmol, 93%).

R_f = 0.80 (pentane). **^{1}H NMR** (600 MHz, $CDCl_3$) δ/ ppm = 7.70 (d, *J* = 7.6 Hz, 2H), 7.25 (d, *J* = 7.6 Hz, 2H), 0.26 (d, *J* = 1.1 Hz, 9H). **^{13}C NMR** (151 MHz, $CDCl_3$) δ/ ppm = 140.0, 136.9, 135.2, 95.8, -1.1. **HRMS** (EI) calculated for $C_9H_{13}ISi$: 275.9826 $[M]^+$, found: 275.9822. These data are in agreement with those reported previously in the literature.[20]

Mechanistic Investigation

Linear Free Energy Relationship Analysis (*Hammett Plot*)

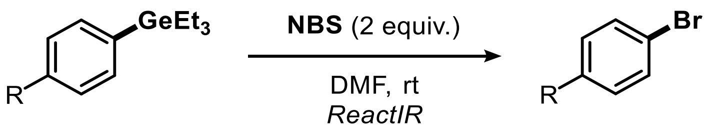

The reaction was performed according to GP 1. It was monitored using a Mettler Toledo *ReactIR*® *15* equipped with a 6.3 mm probe. The relative absorption data over time was normalized to yields obtained by calibrated GC-MS (using mesitylene as internal standard) or quantitative ^{1}H or ^{19}F NMR analysis (using mesitylene or 1,4-difluorobenzene as internal standard).

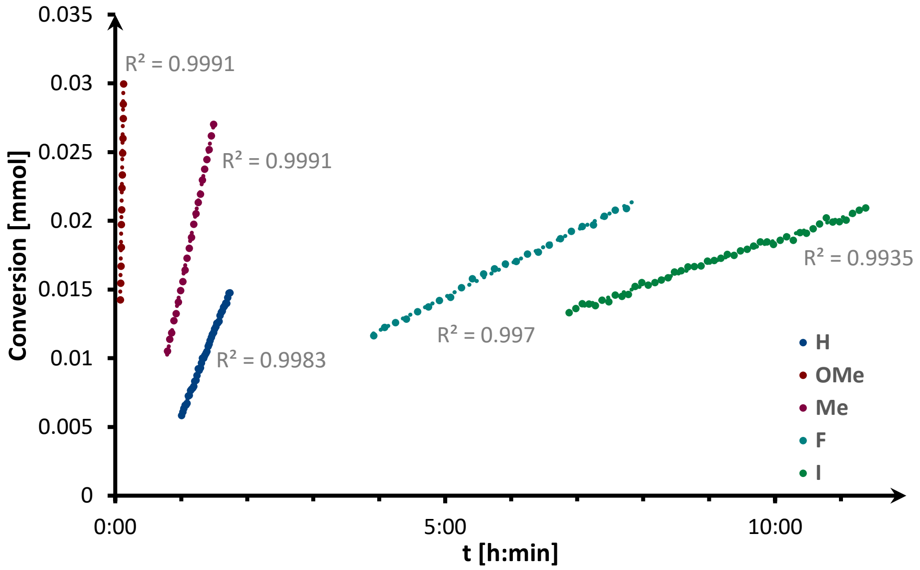

Figure S1: Determination of initial reaction rates.

Table S4: Reaction rates for different substitution patterns.

entry	$R^{ArGeEt3}$	k [mol s^{-1}]	$\log(k/k_H)$	σ_p [21]	σ_p^+ [21]
1	**4-OMe**	7.576	1.41	-0.268	-0.778
2	**4-Me**	0.568	0.29	-0.17	-0.311
3	**4-H**	0.294	0.00	0	0
4	**4-F**	0.058	-0.70	0.062	-0.073
5	**4-I**	0.039	-0.87	0.18	0.135

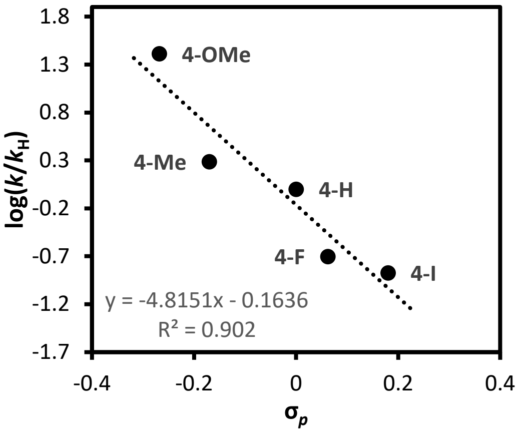

Figure S2: Hammett Plot σ_p.

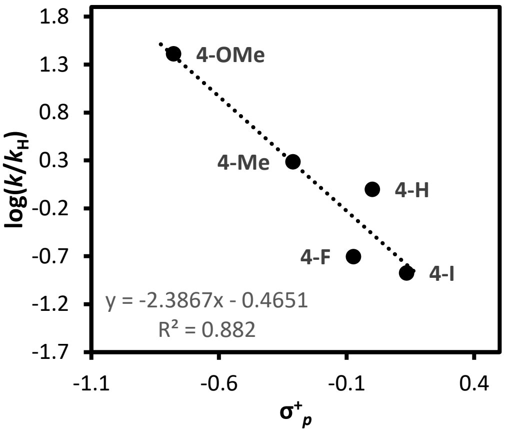

Figure S3: Hammett Plot σ_p^+.

Robustness Screen

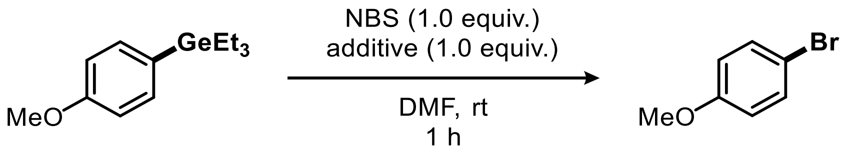

The reaction was performed according to GP 1. In addition, an additive (1.0 equiv., for details see Table S5) was added to the reaction mixture. The tolerance of the reaction towards each additive was shown by quantification of yield of product (**Y**), remaining additive (**A**) and remaining starting material (**SM**) using calibrated GC-MS with mesitylene as internal standard. The results are shown in Table S5 (green: >66%; yellow 34-66%; red <34%).

Table S5: Robustness screen for aryl germanes.

entry	additive	Y [%]	A [%]	SM [%]	entry	additive	Y [%]	A [%]	SM [%]
**1**	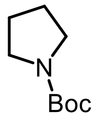	86	83	0	**10**	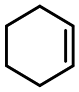	85	n.d.	0
**2**	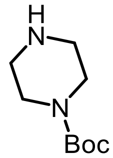	51	n.d.	33	**11**	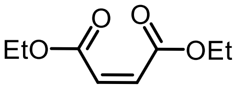	89	81	0
**3**	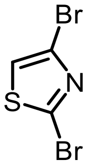	96	84	0	**12**	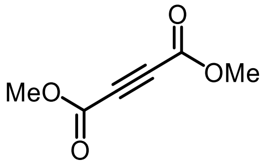	95	74	0
**4**	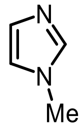	57	n.d.	30	**13**	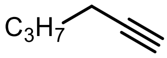	85	n.d.	0
**5**	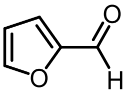	83	99	0	**14**	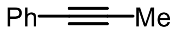	79	99	0
**6**	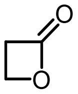	84	n.d.	0	**15**	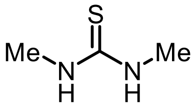	15	0	36
**7**	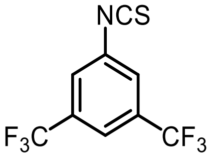	87	n.d.	0	**16**	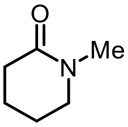	98	99	0
**8**	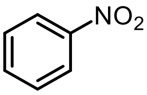	88	48	0	**17**	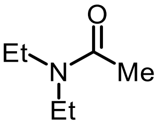	86	48	0
**9**	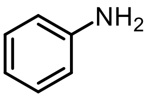	0	n.d.	68	**18**	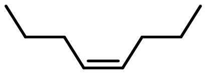	81	n.d.	0

entry	additive	Y [%]	A [%]	SM [%]	entry	additive	Y [%]	A [%]	SM [%]
**19**	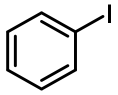	84	99	0	**32**	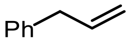	84	95	0
**20**	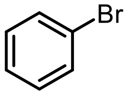	87	99	0	**33**	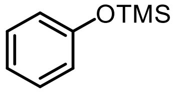	92	51	0
**21**	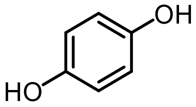	63	0	26	**34**	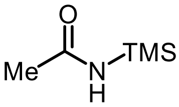	99	n.d.	0
**22**	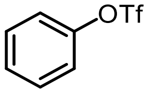	80	99	0	**35**	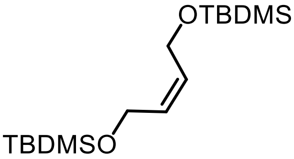	94	48	0
**23**	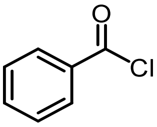	97	29	0	**36**	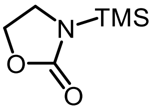	89	37	0
**24**	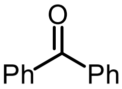	83	99	0	**37**	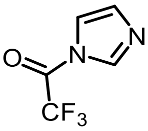	99	n.d.	0
**25**	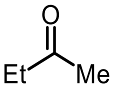	86	n.d.	0	**38**	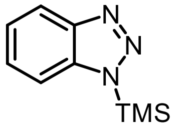	92	21	0
**26**	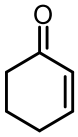	87	99	0	**39**	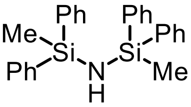	99	n.d.	0
**27**	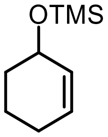	22	0	57	**40**	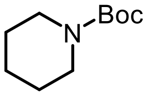	99	90	0
**28**	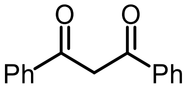	18	0	61	**41**	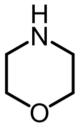	68	0	21
29	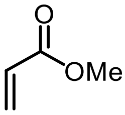	83	n.d.	0	**42**	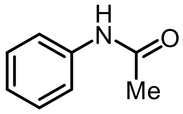	99	99	0
30	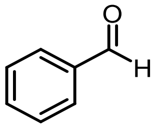	58	n.d.	0	**43**	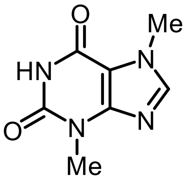	83	n.d.	0
31	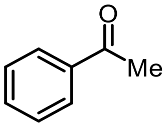	87	99	0	**44**	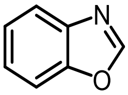	93	99	0

entry	additive	Y [%]	A [%]	SM [%]	entry	additive	Y [%]	A [%]	SM [%]
**45**	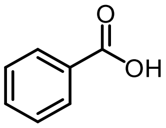	89	n.d.	0	**49**	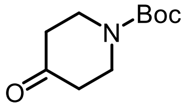	99	99	0
**46**	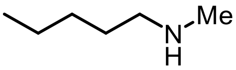	85	n.d.	0	**50**	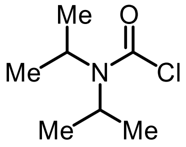	95	0	0
**47**	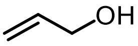	83	n.d.	0	**51**	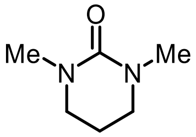	88	41	0
**48**	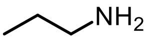	83	n.d.	0	**52**	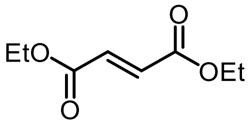	83	91	0

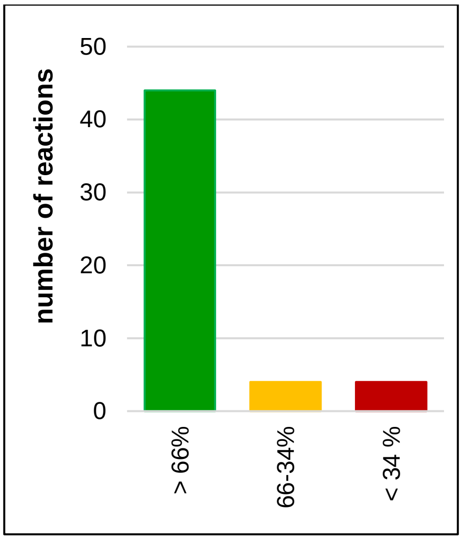

Figure S4: Yield of bromination.

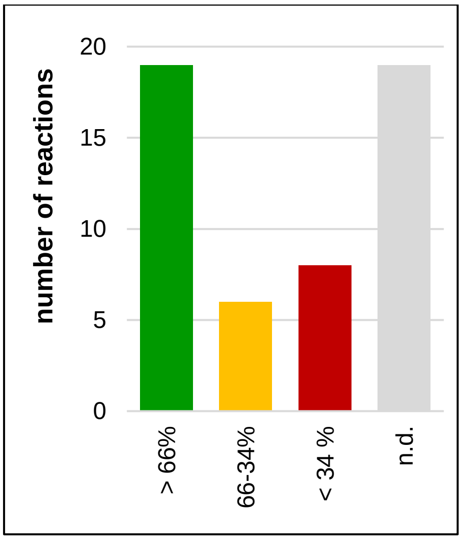

Figure S5: Recovery of additive.

The vast majority of the additives is well tolerated. Due to their low molecular weight and resulting low boiling point, it was impossible to quantify some of the additives via GC-MS (marked in grey). However, also in those cases the reaction proceeded with high efficiency.

Robustness Screen for ArB(pin) and $ArSiMe_3$

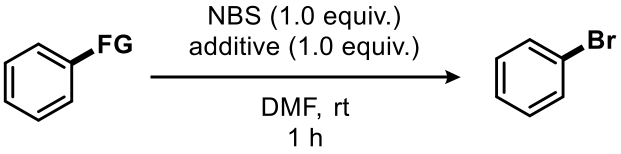

The reaction was performed according to GP 1 with ArFG (FG = $GeEt_3$, B(pin) or $SiMe_3$; 0.03 mmol, 1 equiv.). To investigate the tolerance of the reaction, different additives were added to the reaction mixture (see table below for details). The reaction was stirred for 1 h at rt and subsequently analyzed by calibrated GC-MS using mesitylene (1 equiv.) as internal standard. The results are shown in Table S6.

Table S6: Robustness screen comparing aryl germanes with aryl boronic esters and aryl silanes.

entry	FG	additive	recovery of additive [%][a]	yield [%][a]
1	$GeEt_3$	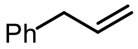	96	84
2	$SiMe_3$	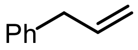	0	0
3	Bpin	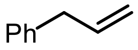	0	0
4	$GeEt_3$	Me—≡—Ph	> 99	79
5	$SiMe_3$	Me—≡—Ph	39[b]	0
6	Bpin	Me—≡—Ph	41[b]	0

[a]Determined by calibrated GC-MS analysis using mesitylene as internal standard. [b]Brominated side products formed.

EPR Analysis

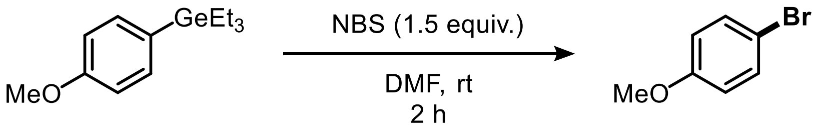

The reaction was performed according to GP 1. EPR measurements of the reaction mixture were performed every 20 min aiming to detect paramagnetic species which would indicate a SET type mechanism. No paramagnetic species were detected.

Additionally, the reaction was performed according to GP 1 with either 2,2,6,6-tetramethylpiperidinyloxyl (TEMPO) or N-*tert*-butyl-α-phenylnitrone (PBN) as spin probe/spin trap. After 2 h of reaction time, the reaction mixture was analyzed by EPR.

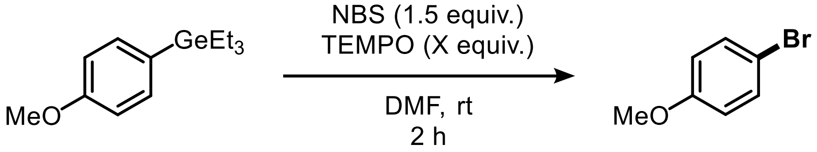

Table S7: TEMPO as spin probe.

entry	equiv. TEMPO	yield [%][a]
1	0.5	99
2	1.0	99
3	2.0	99

[a]Determined by calibrated GC-MS analysis using mesitylene as internal standard.

In all experiments with various amounts of TEMPO as spin probe no TEMPO-related adducts were detected (GC-MS). Additionally, EPR measurements did not show any change in the TEMPO signal.

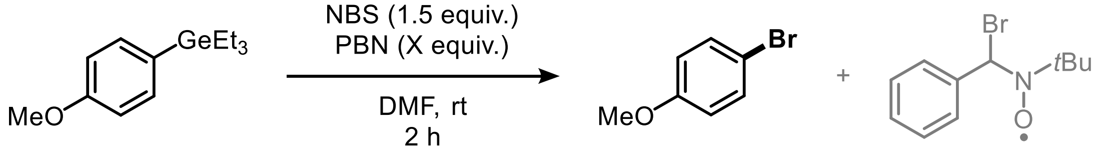

Table S8: PBN as spin trap.

entry	equiv. PBN	yield [%][a]
1	0.5	99
2	1.0	99
3	2.0	99

[a]Determined by calibrated GC-MS analysis using mesitylene as internal standard.

In all experiments with various amounts of PBN as spin trap no (paramagnetic) PBN-related adducts of the aryl germane were detected (GC-MS and EPR analysis). Due to the excess of NBS, some paramagnetic N-O species were formed (EPR), which indicate reaction of an Br-radical with PBN (unambiguously confirmed by GC-MS analysis). This observation is in line with literature reports. [22] The quantitative yield confirmed that the aryl germane did not form significant amount of aryl radicals which might be trapped by the spin trap.

Reaction Monitoring

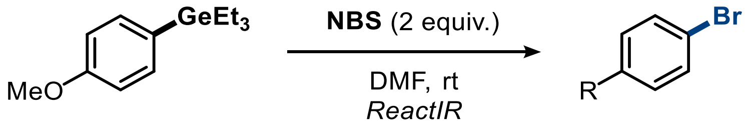

The reaction was performed according to GP 1. It was monitored using a Mettler Toledo *ReactIR*® *15* equipped with a 6.3 mm probe. The relative absorption data over time was normalized to yields obtained by calibrated GC-MS (using mesitylene as internal standard).

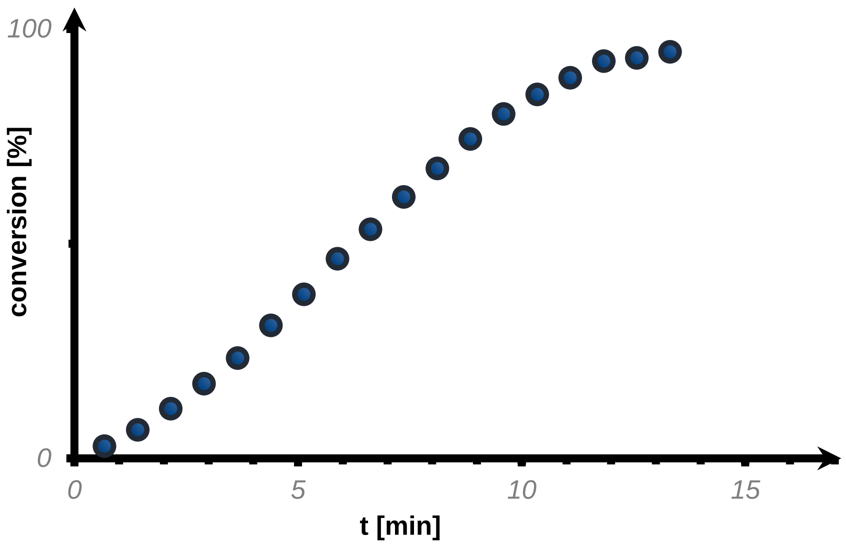

Figure S6: Reaction monitoring (product formation) of bromination with NBS.

Computational Details

DFT calculations were performed using the Gaussian software package (Gaussian16, revision A.03).[23] Geometry optimization and frequency calculations were conducted with implicit solvation (CPCM model for DMF) at the ωB97XD/def2SVP level of theory. Calculated frequencies were used to verify the nature of all stationary points as either minima (no imaginary frequencies) or transition states (one imaginary frequency). Intrinsic reaction coordinate (IRC) calculations were performed to further confirm whether the transition state is connected to the corresponding intermediates. Single point energies were calculated at the M06/6-311++G(d,p) level of theory employing SDD as an ECP for Br, I and Ge and the CPCM solvation model for DMF. Energies of all structures were corrected to 1 M standard state (addition of 1.89 kcal/mol to every species). Images were created using the *CYLview* software.[24]

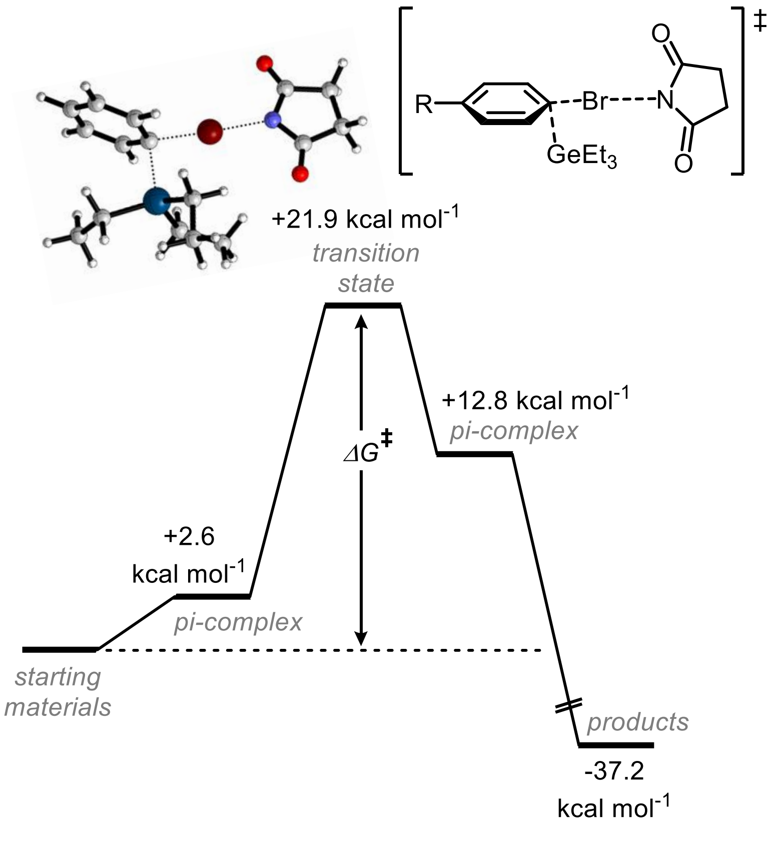

Figure S7: Gibbs free energies computed at the CPCM (DMF) M06/6-311++G(d,p) (SDD)//ωB97XD/def2SVP level of theory. Energies are given relative to starting materials.

Influence of *para*-Substituents on the Transition State Barrier (DFT)

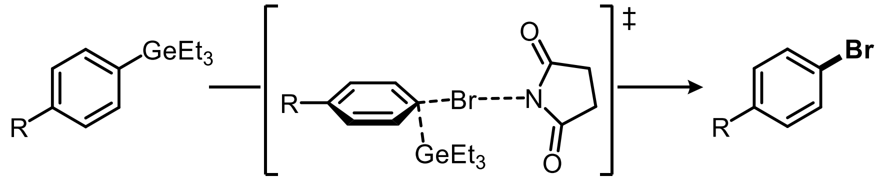

Table S9: Transition state barriers.

entry	R	ΔG‡ [kcal/mol]	σ_p [21]
1	OMe	17.8	-0.268
2	Me	19.6	-0.170
3	H	21.9	0.000
4	F	22.2	0.062
5	I	23.8	0.180

Gibbs free energies computed at the CPCM (DMF) M06/6-311++G(d,p) (SDD)//ωB97XD/def2SVP level of theory. Energy barriers of transition states are relative to starting materials.

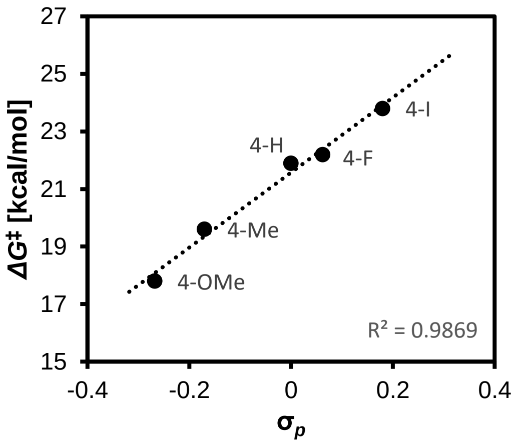

Figure S8: Transition state barriers for various substitution patterns plotted against σ_p value.

Comparison of Aryl Germanes with Aryl Silanes

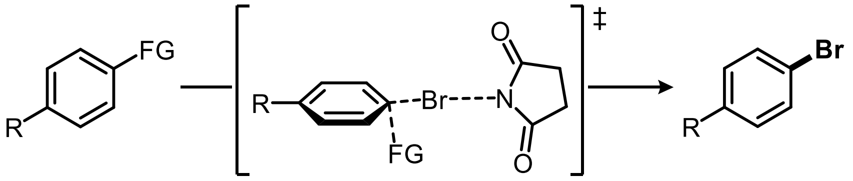

Table S10: Transition state barriers.

entry	FG	*ΔG*‡ [kcal mol-1]
1	$GeEt_3$	21.9
2	$SiMe_3$	25.2

Gibbs free energies computed at the CPCM (DMF) M06/6-311++G(d,p) (SDD)//ωB97XD/def2SVP level of theory. Energy barriers of transition states are relative to starting materials.

XYZ Coordinates and Energies

PhGeEt$_3$

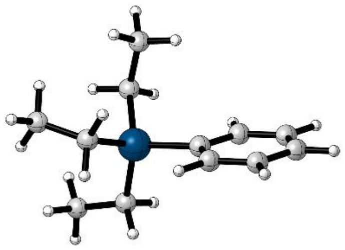

Ge	-0.695547000	-0.099708000	-0.077195000
C	2.049985000	0.226690000	-1.065931000
C	3.443872000	0.184369000	-1.007452000
C	1.263422000	-0.140212000	0.037513000
C	4.080679000	-0.229275000	0.163598000
C	1.922788000	-0.551633000	1.205680000
C	3.316973000	-0.597937000	1.271429000
H	4.035794000	0.476339000	-1.878364000
H	5.171617000	-0.262794000	0.212867000
H	1.343747000	-0.842365000	2.087427000
C	-1.432056000	0.001567000	1.744207000
H	-1.337329000	-0.994393000	2.208665000
H	-0.781505000	0.673863000	2.328426000
C	-1.227165000	1.489728000	-1.101746000
H	-0.854307000	1.373763000	-2.133370000
H	-2.327421000	1.490944000	-1.176992000
C	-1.277525000	-1.725624000	-1.015558000
H	-0.785306000	-1.716140000	-2.003012000
H	-0.858365000	-2.590466000	-0.474355000
H	3.808986000	-0.921709000	2.191876000
H	1.570922000	0.556564000	-1.993248000
C	-0.729598000	2.802307000	-0.495342000
H	0.370204000	2.824331000	-0.435637000
H	-1.045423000	3.674787000	-1.089027000
H	-1.112910000	2.946490000	0.527692000
C	-2.790612000	-1.878861000	-1.175994000
H	-3.237142000	-1.012517000	-1.689565000
H	-3.048214000	-2.774835000	-1.762945000
H	-3.292563000	-1.974981000	-0.200661000
C	-2.881609000	0.487147000	1.814595000
H	-2.990720000	1.495958000	1.386309000
H	-3.564358000	-0.174551000	1.260021000
H	-3.243117000	0.532783000	2.854125000

Zero-point correction = 0.288402 (Hartree/Particle)

Thermal correction to Energy = 0.305101

Thermal correction to Enthalpy = 0.306046

Thermal correction to Gibbs Free Energy = 0.242248

Sum of electronic and zero-point Energies = -2545.390337

Sum of electronic and thermal Energies = -2545.373637

Sum of electronic and thermal Enthalpies = -2545.372693

Sum of electronic and thermal Free Energies = -2545.436490

E(RM06) = -472.859048239

4-Me-PhGeEt$_3$

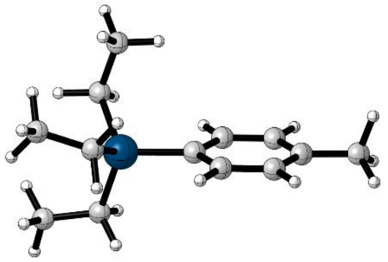

Ge	-1.048105000	-0.101351000	-0.077091000
C	1.678506000	0.280669000	-1.101727000
C	3.072296000	0.258490000	-1.063707000
C	0.910461000	-0.109939000	0.005741000
C	3.756688000	-0.155003000	0.086608000
C	1.600735000	-0.524558000	1.153620000
C	2.995452000	-0.548892000	1.193645000
H	3.641303000	0.567825000	-1.945284000
H	1.046101000	-0.839588000	2.042858000
C	-1.757849000	-0.035292000	1.756923000
H	-1.642140000	-1.036214000	2.205757000
H	-1.107227000	0.637627000	2.340368000
C	-1.624448000	1.491369000	-1.072626000
H	-1.267060000	1.394233000	-2.111695000
H	-2.725710000	1.475390000	-1.129569000
C	-1.623887000	-1.723722000	-1.026268000
H	-1.150457000	-1.693834000	-2.022507000
H	-1.181450000	-2.589090000	-0.504722000
H	3.503045000	-0.881267000	2.103847000
H	1.184834000	0.612112000	-2.020936000
C	-1.138275000	2.804585000	-0.458437000
H	-0.038131000	2.843689000	-0.416488000
H	-1.478004000	3.679110000	-1.035762000
H	-1.506669000	2.929914000	0.572567000
C	-3.137129000	-1.898605000	-1.160731000
H	-3.606540000	-1.032642000	-1.654196000
H	-3.392236000	-2.790701000	-1.754712000
H	-3.619135000	-2.015225000	-0.177586000
C	-3.212543000	0.429099000	1.856990000
H	-3.341941000	1.442320000	1.445071000
H	-3.894793000	-0.233838000	1.303263000

H	-3.558597000	0.454886000	2.902482000
C	5.262354000	-0.146989000	0.139442000
H	5.700202000	-0.347590000	-0.848916000
H	5.635759000	0.836066000	0.469695000
H	5.644024000	-0.897198000	0.846548000

Zero-point correction = 0.315646 (Hartree/Particle)
Thermal correction to Energy = 0.334253
Thermal correction to Enthalpy = 0.335197
Thermal correction to Gibbs Free Energy = 0.266047
Sum of electronic and zero-point Energies = -2584.642123
Sum of electronic and thermal Energies = -2584.623516
Sum of electronic and thermal Enthalpies = -2584.622572
Sum of electronic and thermal Free Energies = -2584.691722
E(RM06) = -512.153686994

4-F-PhGeEt$_3$

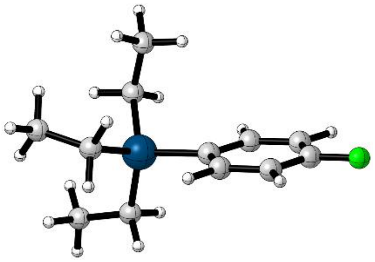

Ge	-1.025136000	-0.108393000	-0.065285000
C	1.700021000	0.137035000	-1.129128000
C	3.094386000	0.125023000	-1.099602000
C	0.935595000	-0.108156000	0.022451000
C	3.730448000	-0.138586000	0.107282000
C	1.622161000	-0.367850000	1.217954000
C	3.016606000	-0.386634000	1.272478000
H	3.689218000	0.317553000	-1.994265000
H	1.066578000	-0.561460000	2.139897000
C	-1.734905000	0.141533000	1.751766000
H	-1.613653000	-0.806719000	2.301885000
H	-1.089787000	0.876003000	2.262552000
C	-1.591047000	1.380545000	-1.214207000
H	-1.230608000	1.181673000	-2.237540000
H	-2.692096000	1.362540000	-1.273268000
C	-1.589956000	-1.818150000	-0.851534000
H	-1.099381000	-1.892363000	-1.837026000
H	-1.159949000	-2.625505000	-0.235285000
H	3.551449000	-0.588559000	2.202271000
H	1.205160000	0.347428000	-2.082021000
C	-1.102512000	2.745480000	-0.727950000
H	-0.002425000	2.785375000	-0.684734000
H	-1.436545000	3.560496000	-1.389380000
H	-1.475279000	2.971549000	0.284102000
C	-3.101410000	-2.002362000	-0.993919000
H	-3.557974000	-1.192696000	-1.585186000
H	-3.349117000	-2.951913000	-1.494474000
H	-3.601497000	-2.012952000	-0.013007000
C	-3.192607000	0.604927000	1.802083000
H	-3.327864000	1.568893000	1.286769000
H	-3.870512000	-0.116515000	1.320924000
H	-3.539080000	0.737554000	2.839214000
F	5.068562000	-0.152070000	0.147961000

Zero-point correction = 0.280169 (Hartree/Particle)
Thermal correction to Energy = 0.297699
Thermal correction to Enthalpy = 0.298643
Thermal correction to Gibbs Free Energy = 0.232655
Sum of electronic and zero-point Energies = -2644.532454
Sum of electronic and thermal Energies = -2644.514925
Sum of electronic and thermal Enthalpies = -2644.513981
Sum of electronic and thermal Free Energies = -2644.579969
E(RM06) = -572.101663604

4-I-PhGeEt$_3$

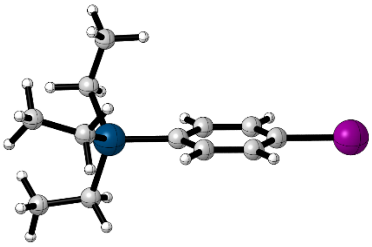

Ge	-2.532602000	-0.108280000	-0.068058000
C	0.169768000	0.248565000	-1.163335000
C	1.565016000	0.260956000	-1.158522000
C	-0.568345000	-0.077787000	-0.016362000
C	2.243546000	-0.060365000	0.017572000
C	0.144798000	-0.393979000	1.148044000
C	1.540682000	-0.389572000	1.176291000
H	2.111775000	0.520962000	-2.066827000
H	-0.388007000	-0.651927000	2.067787000
C	-3.208547000	0.035209000	1.772629000
H	-3.061083000	-0.938161000	2.269886000
H	-2.565466000	0.753317000	2.308547000
C	-3.140384000	1.427383000	-1.129710000
H	-2.796025000	1.285430000	-2.167920000
H	-4.241895000	1.394219000	-1.170089000
C	-3.077381000	-1.786549000	-0.931079000
H	-2.611428000	-1.796192000	-1.931101000
H	-2.613851000	-2.615288000	-0.370143000
H	2.068215000	-0.640493000	2.098433000

H	-0.344474000	0.505690000	-2.094355000
C	-2.663805000	2.774034000	-0.584114000
H	-1.563863000	2.830388000	-0.559672000
H	-3.023546000	3.615234000	-1.197442000
H	-3.020307000	2.942933000	0.444764000
C	-4.587851000	-1.997223000	-1.044701000
H	-5.077335000	-1.167598000	-1.579212000
H	-4.827859000	-2.923863000	-1.589820000
H	-5.061161000	-2.072299000	-0.053378000
C	-4.672365000	0.470172000	1.873528000
H	-4.832916000	1.457098000	1.411848000
H	-5.347296000	-0.236970000	1.367657000
H	-5.000862000	0.542628000	2.922329000
I	4.354148000	-0.045381000	0.043493000

Zero-point correction = 0.277783 (Hartree/Particle)
Thermal correction to Energy = 0.296141
Thermal correction to Enthalpy = 0.297085
Thermal correction to Gibbs Free Energy = 0.227418
Sum of electronic and zero-point Energies = -2842.574895
Sum of electronic and thermal Energies = -2842.556537
Sum of electronic and thermal Enthalpies = -2842.555593
Sum of electronic and thermal Free Energies = -2842.625260
E(RM06) = -483.648464614

4-OMe-PhGeEt$_3$

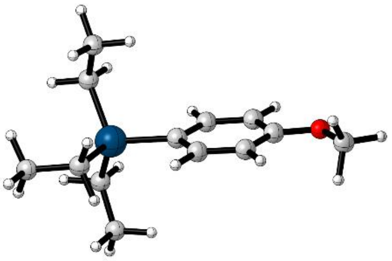

C	0.478145000	-0.000014000	-0.142347000
C	1.214146000	-0.000083000	1.046958000
C	1.206871000	0.000047000	-1.346318000
C	2.612966000	-0.000093000	1.058313000
C	2.595245000	0.000040000	-1.360050000
C	3.312645000	-0.000030000	-0.153179000
H	0.696426000	-0.000133000	2.010639000
H	0.679933000	0.000103000	-2.306076000
H	3.138399000	-0.000150000	2.013650000
H	3.153239000	0.000089000	-2.298803000
Ge	-1.478969000	0.000014000	-0.165129000
C	-2.103399000	-1.606305000	-1.107456000
H	-3.199309000	-1.538270000	-1.211749000
C	-2.103322000	1.606430000	-1.107342000
H	-1.696207000	1.574281000	-2.132075000
C	-2.142710000	-0.000036000	1.682652000
H	-1.728394000	0.881383000	2.200183000
H	-1.728411000	-0.881492000	2.200133000
H	-3.199246000	1.538500000	-1.211566000
H	-1.696212000	-1.574145000	-2.132160000
O	4.658029000	-0.000036000	-0.257888000
C	5.434646000	-0.000037000	0.915815000
H	5.246105000	0.897437000	1.528477000
H	5.246173000	-0.897558000	1.528432000
H	6.483914000	0.000010000	0.598216000
C	-1.708812000	-2.910681000	-0.414665000
H	-0.615547000	-2.990813000	-0.301824000
H	-2.145667000	-2.979377000	0.594528000
H	-2.047183000	-3.794664000	-0.978274000
C	-3.669779000	-0.000023000	1.768988000
H	-4.021174000	-0.000050000	2.812936000
H	-4.102464000	0.886548000	1.278309000
H	-4.102482000	-0.886558000	1.278258000
C	-1.708571000	2.910738000	-0.414518000
H	-2.046901000	3.794779000	-0.978062000
H	-2.145351000	2.979426000	0.594709000
H	-0.615291000	2.990766000	-0.301748000

Zero-point correction = 0.321058 (Hartree/Particle)
Thermal correction to Energy = 0.340378
Thermal correction to Enthalpy = 0.341322
Thermal correction to Gibbs Free Energy = 0.271082
Sum of electronic and zero-point Energies = -2659.765698
Sum of electronic and thermal Energies = -2659.746378
Sum of electronic and thermal Enthalpies = -2659.745434
Sum of electronic and thermal Free Energies = -2659.815675
E(RM06) = -587.356480426

NBS

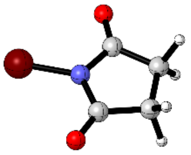

C	-2.360631000	0.764556000	-0.000257000
C	-0.905152000	1.180406000	-0.000021000
C	-2.360629000	-0.764563000	0.000473000
N	-0.154834000	0.000001000	0.000006000
C	-0.905148000	-1.180408000	0.000114000
H	-2.842827000	1.201616000	-0.885617000
H	-2.842746000	-1.201622000	0.885878000
H	-2.843677000	-1.202611000	-0.883923000
H	-2.843598000	1.202603000	0.884182000

Br	1.676789000	0.000001000	-0.000079000
O	-0.440103000	2.287388000	0.000142000
O	-0.440093000	-2.287387000	-0.000099000

Zero-point correction = 0.082115 (Hartree/Particle)
Thermal correction to Energy = 0.089457
Thermal correction to Enthalpy = 0.090401
Thermal correction to Gibbs Free Energy = 0.048021
Sum of electronic and zero-point Energies = -2933.483240
Sum of electronic and thermal Energies = -2933.475899
Sum of electronic and thermal Enthalpies = -2933.474954
Sum of electronic and thermal Free Energies = -2933.517335
E(RM06) = -373.274034339

pi_NBS_PhGeEt$_3$

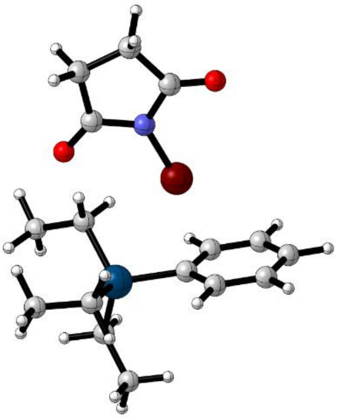

Ge	-2.106828000	-0.674874000	0.071231000
C	-1.735970000	1.255344000	0.111533000
C	-1.368731000	1.965458000	-1.043553000
C	-1.837822000	1.971019000	1.315572000
C	-1.100481000	3.336887000	-0.996080000
C	-1.576088000	3.340090000	1.368805000
C	-1.202673000	4.026100000	0.211557000
H	-1.286722000	1.445543000	-2.002646000
H	-2.122877000	1.453110000	2.236838000
H	-0.811152000	3.866554000	-1.906769000
H	-1.659572000	3.874733000	2.318040000
H	-0.991930000	5.097182000	0.252042000
C	-4.041366000	-0.916059000	0.322727000
H	-4.254120000	-1.997814000	0.345486000
H	-4.297876000	-0.532631000	1.324916000
C	-1.562557000	-1.381424000	-1.678385000
H	-0.524904000	-1.059459000	-1.865841000
H	-2.181255000	-0.900125000	-2.454354000
C	-1.120109000	-1.553462000	1.527500000
H	-0.094977000	-1.730639000	1.163426000
H	-1.031935000	-0.833943000	2.358541000
Br	1.539347000	0.812937000	-0.229840000
C	4.598641000	-1.908228000	0.158237000
C	3.143081000	-1.513008000	0.029417000
N	3.110736000	-0.119837000	-0.047918000
C	4.360427000	0.503984000	0.012624000
C	5.388080000	-0.598279000	0.158114000
H	4.717725000	-2.492167000	1.081315000
O	2.176171000	-2.227206000	-0.003559000
O	4.535685000	1.690710000	-0.042923000
H	6.102467000	-0.517014000	-0.672716000
H	4.851176000	-2.570489000	-0.681523000
H	5.947855000	-0.426074000	1.087934000
C	-1.749266000	-2.856320000	2.025112000
H	-1.147172000	-3.315824000	2.825266000
H	-2.759929000	-2.690629000	2.429976000
H	-1.841708000	-3.600781000	1.218726000
C	-1.667343000	-2.903932000	-1.783045000
H	-2.689667000	-3.259348000	-1.576241000
H	-1.393919000	-3.262652000	-2.787971000
H	-0.995578000	-3.401614000	-1.066066000
C	-4.885023000	-0.225795000	-0.748834000
H	-5.964002000	-0.355772000	-0.568863000
H	-4.684792000	0.857274000	-0.783035000
H	-4.670586000	-0.627663000	-1.752002000

Zero-point correction = 0.371276 (Hartree/Particle)
Thermal correction to Energy = 0.397548
Thermal correction to Enthalpy = 0.398492
Thermal correction to Gibbs Free Energy = 0.307967
Sum of electronic and zero-point Energies = -5478.883741
Sum of electronic and thermal Energies = -5478.857468
Sum of electronic and thermal Enthalpies = -5478.856524
Sum of electronic and thermal Free Energies = -5478.947049
E(RM06) = -846.143579147

TS_H-PhGeEt$_3$

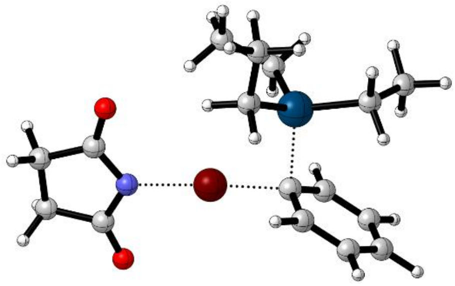

Ge	1.764934000	0.884370000	-0.017289000
C	1.106732000	-1.166488000	0.143173000
C	1.654886000	-1.797144000	-1.004818000
C	1.542732000	-1.621383000	1.416784000

C	2.638793000	-2.769908000	-0.888580000
C	2.524199000	-2.592929000	1.534397000
C	3.076688000	-3.159098000	0.379796000
H	1.307768000	-1.499066000	-1.997812000
H	1.102217000	-1.187393000	2.317927000
H	3.065031000	-3.229090000	-1.781805000
H	2.859906000	-2.919582000	2.519785000
H	3.851843000	-3.922827000	0.471816000
C	3.715851000	0.706187000	-0.001869000
H	4.100875000	1.614344000	0.491106000
H	3.969004000	-0.131215000	0.668003000
C	1.055867000	1.579113000	-1.707609000
H	0.685806000	0.732564000	-2.305602000
H	1.918783000	1.986752000	-2.258445000
C	1.068527000	1.795942000	1.566631000
H	-0.017524000	1.906099000	1.430036000
H	1.219153000	1.153756000	2.447233000
Br	-0.963504000	-0.689918000	0.043046000
C	-5.191435000	0.925729000	0.111941000
C	-3.663984000	0.979321000	0.159703000
N	-3.153844000	-0.273736000	-0.019967000
C	-4.130677000	-1.216918000	-0.186199000
C	-5.504275000	-0.546311000	-0.126926000
H	-5.589947000	1.317267000	1.059260000
O	-3.014786000	1.997700000	0.331783000
O	-3.946055000	-2.407821000	-0.356460000
H	-6.034278000	-0.740384000	-1.070854000
H	-5.544888000	1.592543000	-0.687941000
H	-6.092579000	-1.012981000	0.676486000
C	1.734951000	3.157201000	1.786300000
H	1.290619000	3.669835000	2.652906000
H	2.813654000	3.059547000	1.981365000
H	1.614953000	3.823299000	0.917908000
C	-0.035372000	2.638658000	-1.549103000
H	0.340451000	3.531107000	-1.025550000
H	-0.395823000	2.969995000	-2.535323000
H	-0.900788000	2.261289000	-0.983290000
C	4.372017000	0.526553000	-1.369766000
H	5.459087000	0.387010000	-1.268304000
H	3.978763000	-0.352784000	-1.902463000
H	4.213305000	1.403183000	-2.015728000

Zero-point correction = 0.370885 (Hartree/Particle)
Thermal correction to Energy = 0.396202
Thermal correction to Enthalpy = 0.397146
Thermal correction to Gibbs Free Energy = 0.312839
Sum of electronic and zero-point Energies = -5478.838808
Sum of electronic and thermal Energies = -5478.813491
Sum of electronic and thermal Enthalpies = -5478.812546
Sum of electronic and thermal Free Energies = -5478.896853

E(RM06) = -846.117757712

TS_Me-PhGeEt$_3$

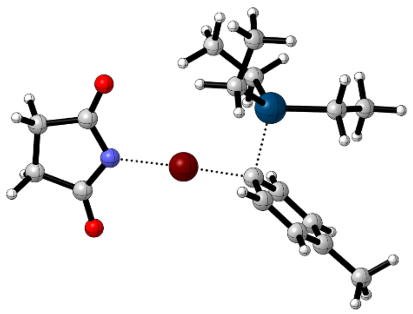

Ge	-1.478343000	-1.252694000	-0.018776000
C	-1.072354000	0.834093000	0.085801000
C	-1.667693000	1.391442000	-1.079915000
C	-1.570210000	1.288534000	1.339860000
C	-2.736558000	2.266448000	-0.998055000
C	-2.638597000	2.161026000	1.419786000
C	-3.249998000	2.654319000	0.251458000
H	-1.284152000	1.107773000	-2.063836000
H	-1.103773000	0.927714000	2.260249000
H	-3.186260000	2.661656000	-1.911284000
H	-3.010034000	2.478594000	2.396314000
C	-3.437674000	-1.324459000	-0.020113000
H	-3.709764000	-2.275510000	0.467348000
H	-3.798662000	-0.527125000	0.649766000
C	-0.676934000	-1.916321000	-1.681621000
H	-0.422407000	-1.052202000	-2.314244000
H	-1.474795000	-2.458289000	-2.214602000
C	-0.705227000	-2.047545000	1.593537000
H	0.388948000	-2.028925000	1.480280000
H	-0.948387000	-1.412871000	2.458982000
Br	1.049736000	0.660925000	0.015348000
C	5.462862000	-0.351119000	0.178084000
C	3.957192000	-0.617490000	0.205022000
N	3.279933000	0.546022000	-0.019041000
C	4.118044000	1.611602000	-0.200606000
C	5.570887000	1.142282000	-0.103831000
H	5.893458000	-0.653620000	1.143836000
O	3.454369000	-1.711963000	0.398078000
O	3.772173000	2.759886000	-0.408630000
H	6.083585000	1.380067000	-1.047355000
H	5.921970000	-0.985681000	-0.593858000
H	6.075402000	1.710808000	0.690892000
C	-1.205287000	-3.475379000	1.830935000
H	-0.715604000	-3.917555000	2.711893000
H	-2.290690000	-3.504966000	2.010584000
H	-0.994542000	-4.136130000	0.975737000

C	0.546489000	-2.813268000	-1.487806000
H	0.294731000	-3.724929000	-0.924540000
H	0.947571000	-3.134898000	-2.461550000
H	1.354176000	-2.300509000	-0.944113000
C	-4.104724000	-1.224892000	-1.391157000
H	-5.201386000	-1.241232000	-1.297077000
H	-3.836603000	-0.293666000	-1.912899000
H	-3.820262000	-2.063633000	-2.044381000
C	-4.438329000	3.561730000	0.338638000
H	-4.417511000	4.166014000	1.255335000
H	-4.503272000	4.226018000	-0.533273000
H	-5.358704000	2.955373000	0.363050000

Zero-point correction = 0.398110 (Hartree/Particle)
Thermal correction to Energy = 0.425339
Thermal correction to Enthalpy = 0.426283
Thermal correction to Gibbs Free Energy = 0.336695
Sum of electronic and zero-point Energies = -5518.094361
Sum of electronic and thermal Energies = -5518.067132
Sum of electronic and thermal Enthalpies = -5518.066188
Sum of electronic and thermal Free Energies = -5518.155776
E(RM06) = -885.416092283

TS_F-PhGeEt$_3$

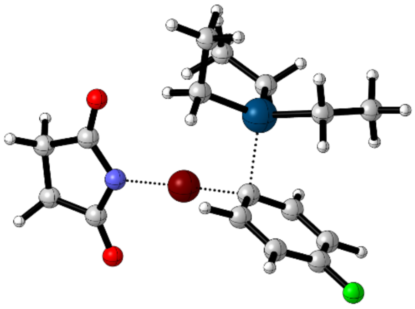

Ge	1.491770000	1.221488000	-0.026586000
C	1.067495000	-0.891543000	0.092686000
C	1.679610000	-1.435724000	-1.071031000
C	1.568907000	-1.320871000	1.354600000
C	2.772645000	-2.280331000	-0.990670000
C	2.659486000	-2.163070000	1.455092000
C	3.249380000	-2.622892000	0.274838000
H	1.290815000	-1.167932000	-2.056834000
H	1.087241000	-0.966394000	2.269078000
H	3.259638000	-2.681561000	-1.879820000
H	3.058919000	-2.480703000	2.418598000
C	3.449903000	1.274583000	-0.020140000
H	3.724429000	2.213223000	0.489448000
H	3.806481000	0.463683000	0.635378000
C	0.691997000	1.856434000	-1.699623000
H	0.430901000	0.983445000	-2.316752000
H	1.492411000	2.384299000	-2.242613000
C	0.700241000	2.011868000	1.576998000
H	-0.392312000	1.988580000	1.449768000
H	0.937444000	1.380759000	2.446441000
Br	-1.031234000	-0.685513000	0.016168000
C	-5.431514000	0.439604000	0.198752000
C	-3.917858000	0.657777000	0.233116000
N	-3.275375000	-0.518985000	-0.017722000
C	-4.145726000	-1.552700000	-0.224585000
C	-5.584564000	-1.042547000	-0.118720000
H	-5.854192000	0.732204000	1.171068000
O	-3.382498000	1.732444000	0.452928000
O	-3.835320000	-2.706595000	-0.458784000
H	-6.102222000	-1.242174000	-1.068406000
H	-5.869601000	1.106304000	-0.558224000
H	-6.107884000	-1.614276000	0.661459000
C	1.194672000	3.442031000	1.813587000
H	0.694521000	3.883960000	2.688611000
H	2.278036000	3.475687000	2.004058000
H	0.989183000	4.099734000	0.954895000
C	-0.526124000	2.762389000	-1.515007000
H	-0.265997000	3.684888000	-0.973778000
H	-0.932692000	3.064029000	-2.492671000
H	-1.331054000	2.264244000	-0.953751000
C	4.119480000	1.201824000	-1.391621000
H	5.215637000	1.203711000	-1.292841000
H	3.843521000	0.287506000	-1.938682000
H	3.846303000	2.060154000	-2.023611000
F	4.293221000	-3.423656000	0.361701000

Zero-point correction = 0.362822 (Hartree/Particle)
Thermal correction to Energy = 0.388924
Thermal correction to Enthalpy = 0.389868
Thermal correction to Gibbs Free Energy = 0.303567
Sum of electronic and zero-point Energies = -5577.981256
Sum of electronic and thermal Energies = -5577.955155
Sum of electronic and thermal Enthalpies = -5577.954211
Sum of electronic and thermal Free Energies = -5578.040512
E(RM06) = -945.360211265

TS_I-PhGeEt$_3$

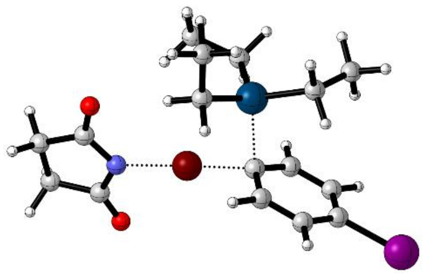

Ge	-0.179069000	1.935830000	-0.055096000
C	0.298402000	-0.192950000	0.003431000
C	1.038929000	-0.408291000	-1.185997000
C	0.970910000	-0.395263000	1.236289000
C	2.390604000	-0.721753000	-1.153215000
C	2.319868000	-0.706393000	1.285240000
C	3.028520000	-0.855056000	0.084917000
H	0.545328000	-0.307901000	-2.156217000
H	0.418832000	-0.287929000	2.173310000
H	2.941982000	-0.858318000	-2.084267000
H	2.817101000	-0.834839000	2.247383000
C	1.595720000	2.763095000	-0.073729000
H	1.476073000	3.729550000	0.444207000
H	2.256833000	2.158931000	0.567937000
C	-1.208060000	2.228032000	-1.696684000
H	-1.124182000	1.330451000	-2.327535000
H	-0.693224000	3.034489000	-2.243276000
C	-1.181865000	2.287735000	1.585381000
H	-2.176955000	1.834290000	1.461604000
H	-0.692783000	1.771482000	2.425011000
Br	-1.734337000	-0.785746000	-0.015643000
C	-6.201444000	-1.328873000	0.376744000
C	-4.856110000	-0.603607000	0.424644000
N	-3.863805000	-1.447957000	0.019864000
C	-4.334491000	-2.688716000	-0.310722000
C	-5.851691000	-2.727225000	-0.117767000
H	-6.651114000	-1.310567000	1.380178000
O	-4.708825000	0.556891000	0.772565000
O	-3.658848000	-3.623359000	-0.699668000
H	-6.324628000	-2.991745000	-1.074766000
H	-6.878221000	-0.776417000	-0.291256000
H	-6.097847000	-3.526257000	0.596686000
C	-1.290457000	3.787258000	1.878180000
H	-1.904639000	3.960854000	2.774735000
H	-0.305989000	4.243261000	2.062482000
H	-1.761395000	4.339498000	1.050213000
C	-2.677445000	2.581948000	-1.463331000
H	-2.783163000	3.531961000	-0.917590000
H	-3.201950000	2.699320000	-2.424047000
H	-3.201336000	1.807160000	-0.883329000
C	2.216842000	2.975816000	-1.453073000
H	3.220077000	3.419702000	-1.365314000
H	2.326295000	2.031135000	-2.006714000
H	1.611264000	3.654412000	-2.072392000
I	5.076496000	-1.290662000	0.145838000

Zero-point correction = 0.360623 (Hartree/Particle)
Thermal correction to Energy = 0.387428
Thermal correction to Enthalpy = 0.388372
Thermal correction to Gibbs Free Energy = 0.299156
Sum of electronic and zero-point Energies = -5776.020455
Sum of electronic and thermal Energies = -5775.993650
Sum of electronic and thermal Enthalpies = -5775.992706
Sum of electronic and thermal Free Energies = -5776.081922
E(RM06) = -856.905220923

TS_OMe-PhGeEt$_3$

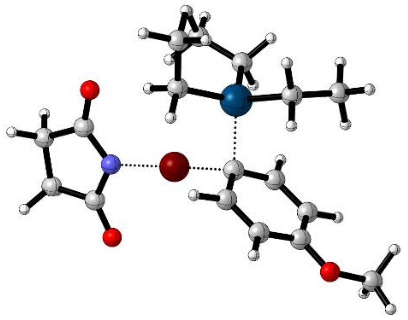

Ge	-1.163816000	-1.613070000	0.010859000
C	-1.008623000	0.466931000	0.134238000
C	-1.655673000	1.034936000	-1.008733000
C	-1.512454000	0.887475000	1.412649000
C	-2.746953000	1.869018000	-0.910483000
C	-2.601124000	1.708777000	1.533311000
C	-3.241772000	2.205283000	0.369839000
H	-1.278528000	0.787971000	-2.004912000
H	-1.017494000	0.528754000	2.318713000
H	-3.214139000	2.257929000	-1.814003000
H	-2.990857000	2.007713000	2.506916000
C	-3.087834000	-1.978540000	0.096843000
H	-3.180870000	-3.028876000	0.420848000
H	-3.508186000	-1.370332000	0.914529000
C	-0.367068000	-2.173100000	-1.693199000
H	-0.289845000	-1.292911000	-2.350362000
H	-1.102036000	-2.841809000	-2.170535000
C	-0.227886000	-2.331622000	1.573638000
H	0.849048000	-2.165212000	1.420977000
H	-0.514706000	-1.747953000	2.461918000
Br	1.094424000	0.640470000	0.022885000
C	5.666867000	0.261974000	-0.001654000
C	4.213915000	-0.218709000	-0.019226000

N	3.369736000	0.851173000	-0.049638000
C	4.043905000	2.039653000	-0.049318000
C	5.552720000	1.780340000	-0.032589000
H	6.159219000	-0.129187000	0.900767000
O	3.882607000	-1.393980000	-0.007746000
O	3.535206000	3.146736000	-0.060830000
H	6.001749000	2.241827000	-0.924388000
H	6.193267000	-0.164720000	-0.867913000
H	5.989919000	2.279302000	0.844592000
C	-0.522846000	-3.816161000	1.804535000
H	0.060500000	-4.204087000	2.653578000
H	-1.585707000	-3.991096000	2.030575000
H	-0.268148000	-4.430345000	0.926754000
C	0.990476000	-2.867858000	-1.574741000
H	0.921771000	-3.790272000	-0.977642000
H	1.366976000	-3.154111000	-2.569346000
H	1.748016000	-2.224752000	-1.102168000
C	-3.865354000	-1.760142000	-1.200752000
H	-4.930251000	-2.007400000	-1.070552000
H	-3.812349000	-0.715141000	-1.540173000
H	-3.480643000	-2.391610000	-2.016252000
O	-4.283917000	2.981397000	0.575446000
C	-5.003632000	3.535310000	-0.517929000
H	-4.357933000	4.192353000	-1.118334000
H	-5.425279000	2.740243000	-1.149963000
H	-5.815514000	4.122701000	-0.078161000

Zero-point correction = 0.403776 (Hartree/Particle)

Thermal correction to Energy = 0.431711

Thermal correction to Enthalpy = 0.432655

Thermal correction to Gibbs Free Energy = 0.341687

Sum of electronic and zero-point Energies = -5593.222083

Sum of electronic and thermal Energies = -5593.194147

Sum of electronic and thermal Enthalpies = -5593.193203

Sum of electronic and thermal Free Energies = -5593.284171

E(RM06) = -960.621798735

pi_NBS-GeEt$_3$_PhBr

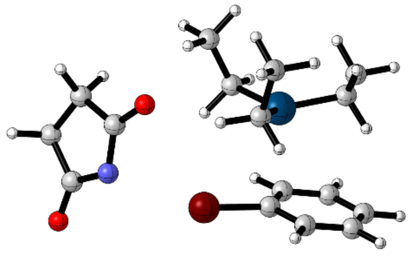

Ge	1.432773000	1.051361000	0.047577000
C	1.125429000	-1.737479000	0.069925000
C	1.621249000	-1.603144000	-1.239412000
C	1.996363000	-2.060147000	1.124654000
C	2.979484000	-1.823640000	-1.488876000
C	3.346481000	-2.260826000	0.859903000
C	3.838408000	-2.154281000	-0.445888000
H	0.939206000	-1.388649000	-2.064346000
H	1.607626000	-2.170231000	2.138247000
H	3.354990000	-1.743104000	-2.510179000
H	4.020936000	-2.514270000	1.679549000
H	4.897186000	-2.327823000	-0.644632000
C	3.336511000	1.310748000	-0.171759000
H	3.567478000	2.141409000	0.517818000
H	3.846683000	0.432078000	0.254625000
C	0.256865000	1.467278000	-1.435746000
H	-0.520955000	0.690945000	-1.482791000
H	0.841937000	1.423786000	-2.365932000
C	0.733358000	1.214312000	1.846301000
H	-0.356307000	1.069123000	1.777594000
H	1.169815000	0.420136000	2.469389000
Br	-0.745342000	-1.619886000	0.379391000
C	-4.043466000	1.451512000	-0.669290000
C	-3.187535000	0.834439000	0.445608000
N	-3.412541000	-0.490692000	0.587013000
C	-4.368204000	-0.892237000	-0.294399000
C	-4.900183000	0.284830000	-1.130383000
H	-4.608163000	2.308399000	-0.273056000
O	-2.384357000	1.505446000	1.099258000
O	-4.778873000	-2.039258000	-0.429462000
H	-4.805862000	0.044861000	-2.199903000
H	-3.372092000	1.841594000	-1.450223000
H	-5.973533000	0.414037000	-0.922568000
C	1.063359000	2.599644000	2.421760000
H	0.625420000	2.689504000	3.426800000
H	2.145713000	2.771439000	2.518380000
H	0.642648000	3.413198000	1.811497000
C	-0.388703000	2.844669000	-1.223215000
H	0.355777000	3.648084000	-1.112774000
H	-1.009220000	3.095606000	-2.096518000
H	-1.044662000	2.830041000	-0.339459000
C	3.806701000	1.618773000	-1.590139000
H	4.894231000	1.780102000	-1.604825000
H	3.585915000	0.793579000	-2.282216000
H	3.330374000	2.527245000	-1.987369000

Zero-point correction = 0.371284 (Hartree/Particle)

Thermal correction to Energy = 0.397544

Thermal correction to Enthalpy = 0.398489

Thermal correction to Gibbs Free Energy = 0.310182

Sum of electronic and zero-point Energies = -5478.848753

Sum of electronic and thermal Energies = -5478.822492

Sum of electronic and thermal Enthalpies = -5478.821548

Sum of electronic and thermal Free Energies = -5478.909854

E(RM06) = -846.129622349

PhBr

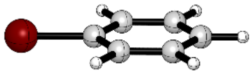

C	0.000000000	0.000000000	-0.094676000
C	0.000000000	1.215429000	-0.779485000
C	0.000000000	-1.215429000	-0.779485000
C	0.000000000	1.207249000	-2.174405000
C	0.000000000	-1.207249000	-2.174405000
C	0.000000000	0.000000000	-2.873697000
H	0.000000000	2.158559000	-0.230406000
H	0.000000000	-2.158559000	-0.230406000
H	0.000000000	2.156016000	-2.714999000
H	0.000000000	-2.156016000	-2.714999000
H	0.000000000	0.000000000	-3.965389000
Br	0.000000000	0.000000000	1.803232000

Zero-point correction = 0.091414 (Hartree/Particle)

Thermal correction to Energy = 0.097052

Thermal correction to Enthalpy = 0.097996

Thermal correction to Gibbs Free Energy = 0.061238

Sum of electronic and zero-point Energies = -2805.214495

Sum of electronic and thermal Energies = -2805.208857

Sum of electronic and thermal Enthalpies = -2805.207912

Sum of electronic and thermal Free Energies = -2805.244671

E(RM06) = -244.863206182

NBS-GeEt3

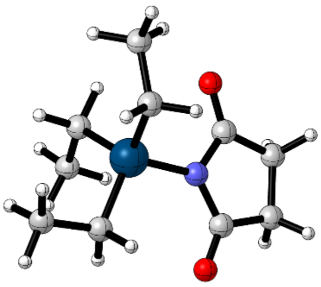

Ge	0.822916000	0.023180000	-0.120622000
C	1.320653000	1.404303000	-1.415159000
H	2.222085000	1.036598000	-1.933245000
H	0.524454000	1.436772000	-2.176580000
C	1.233710000	0.478254000	1.733245000
H	0.786996000	1.461940000	1.941382000
H	2.326714000	0.605670000	1.806055000
C	1.564322000	-1.718618000	-0.597950000
H	1.085099000	-2.486248000	0.026381000
H	1.276794000	-1.946007000	-1.635809000
C	-3.344382000	-0.866638000	-0.320373000
C	-1.873954000	-1.228449000	-0.459965000
N	-1.102435000	-0.114942000	-0.157649000
C	-1.884389000	0.979637000	0.169180000
C	-3.351044000	0.598720000	0.093368000
H	-3.844295000	-1.059544000	-1.279977000
O	-1.443387000	-2.311284000	-0.783241000
O	-1.443961000	2.068792000	0.467896000
H	-3.811917000	0.781885000	1.074201000
H	-3.798191000	-1.538261000	0.422033000
H	-3.848890000	1.261958000	-0.627975000
C	3.085285000	-1.741262000	-0.429404000
H	3.504189000	-2.716394000	-0.721987000
H	3.580894000	-0.976179000	-1.048269000
H	3.381739000	-1.559382000	0.615871000
C	0.741783000	-0.569871000	2.731483000
H	1.205507000	-1.552643000	2.551151000
H	0.976031000	-0.281955000	3.767982000
H	-0.349450000	-0.706033000	2.666488000
C	1.568577000	2.793563000	-0.827556000
H	1.849756000	3.513727000	-1.611841000
H	0.667592000	3.170202000	-0.323282000
H	2.384034000	2.778845000	-0.087582000

Zero-point correction = 0.279986 (Hartree/Particle)

Thermal correction to Energy = 0.298105

Thermal correction to Enthalpy = 0.299049

Thermal correction to Gibbs Free Energy = 0.232288

Sum of electronic and zero-point Energies = -2673.719832

Sum of electronic and thermal Energies = -2673.701713

Sum of electronic and thermal Enthalpies = -2673.700769

Sum of electronic and thermal Free Energies = -2673.767530

E(RM06) = -601.332435765

PhSiMe3

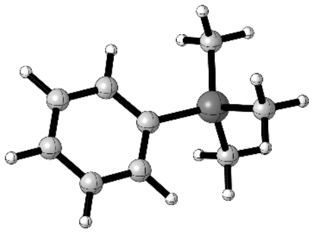

Si	1.472342000	0.007227000	0.000002000
C	-0.421734000	0.023450000	-0.000091000

C	-1.136605000	-1.187631000	-0.000104000
C	-1.163050000	1.215737000	-0.000045000
C	-2.531189000	-1.209303000	-0.000046000
C	-2.559832000	1.203202000	0.000011000
C	-3.246862000	-0.010382000	0.000016000
H	-0.596916000	-2.140439000	-0.000155000
H	-0.646337000	2.179783000	-0.000081000
H	-3.062917000	-2.163908000	-0.000056000
H	-3.113486000	2.145269000	0.000045000
C	2.068591000	-0.899576000	1.537879000
H	3.169018000	-0.951705000	1.558109000
H	1.733055000	-0.385715000	2.452660000
H	1.680236000	-1.930156000	1.565153000
C	2.068853000	-0.902375000	-1.536109000
H	1.731596000	-0.391467000	-2.451904000
H	3.169363000	-0.952452000	-1.557107000
H	1.682491000	-1.933777000	-1.560466000
C	2.120144000	1.771773000	-0.001595000
H	3.221776000	1.768903000	-0.000678000
H	1.785115000	2.323117000	-0.894534000
H	1.783685000	2.325345000	0.889424000
H	-4.339371000	-0.023338000	0.000063000

Zero-point correction = 0.202523 (Hartree/Particle)
Thermal correction to Energy = 0.214641
Thermal correction to Enthalpy = 0.215585
Thermal correction to Gibbs Free Energy = 0.164415
Sum of electronic and zero-point Energies = -640.269566
Sum of electronic and thermal Energies = -640.257448
Sum of electronic and thermal Enthalpies = -640.256503
Sum of electronic and thermal Free Energies = -640.307673
E(RM06) = -640.716740912

pi_NBS_PhSiMe$_3$

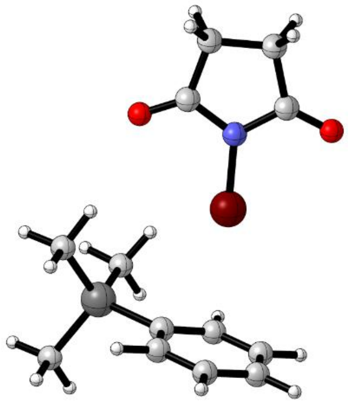

Si	2.713191000	1.419135000	0.215243000
C	2.472666000	-0.439955000	-0.078235000
C	2.606738000	-0.977221000	-1.370692000
C	2.159965000	-1.329010000	0.965261000
C	2.431477000	-2.338979000	-1.614307000
C	1.979173000	-2.695159000	0.727870000
C	2.113718000	-3.201989000	-0.563775000
H	2.848577000	-0.320077000	-2.211981000
H	2.053371000	-0.955299000	1.987903000
H	2.538552000	-2.729843000	-2.628948000
H	1.731244000	-3.363161000	1.555994000
C	4.538830000	1.822489000	-0.002028000
H	4.721212000	2.899449000	0.143089000
H	5.154125000	1.269474000	0.725170000
C	1.698194000	2.376286000	-1.045249000
H	1.978337000	2.102102000	-2.074813000
H	1.872696000	3.458241000	-0.928182000
C	2.155615000	1.850685000	1.957955000
H	1.097086000	1.588288000	2.113570000
H	2.755898000	1.327158000	2.718834000
Br	-0.837184000	-0.516002000	0.262885000
C	-4.354497000	1.458369000	-0.550927000
C	-2.846261000	1.346988000	-0.483376000
N	-2.558958000	0.070564000	0.002527000
C	-3.677661000	-0.720038000	0.281359000
C	-4.894178000	0.114361000	-0.059681000
H	-4.665124000	2.308461000	0.072226000
O	-2.023789000	2.172325000	-0.779525000
O	-3.633630000	-1.841796000	0.708135000
H	-5.480168000	-0.424202000	-0.817323000
H	-4.637924000	1.692232000	-1.586425000
H	-5.520156000	0.196037000	0.839687000
H	1.970472000	-4.268221000	-0.753096000
H	0.620610000	2.186447000	-0.918832000
H	4.882076000	1.552779000	-1.013469000
H	2.265273000	2.933185000	2.130575000

Zero-point correction = 0.285616 (Hartree/Particle)
Thermal correction to Energy = 0.307161
Thermal correction to Enthalpy = 0.308105
Thermal correction to Gibbs Free Energy = 0.230771
Sum of electronic and zero-point Energies = -3573.761875
Sum of electronic and thermal Energies = -3573.740331
Sum of electronic and thermal Enthalpies = -3573.739387
Sum of electronic and thermal Free Energies = -3573.816720
E(RM06) = -1013.99961232

TS_PhSiMe$_3$

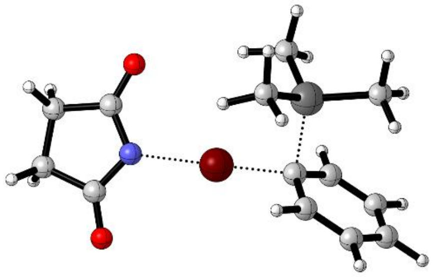

Si	-2.071316000	1.468952000	-0.233188000
C	-1.646289000	-0.543948000	0.070359000
C	-2.216030000	-0.794527000	1.353329000
C	-2.247995000	-1.181127000	-1.054582000
C	-3.373079000	-1.544198000	1.488173000
C	-3.403622000	-1.932077000	-0.917792000
C	-3.966088000	-2.105038000	0.351991000
H	-1.741457000	-0.363196000	2.238443000
H	-1.795664000	-1.052967000	-2.041016000
H	-3.813260000	-1.704249000	2.473127000
H	-3.866288000	-2.394028000	-1.790780000
C	-3.920341000	1.634867000	-0.042254000
H	-4.201303000	2.656314000	-0.346691000
H	-4.470275000	0.930250000	-0.683559000
C	-1.152407000	2.443365000	1.063011000
H	-1.403243000	2.120575000	2.083935000
H	-1.472597000	3.493312000	0.955834000
C	-1.521001000	1.872160000	-1.968276000
H	-0.467719000	1.611148000	-2.141315000
H	-2.147219000	1.376817000	-2.724335000
Br	0.418920000	-0.404383000	0.027548000
C	4.851982000	0.822132000	0.205876000
C	3.329459000	0.970302000	0.278987000
N	2.730184000	-0.228781000	0.033405000
C	3.640283000	-1.216716000	-0.206414000
C	5.061344000	-0.651194000	-0.117830000
H	5.239977000	1.507079000	-0.562374000
O	2.756116000	2.021025000	0.523630000
O	3.378592000	-2.381417000	-0.455416000
H	5.615953000	-1.202911000	0.655456000
H	5.286950000	1.134506000	1.166646000
H	5.575685000	-0.829486000	-1.073610000
H	-4.877298000	-2.697372000	0.459043000
H	-0.063558000	2.390788000	0.922717000
H	-4.238969000	1.490622000	1.000251000
H	-1.629716000	2.961470000	-2.098345000

Zero-point correction = 0.284822 (Hartree/Particle)

Thermal correction to Energy = 0.305548

Thermal correction to Enthalpy = 0.306492

Thermal correction to Gibbs Free Energy = 0.233022

Sum of electronic and zero-point Energies = -3573.711712

Sum of electronic and thermal Energies = -3573.690986

Sum of electronic and thermal Enthalpies = -3573.690042

Sum of electronic and thermal Free Energies = -3573.763512

E(RM06) = -1013.96822094

pi_NBS-SiMe$_3$_PhBr

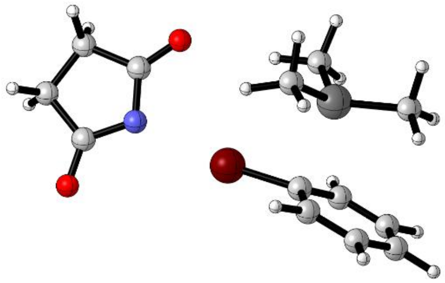

Si	2.037726000	1.462567000	0.276215000
C	1.633601000	-0.802016000	-0.113071000
C	2.285824000	-0.799628000	-1.372320000
C	2.351320000	-1.234500000	1.030883000
C	3.621971000	-1.169050000	-1.467991000
C	3.687251000	-1.598259000	0.920587000
C	4.317210000	-1.573416000	-0.326474000
H	1.726990000	-0.513677000	-2.265624000
H	1.842538000	-1.286883000	1.995181000
H	4.118616000	-1.152620000	-2.438880000
H	4.235351000	-1.917266000	1.807968000
C	3.853328000	1.772002000	0.088458000
H	3.989140000	2.824444000	0.393727000
H	4.472760000	1.153229000	0.751033000
C	0.986136000	2.280995000	-1.008963000
H	1.279702000	2.006181000	-2.031565000
H	1.174418000	3.362100000	-0.885392000
C	1.424604000	1.664180000	2.014484000
H	0.420603000	1.241292000	2.154796000
H	2.118650000	1.233258000	2.749123000
Br	-0.295082000	-0.738743000	-0.062638000
C	-4.800202000	1.171791000	-0.186677000
C	-3.279783000	0.976992000	-0.292204000
N	-2.929232000	-0.313684000	-0.070438000
C	-4.028672000	-1.070226000	0.184995000
C	-5.310951000	-0.223442000	0.134658000
H	-5.013384000	1.918482000	0.593091000
O	-2.512680000	1.902523000	-0.547231000
O	-4.031588000	-2.271231000	0.427148000
H	-5.986957000	-0.639995000	-0.627255000

H	-5.177573000	1.581352000	-1.135771000
H	-5.830333000	-0.299787000	1.101800000
H	5.363816000	-1.871944000	-0.408627000
H	-0.088130000	2.092805000	-0.856942000
H	4.199425000	1.670787000	-0.948386000
H	1.363021000	2.751227000	2.192571000

Zero-point correction = 0.284834 (Hartree/Particle)

Thermal correction to Energy = 0.306504

Thermal correction to Enthalpy = 0.307449

Thermal correction to Gibbs Free Energy = 0.230888

Sum of electronic and zero-point Energies = -3573.714058

Sum of electronic and thermal Energies = -3573.692388

Sum of electronic and thermal Enthalpies = -3573.691444

Sum of electronic and thermal Free Energies = -3573.768005

E(RM06) = -1013.96883132

NMR Spectra

Triethyl(phenyl)germane

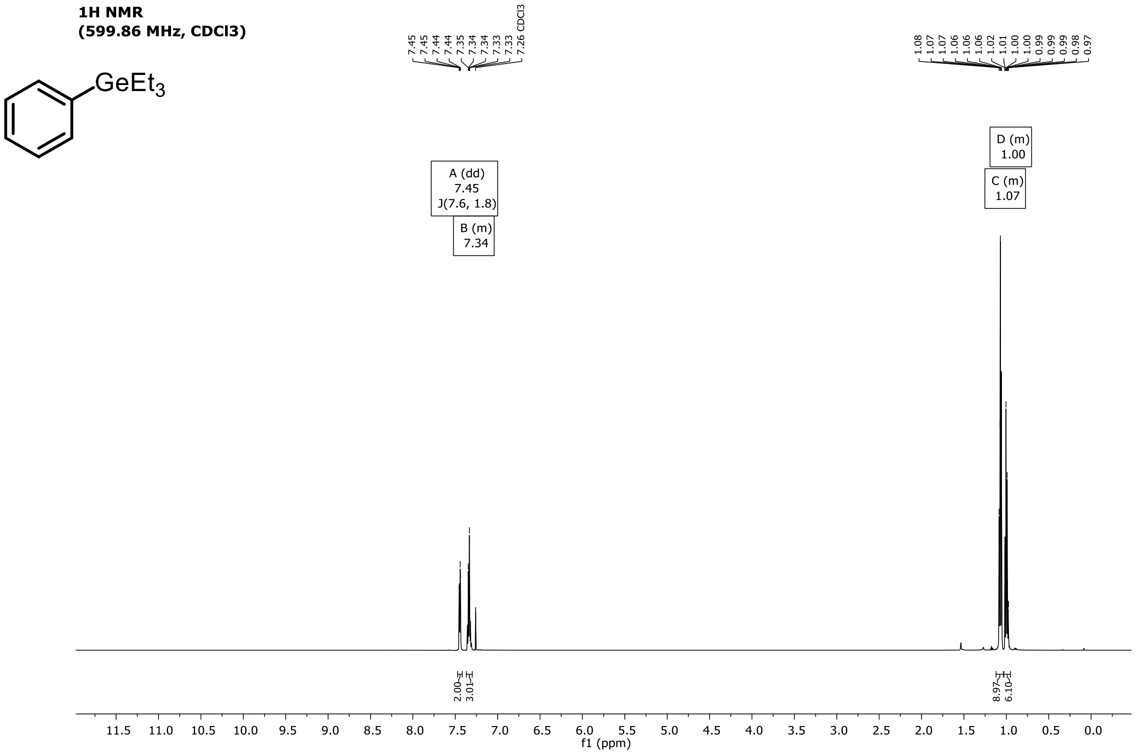

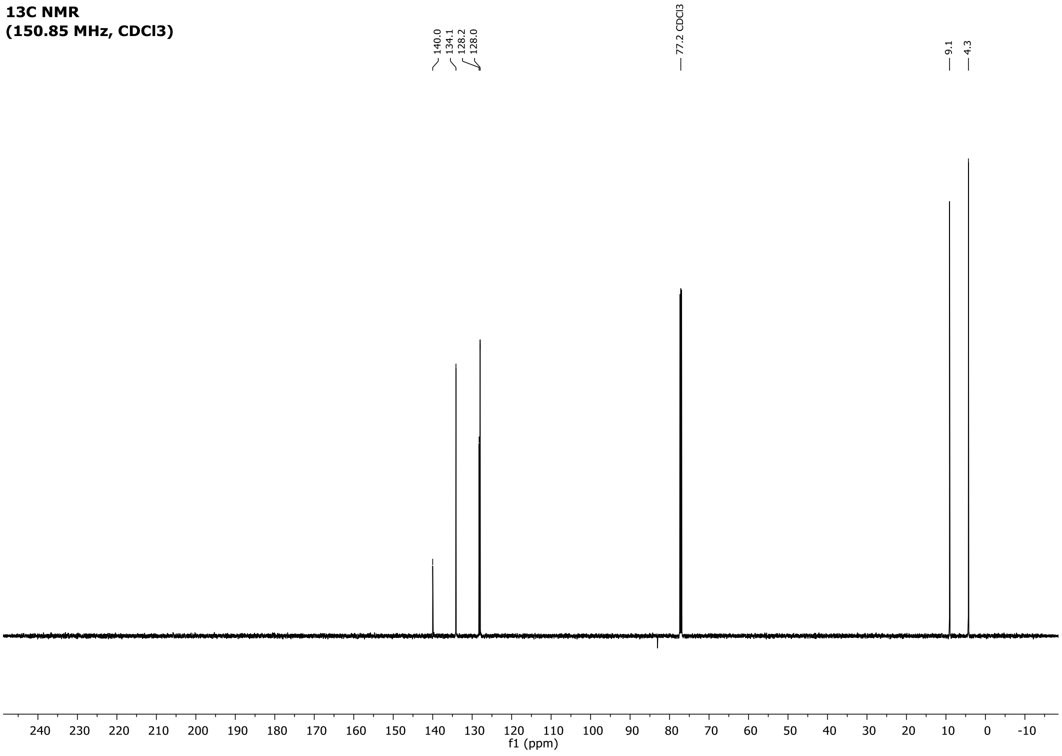

Triethyl(4-fluorophenyl)germane

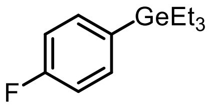

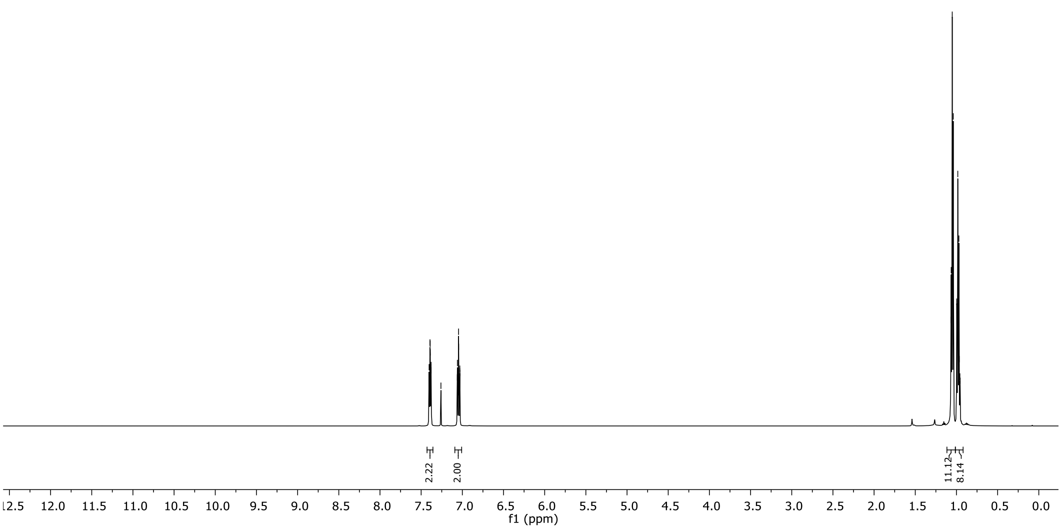

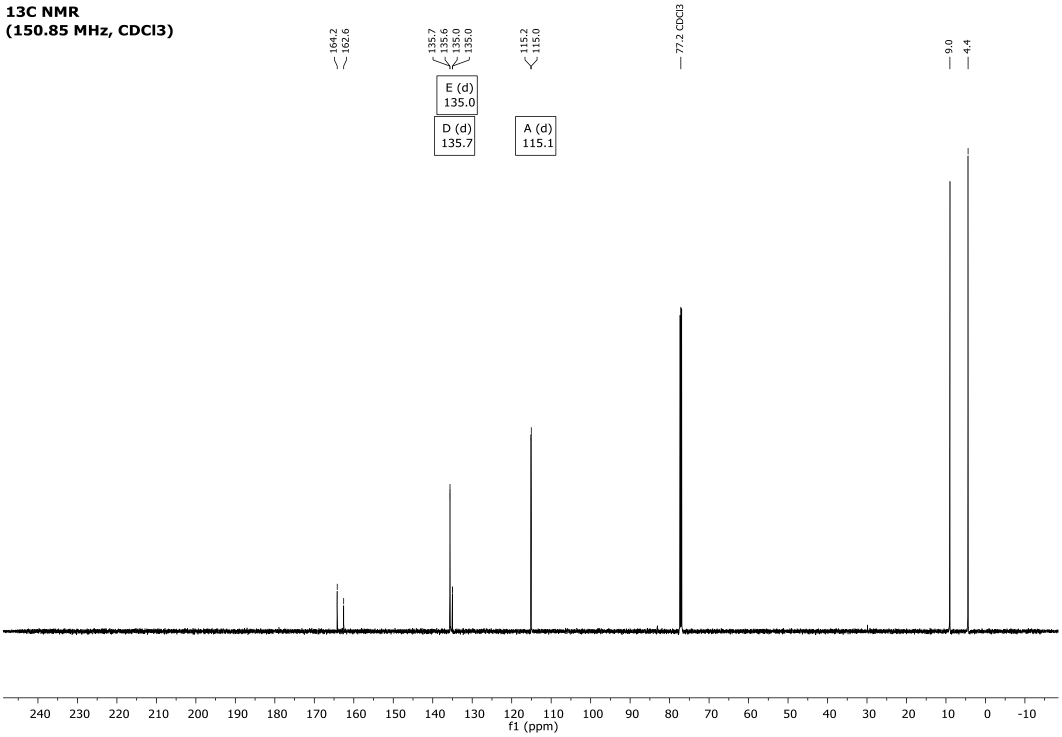

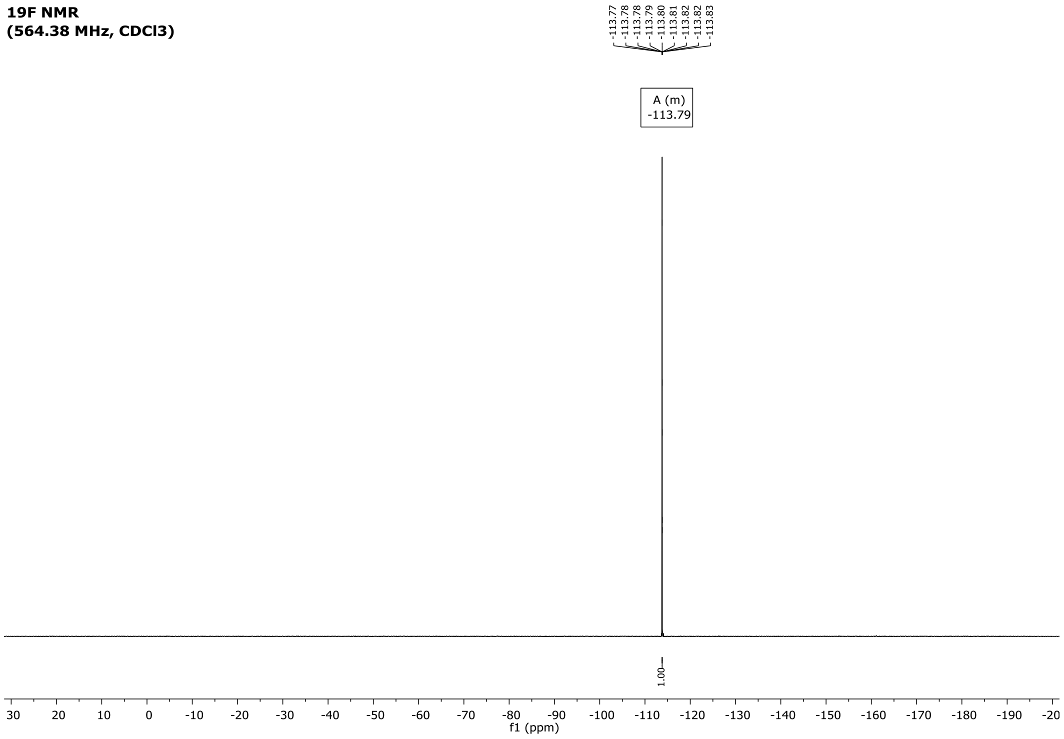
19F NMR
(564.38 MHz, CDCl3)
-113.77
-113.78
-113.78
-113.79
-113.80
-113.81
-113.82
-113.82
-113.83
A (m)
-113.79
1.00
30
20
10
0
-10
-20
-30
-40
-50
-60
-70
-80
-90
-100
-110
-120
-130
-140
-150
-160
-170
-180
-190
-20
f1 (ppm)

Triethyl(4-methoxyphenyl)germane

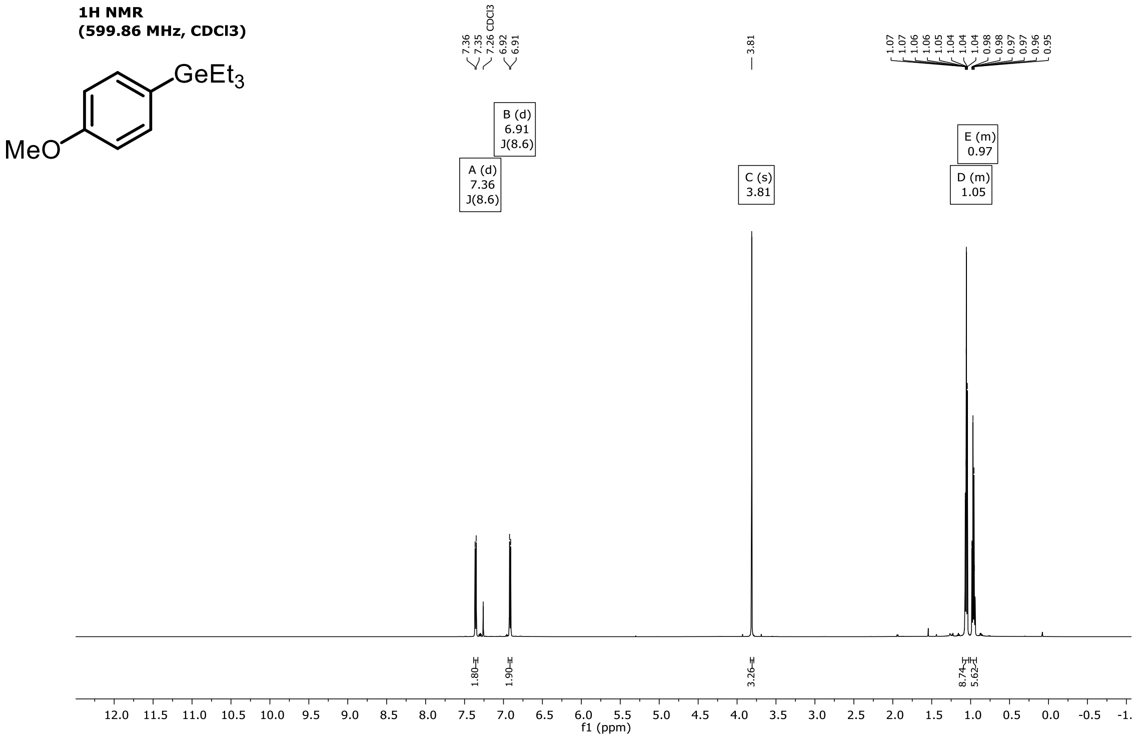

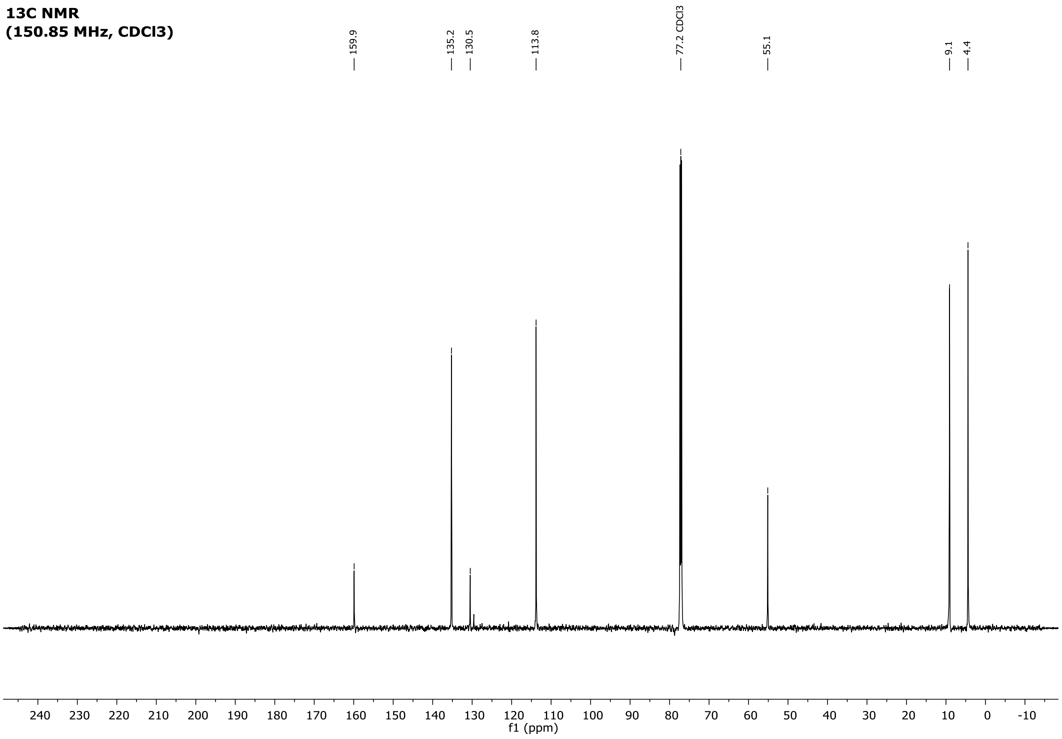

Triethyl(mesityl)germane

1H NMR
(599.86 MHz, CDCl3)

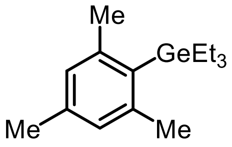

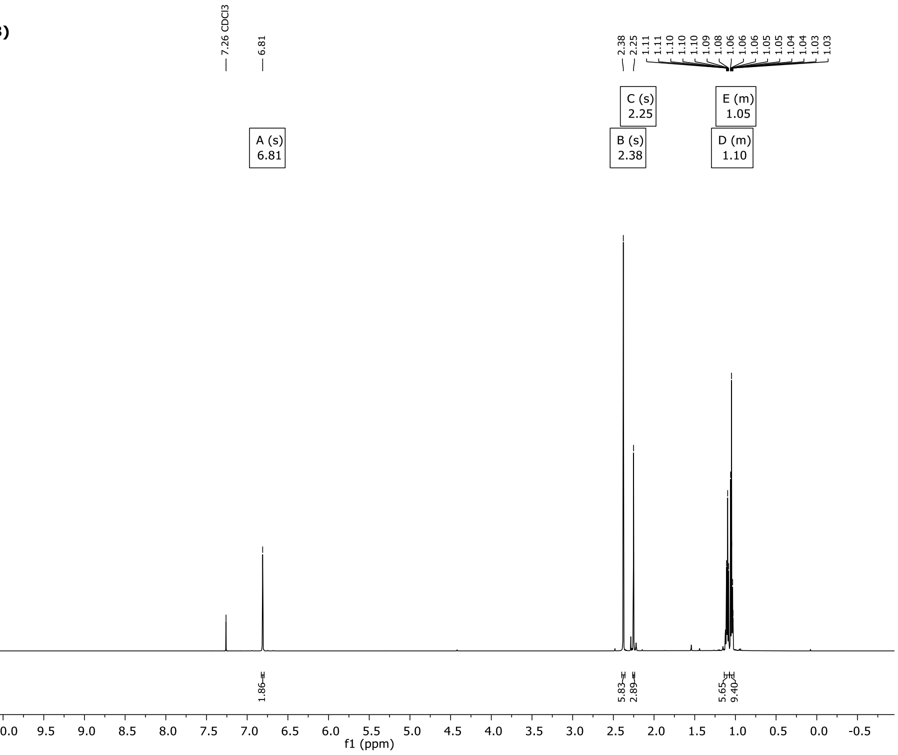

13C NMR
(150.85 MHz, CDCl3)

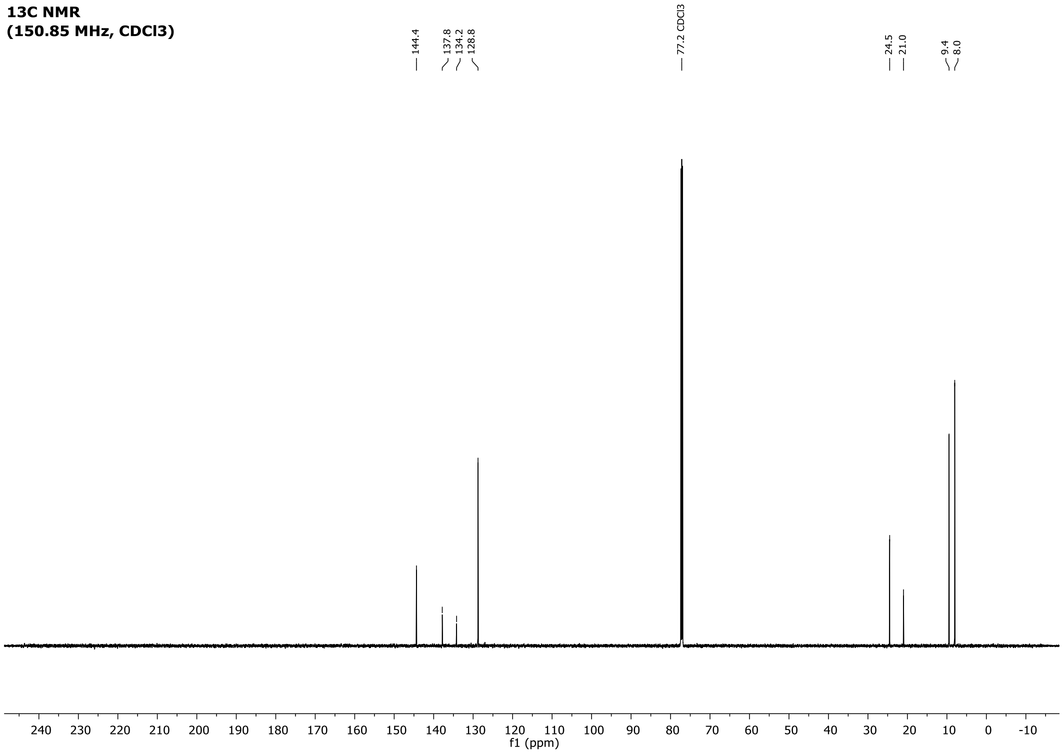

Triethyl(4-chlorophenyl)germane

1H NMR
(599.86 MHz, CDCl3)

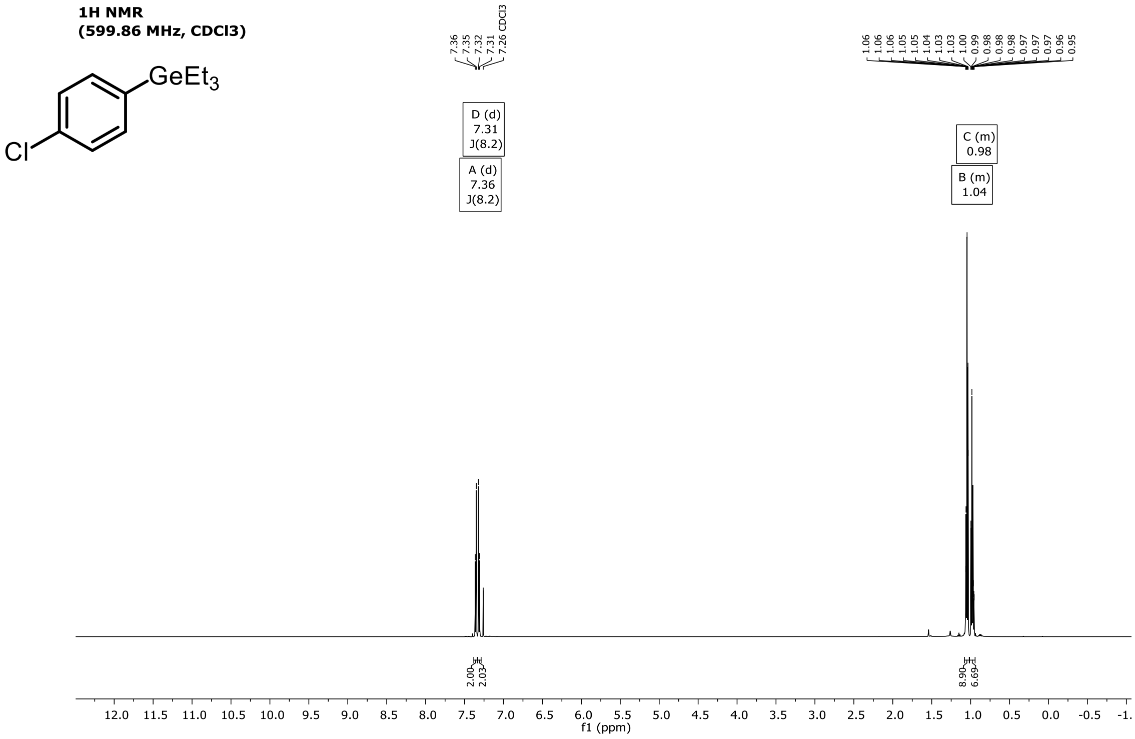

13C NMR
(150.85 MHz, CDCl3)

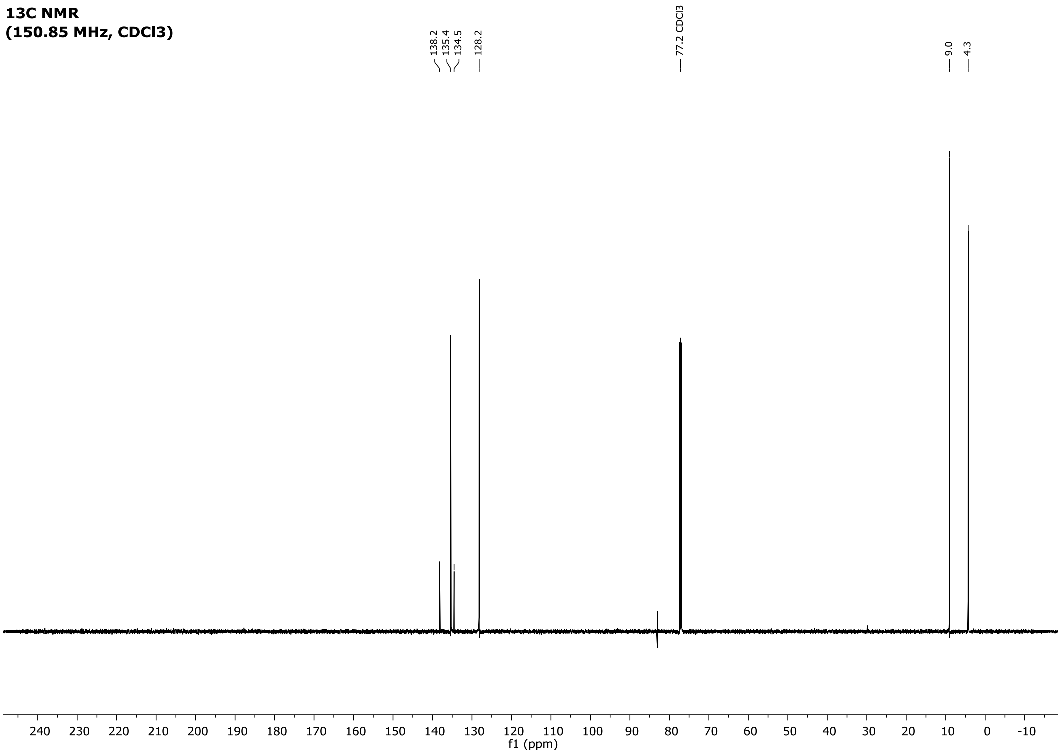

Triethyl(4-bromophenyl)germane

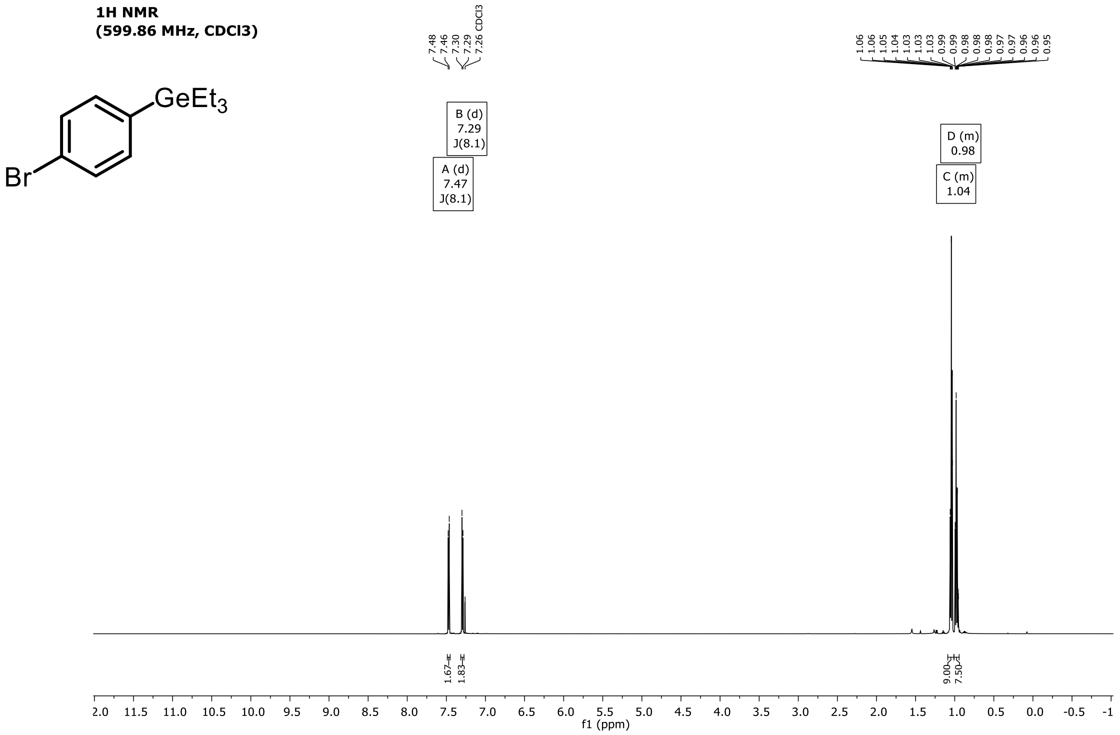

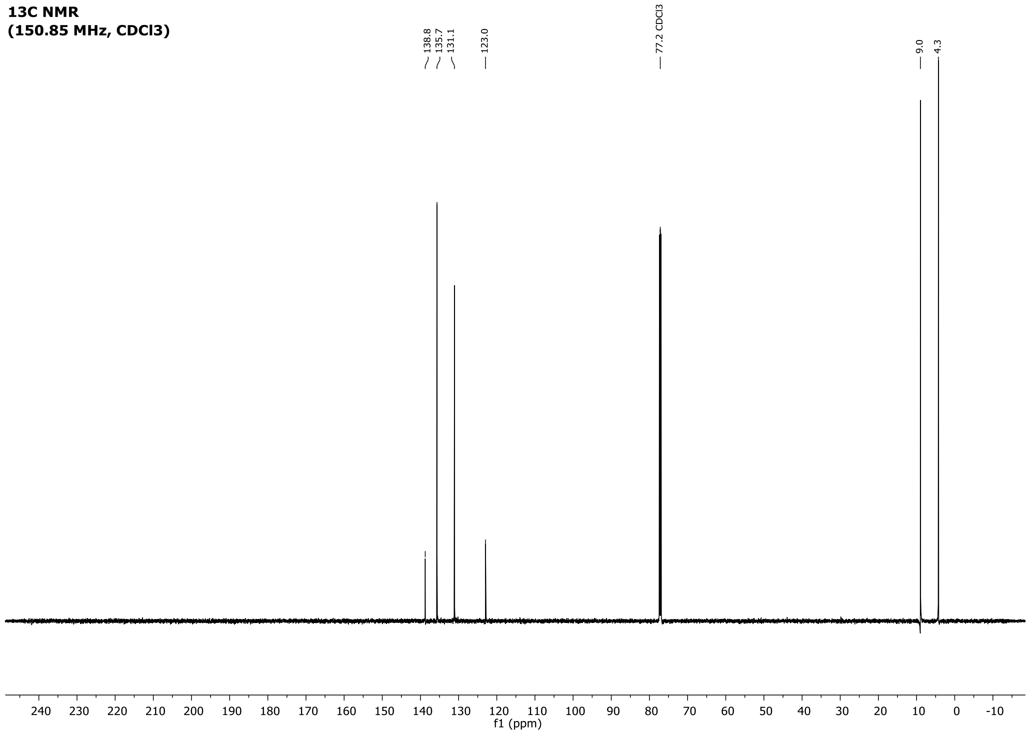

Triethyl(4-iodophenyl)germane

1H NMR
(599.86 MHz, CDCl3)

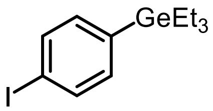

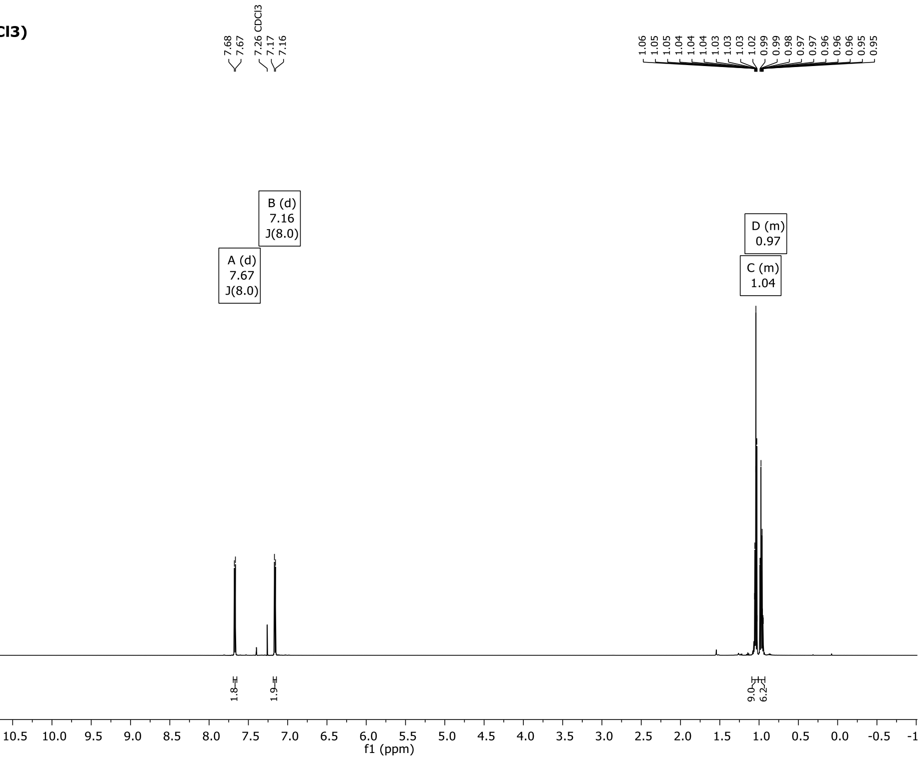

13C NMR
(150.85 MHz, CDCl3)

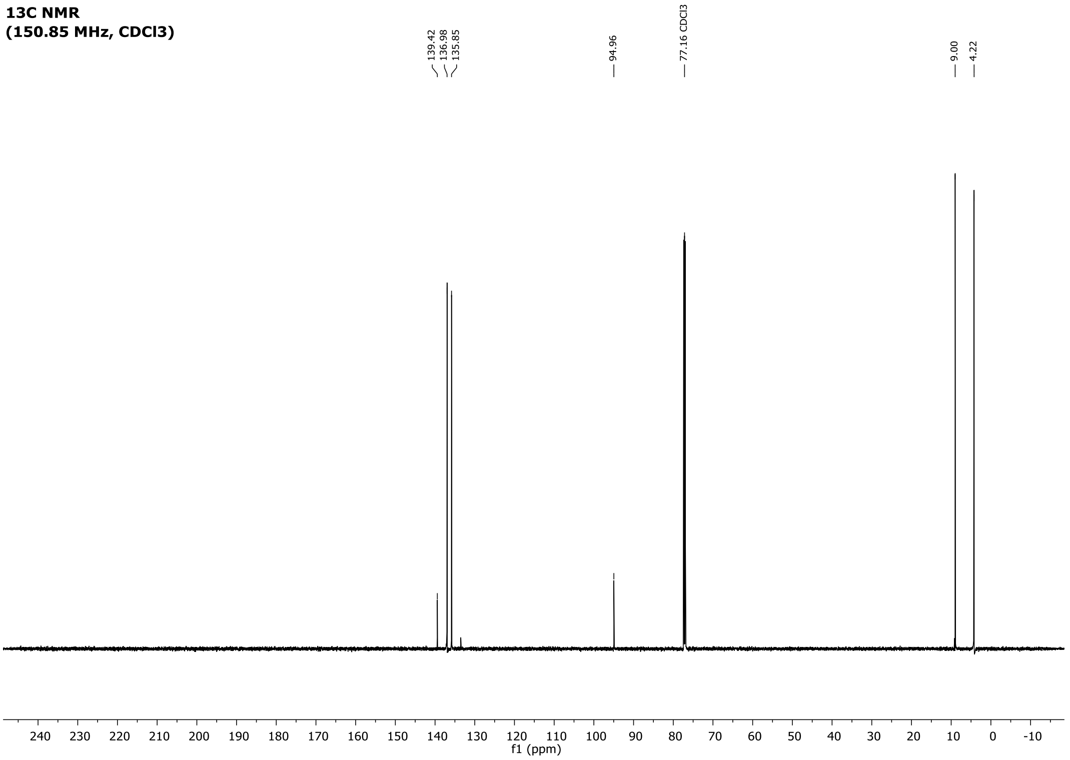

Triethyl(thiophen-2-yl)germane

1H NMR
(599.86 MHz, CDCl3)

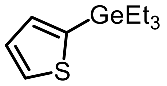

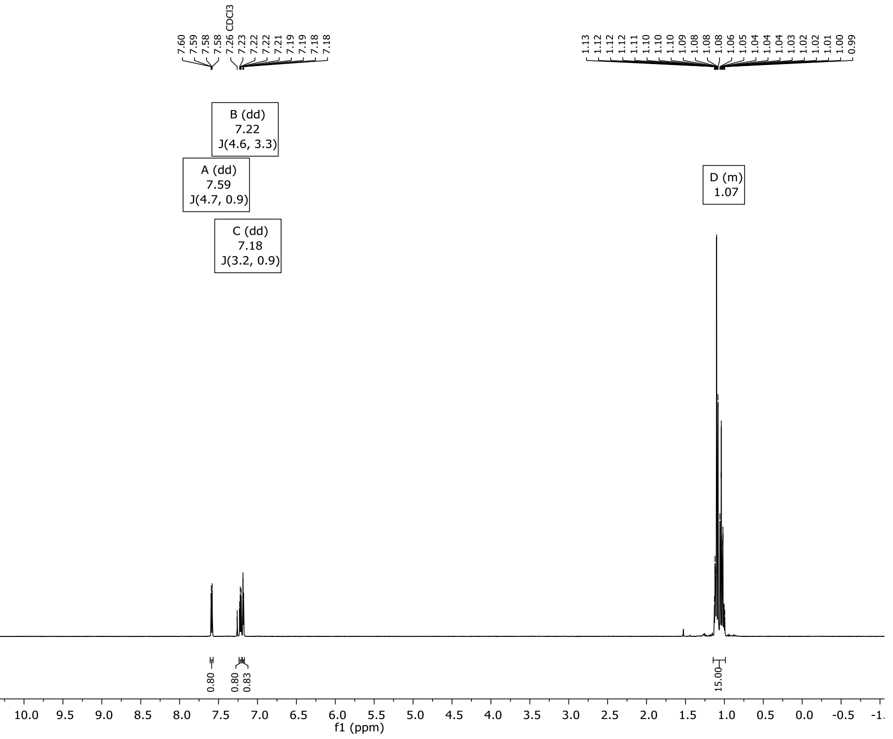

13C NMR
(150.85 MHz, CDCl3)

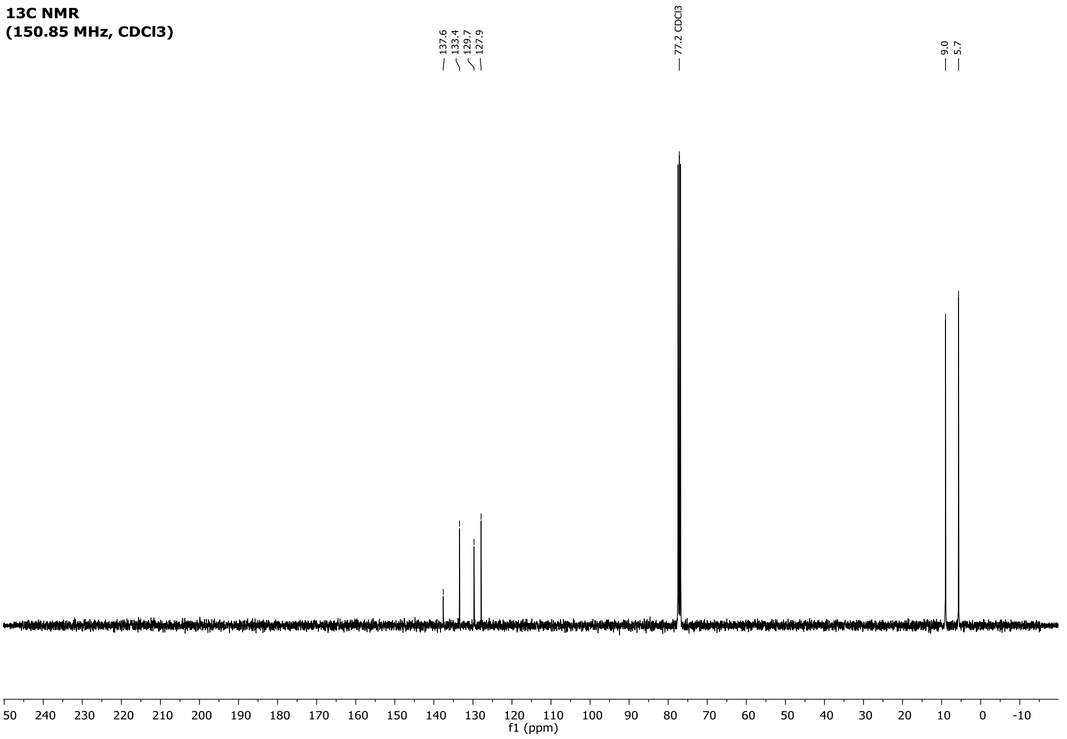

Triethyl(*p*-tolyl)germane

1H NMR
(599.86 MHz, CDCl3)

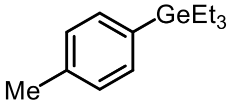

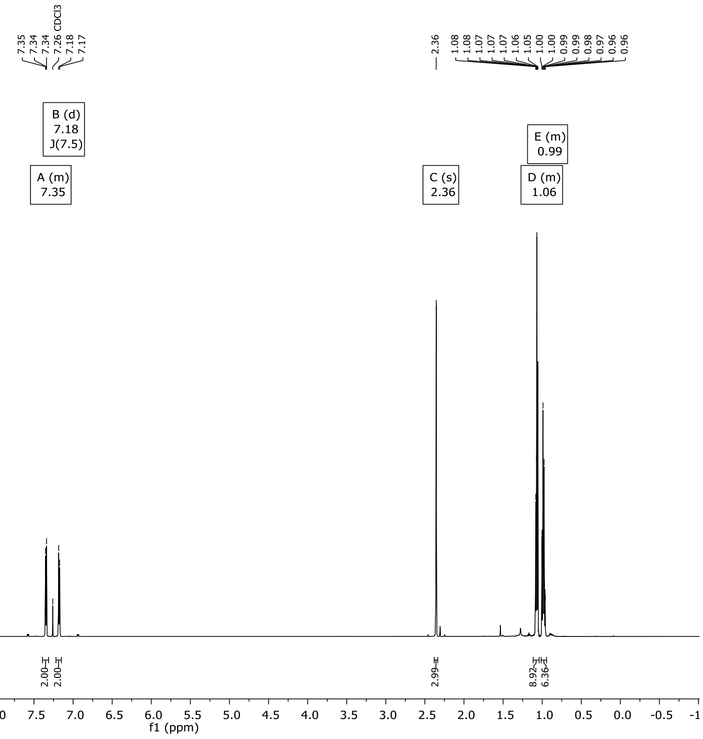

13C NMR
(150.85 MHz, CDCl3)

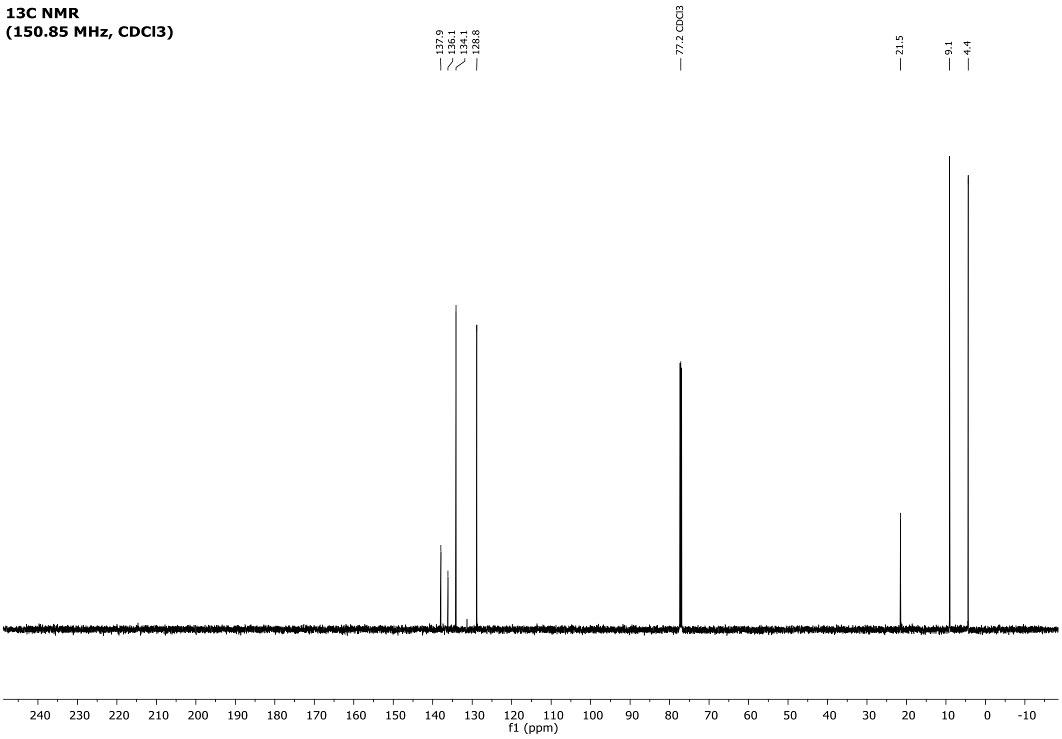

Triethyl(4-(4,4,5,5-tetramethyl-1,3,2-dioxaborolan-2-yl)phenyl)germane

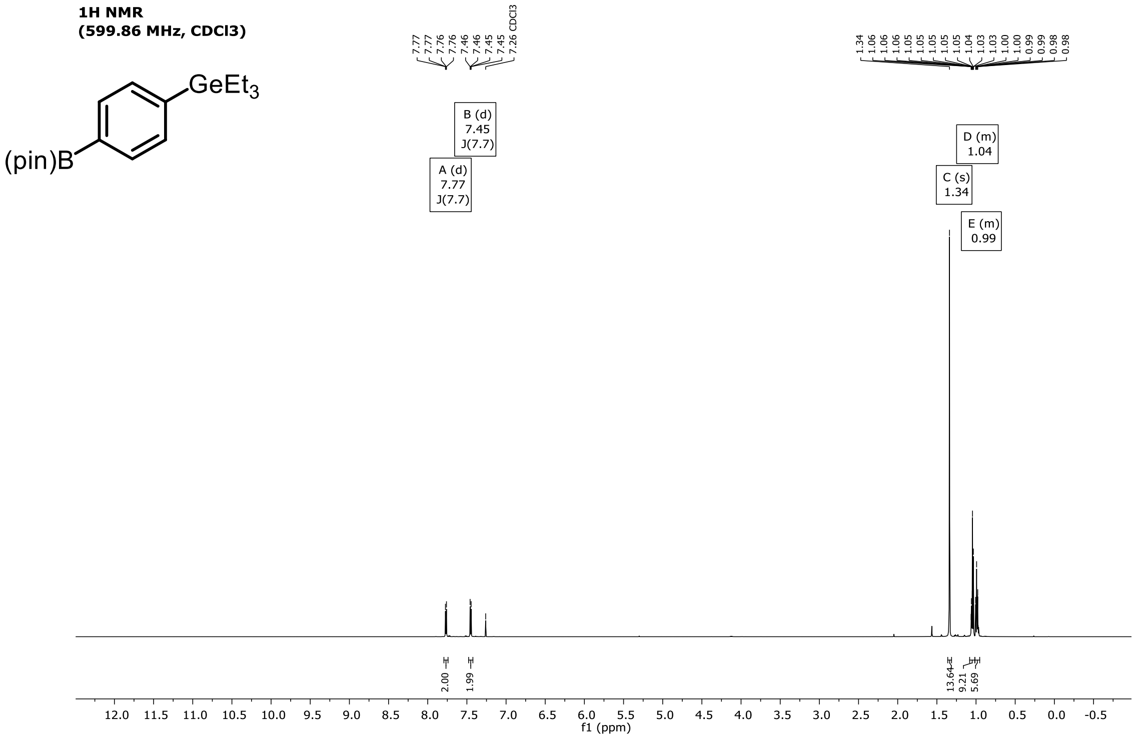

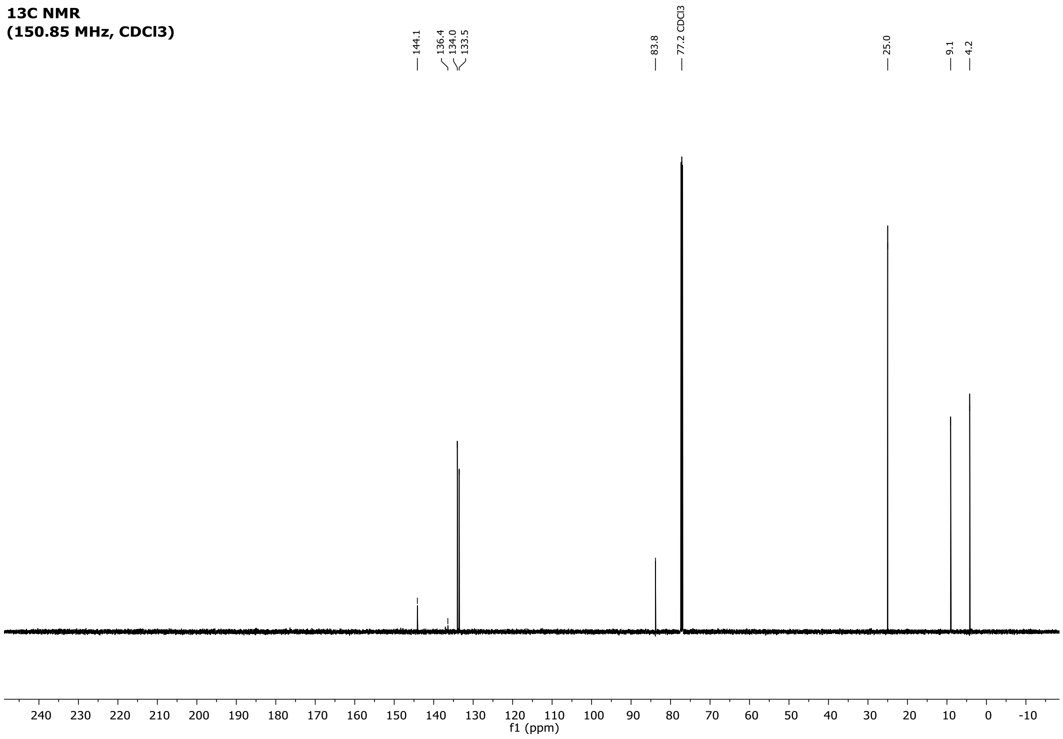

Triethyl(4-(tributylstannyl)phenyl)germane

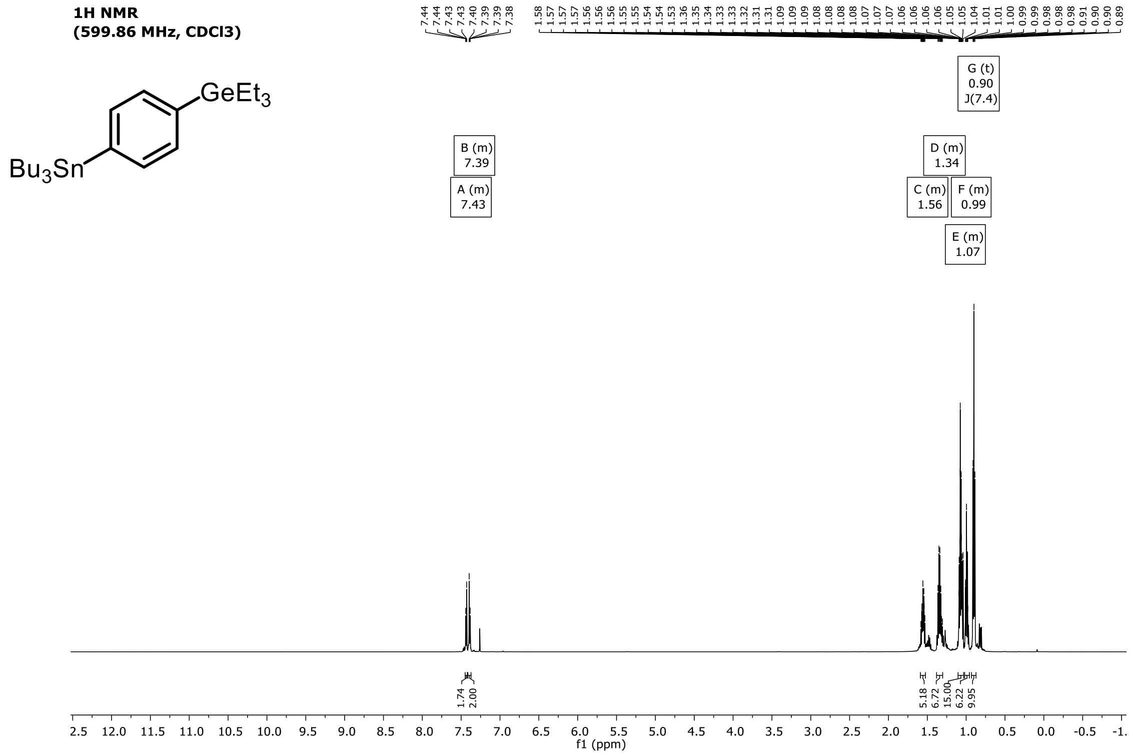

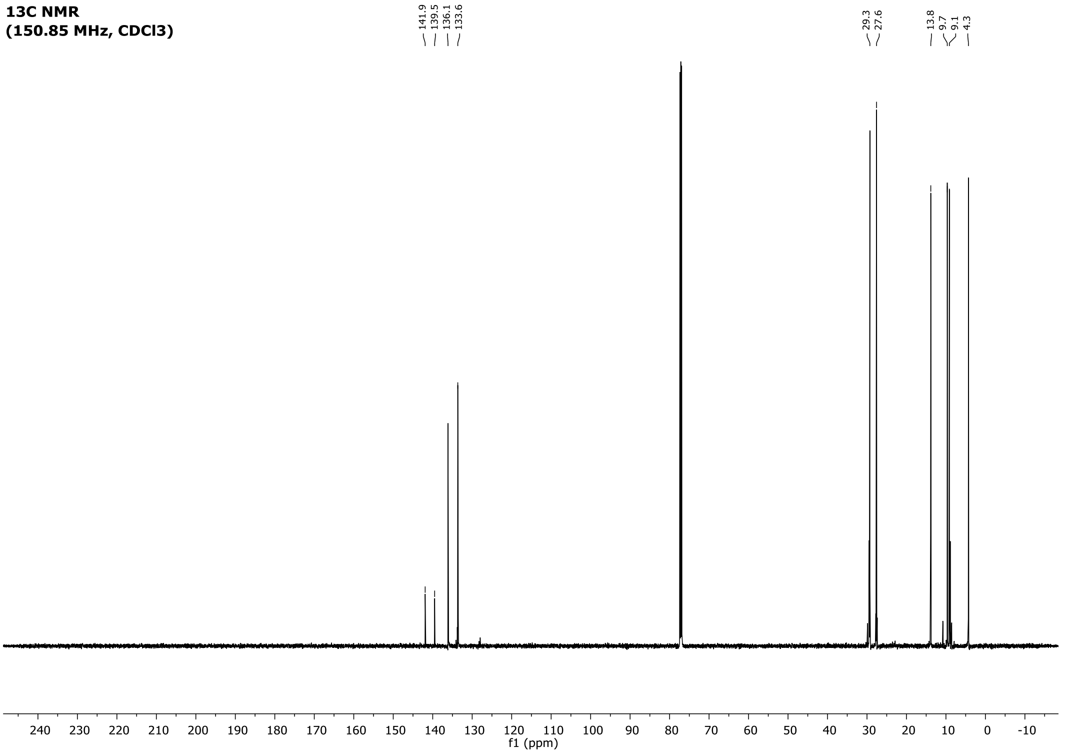

Triethyl(thiophen-3-yl)germane

1H NMR
(599.86 MHz, CDCl3)

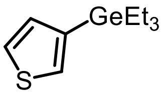

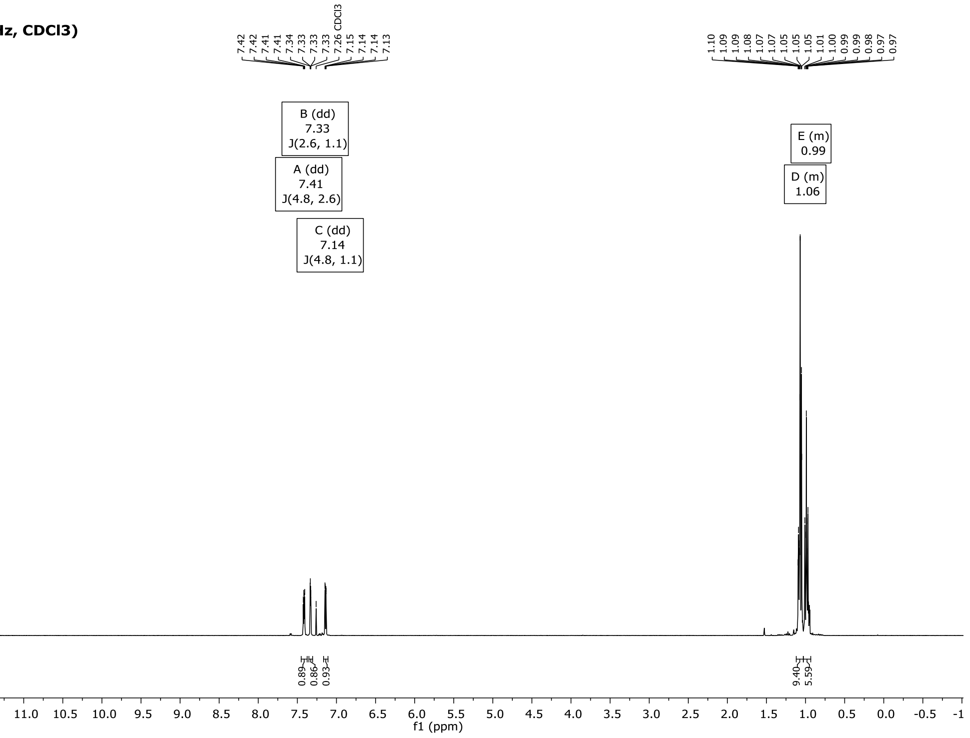

13C NMR
(150.85 MHz, CDCl3)

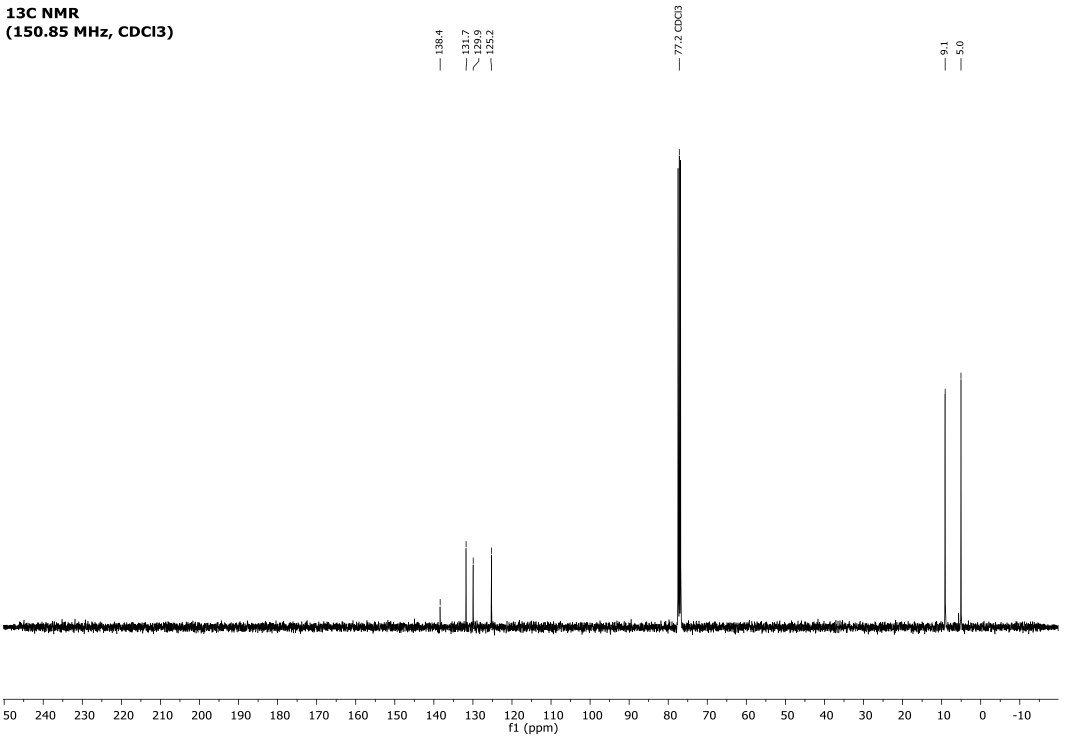

Benzo[b]thiophen-3-yltriethylgermane

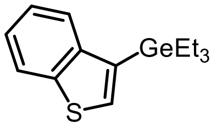

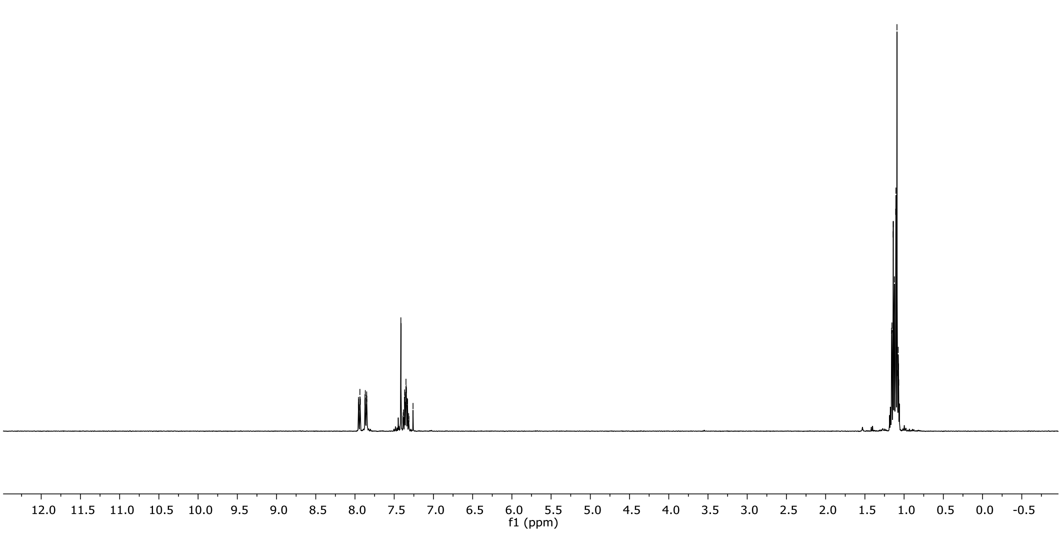

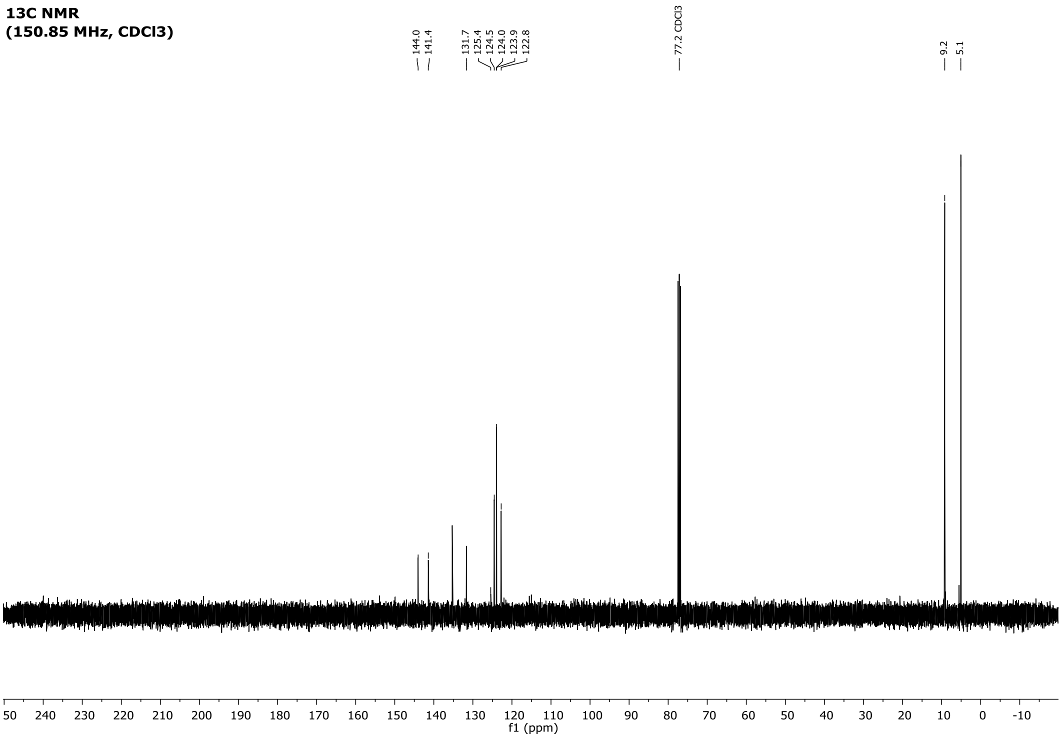

Triethyl(naphthalen-1-yl)germane

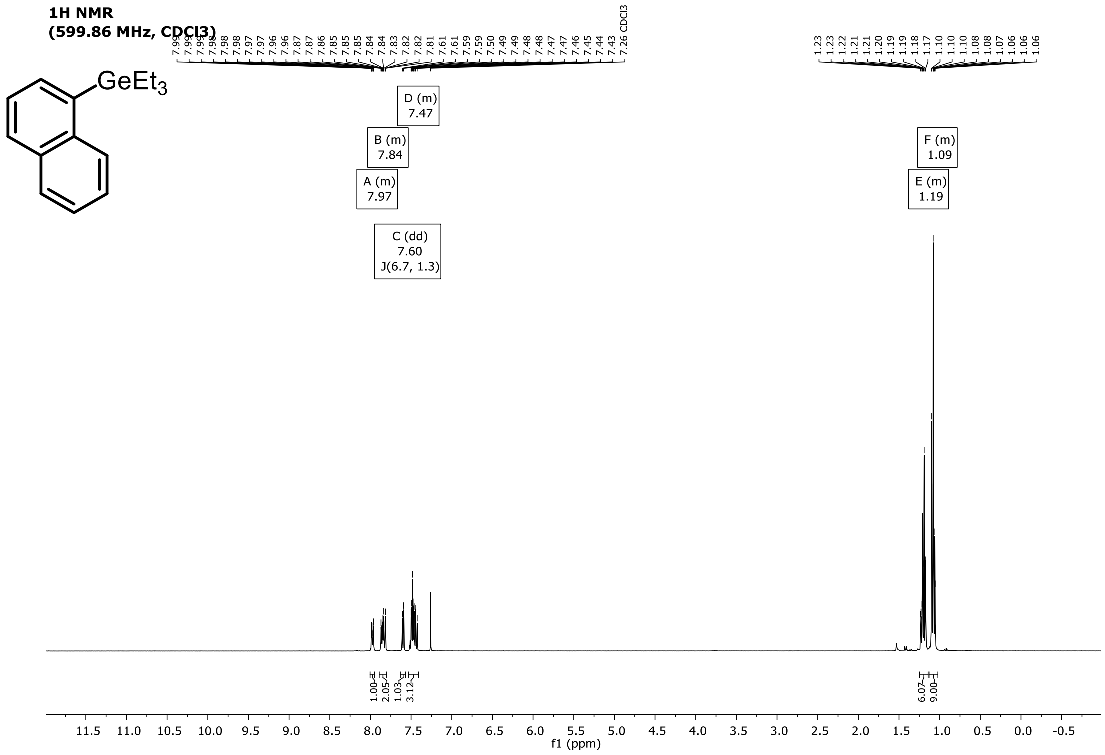

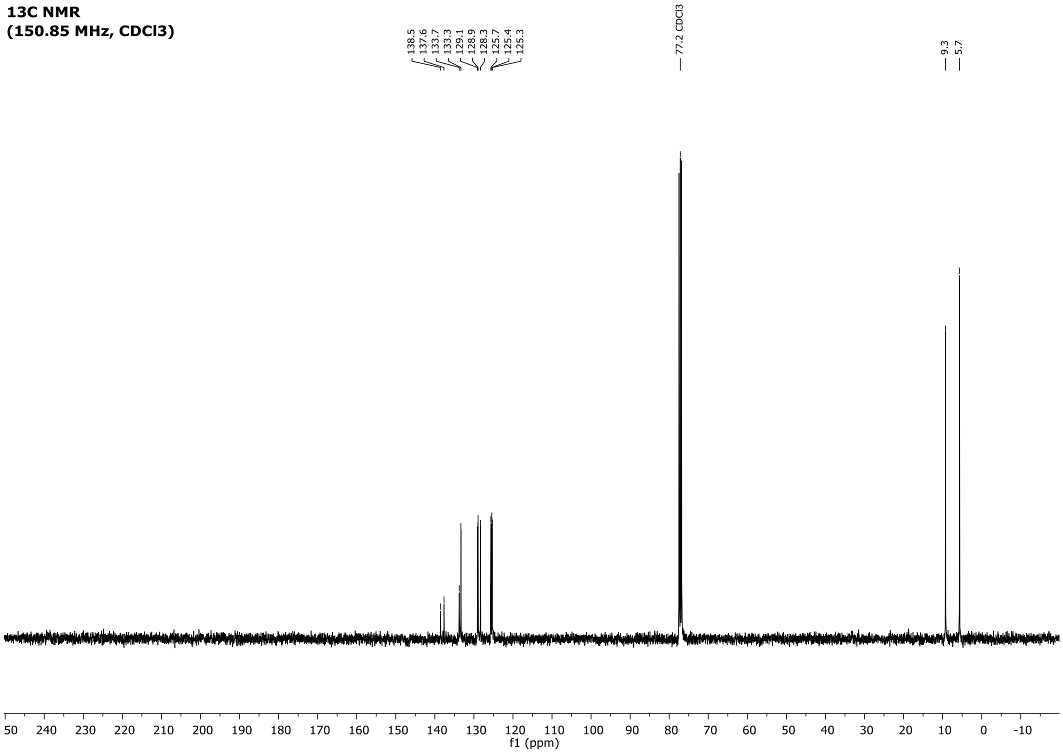

(2,6-Dimethoxyphenyl)triethylgermane

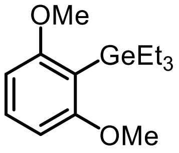

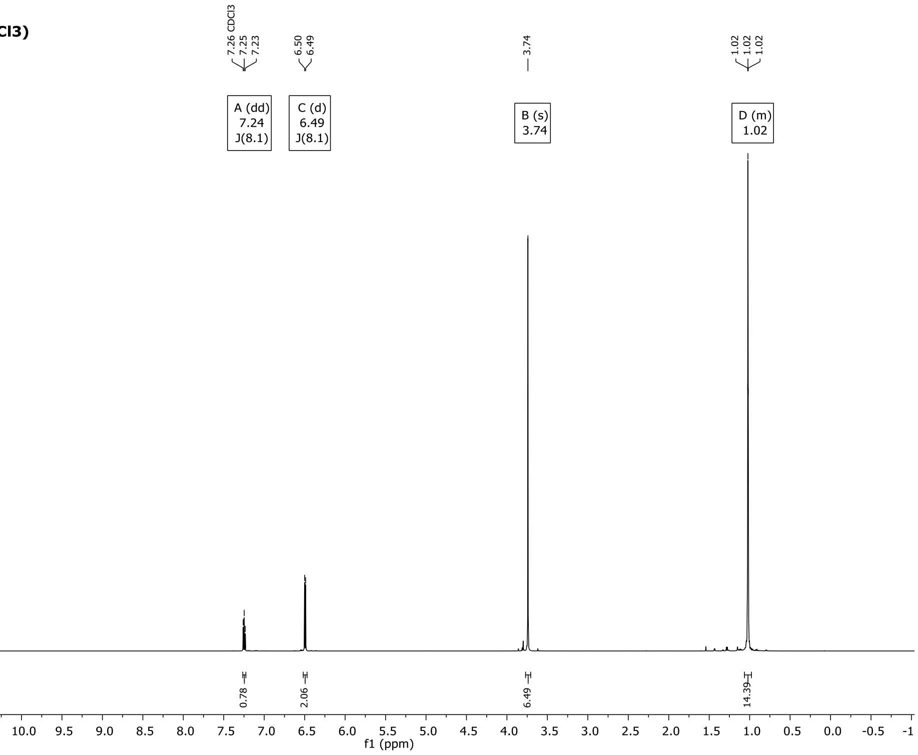

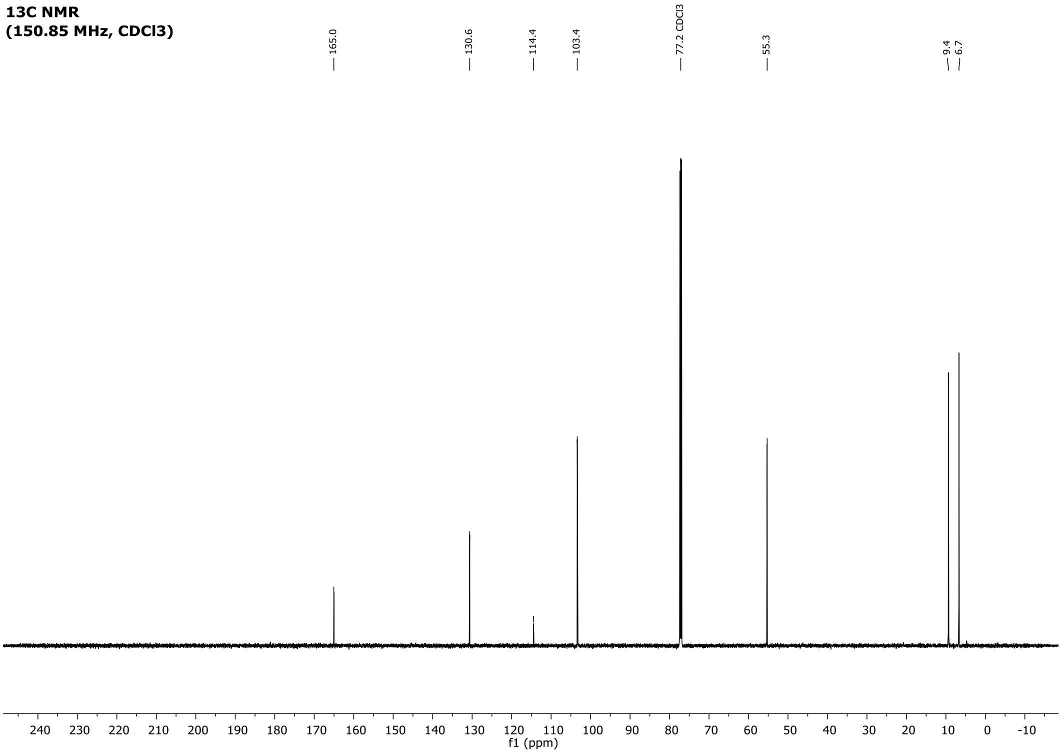

Triethyl(3,4,5-trimethoxyphenyl)germane

1H NMR
(599.86 MHz, CDCl3)

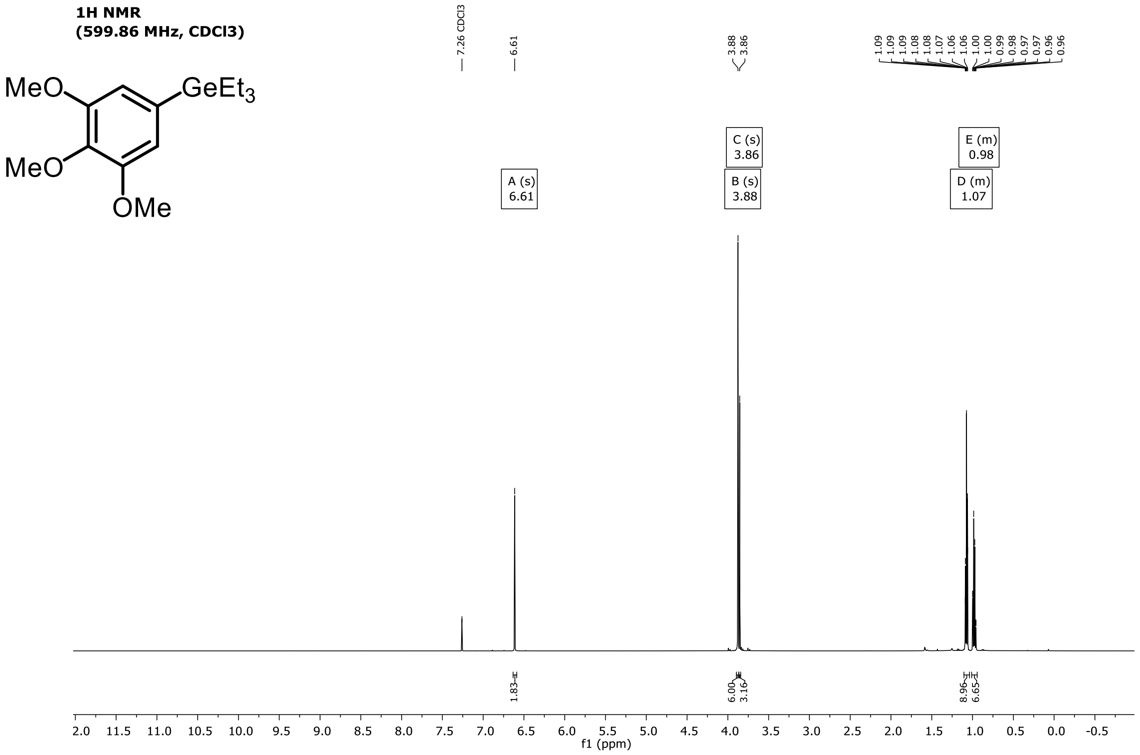

13C NMR
(150.85 MHz, CDCl3)

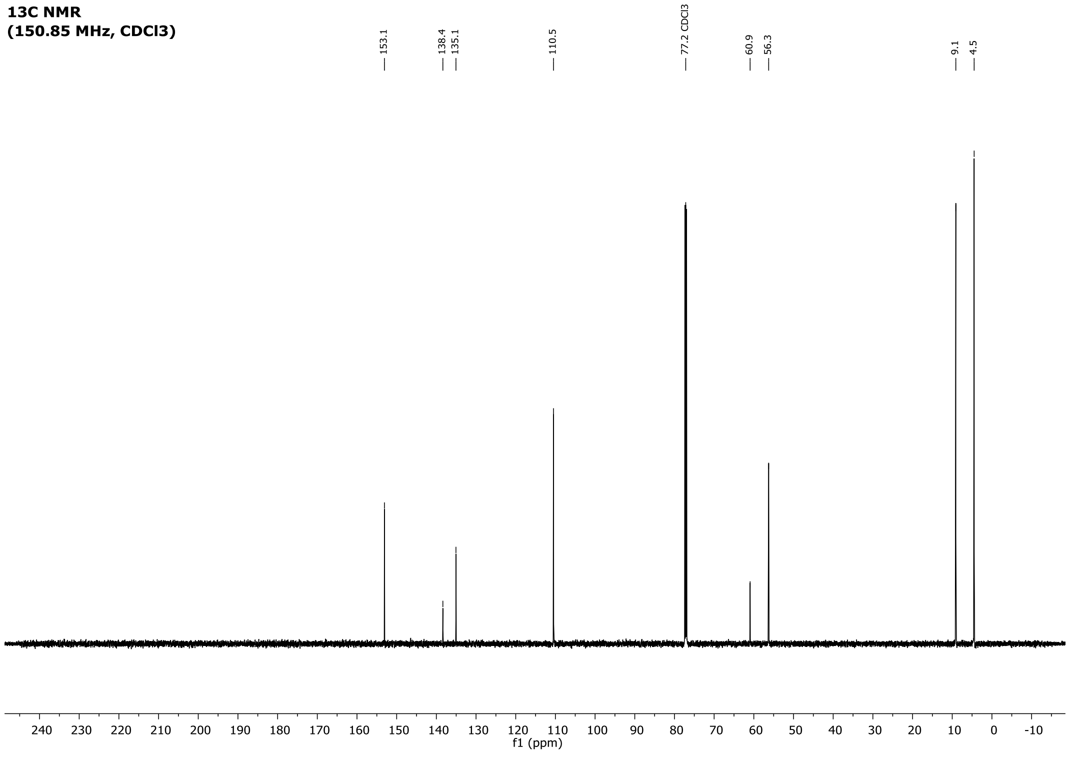

Triethyl(2-methoxyphenyl)germane

1H NMR
(599.86 MHz, CDCl3)

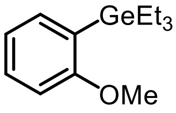

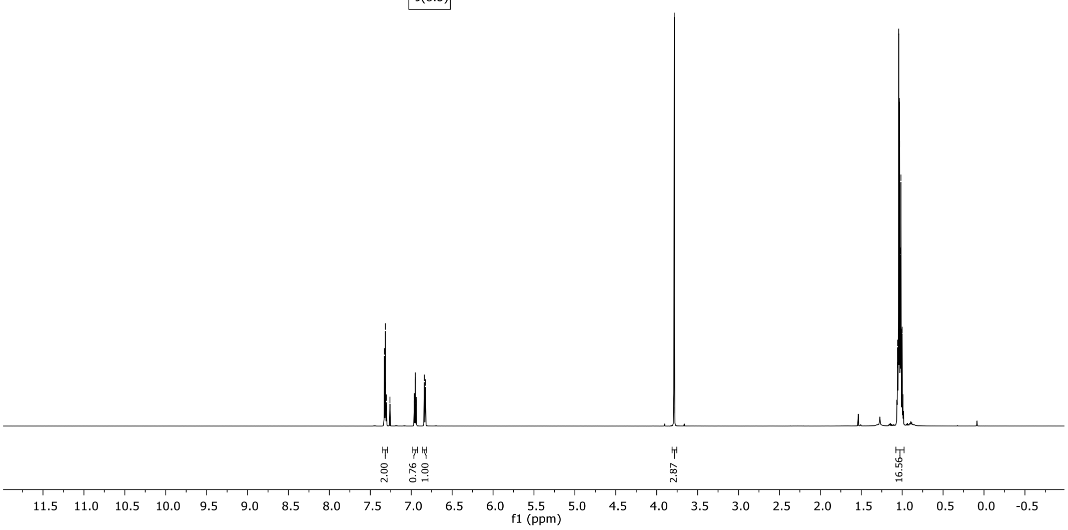

13C NMR
(150.85 MHz, CDCl3)

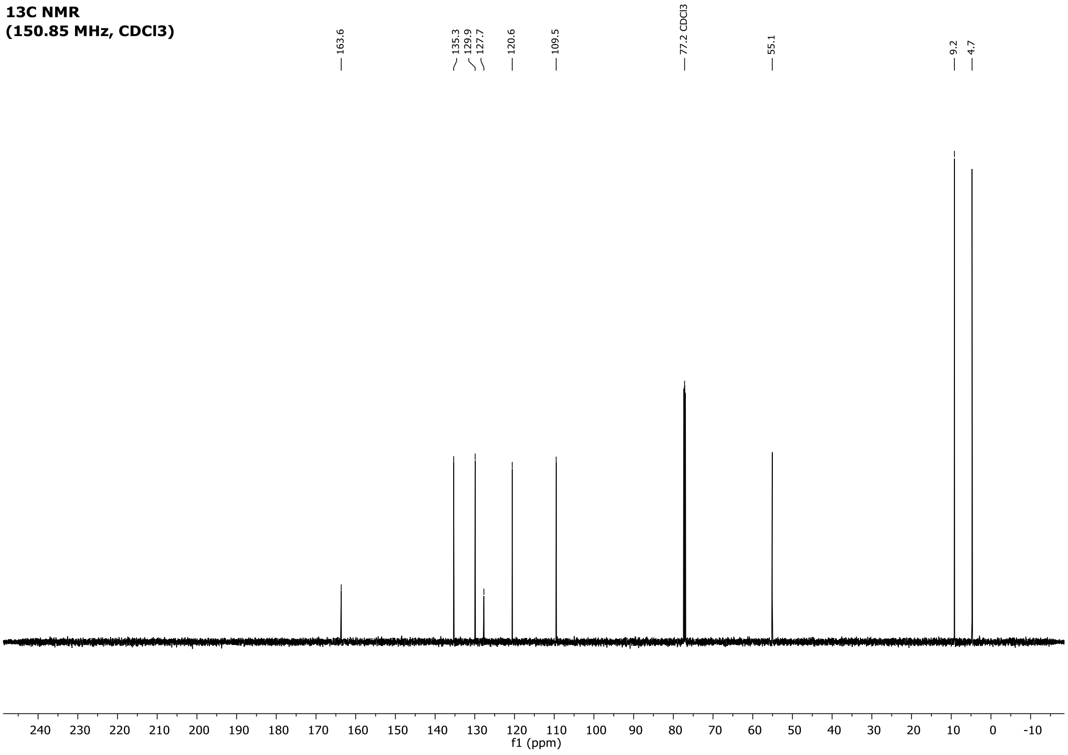

Trimethyl(4-(triethylgermyl)phenyl)silane

1H NMR
(599.86 MHz, CDCl3)

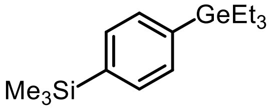

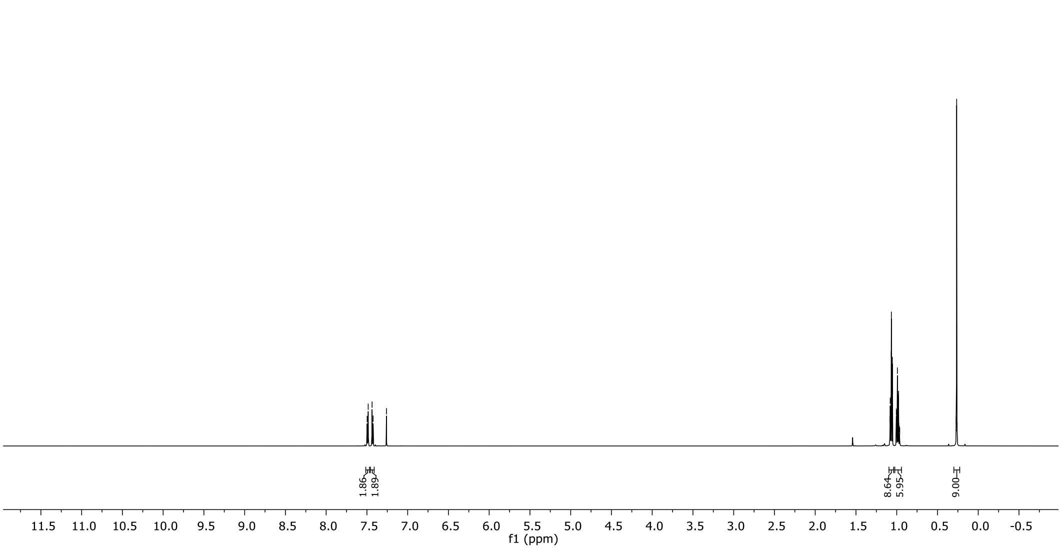

13C NMR
(150.85 MHz, CDCl3)

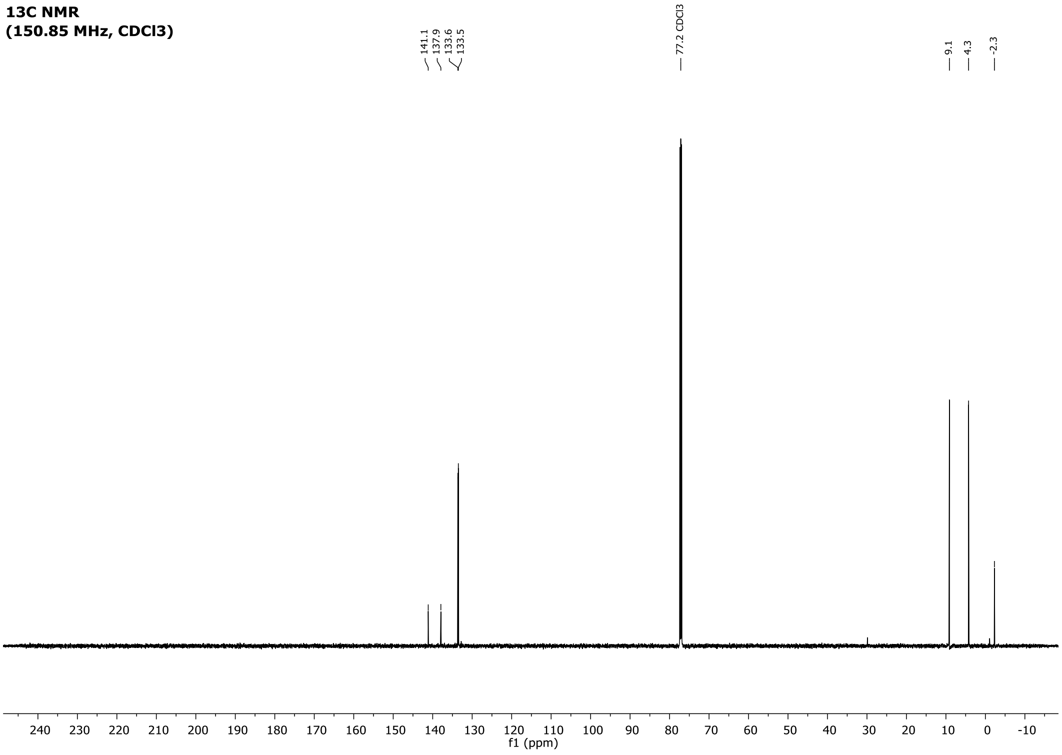

3,5-Dimethyl-4-(triethylgermyl)isoxazole

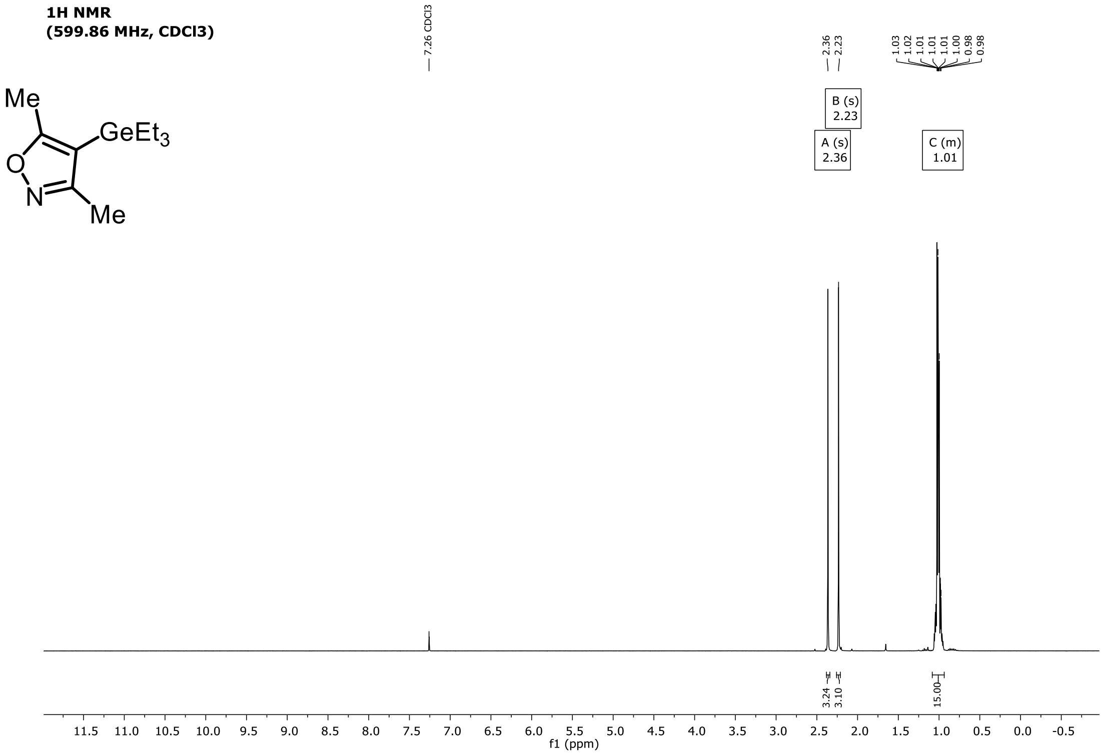

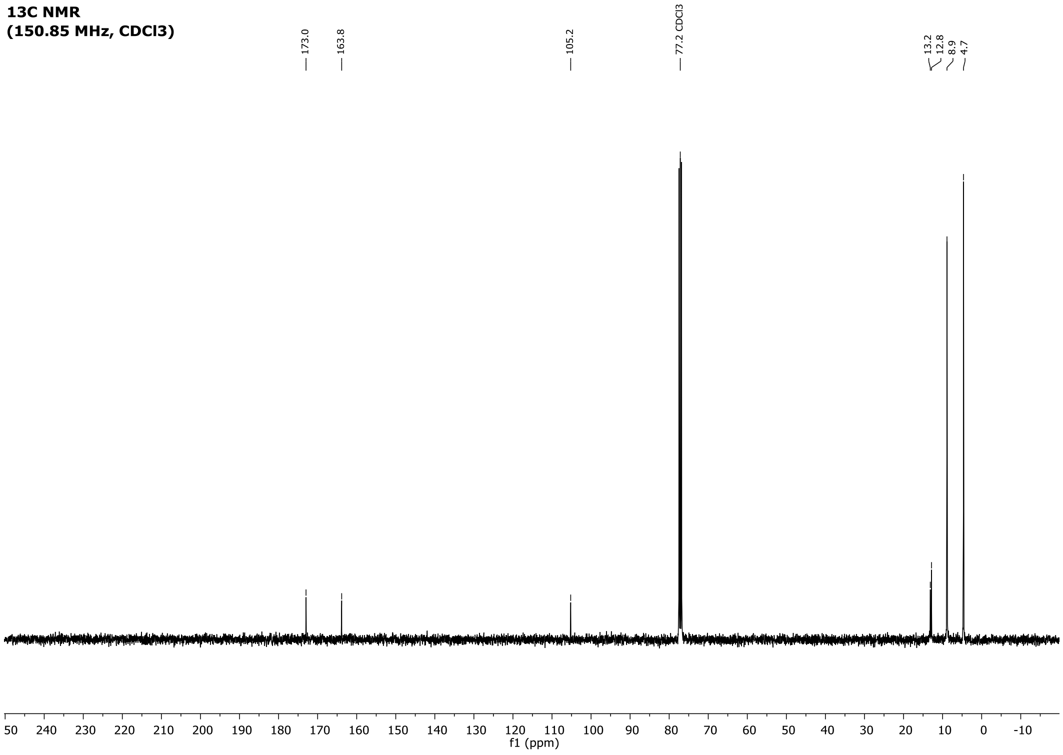

***tert*-Butyl{(Z)-[(3-iodobenzyl)amino][(*tert*-butoxycarbonyl)amino]methylidene} carbamate**

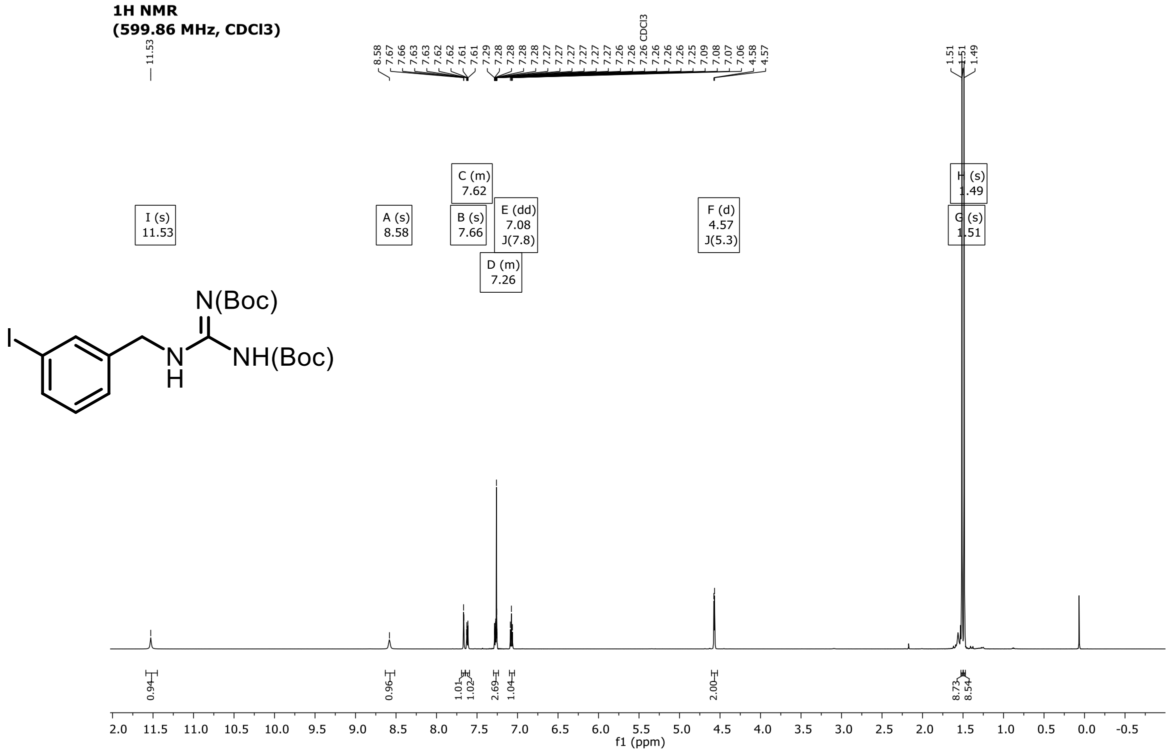

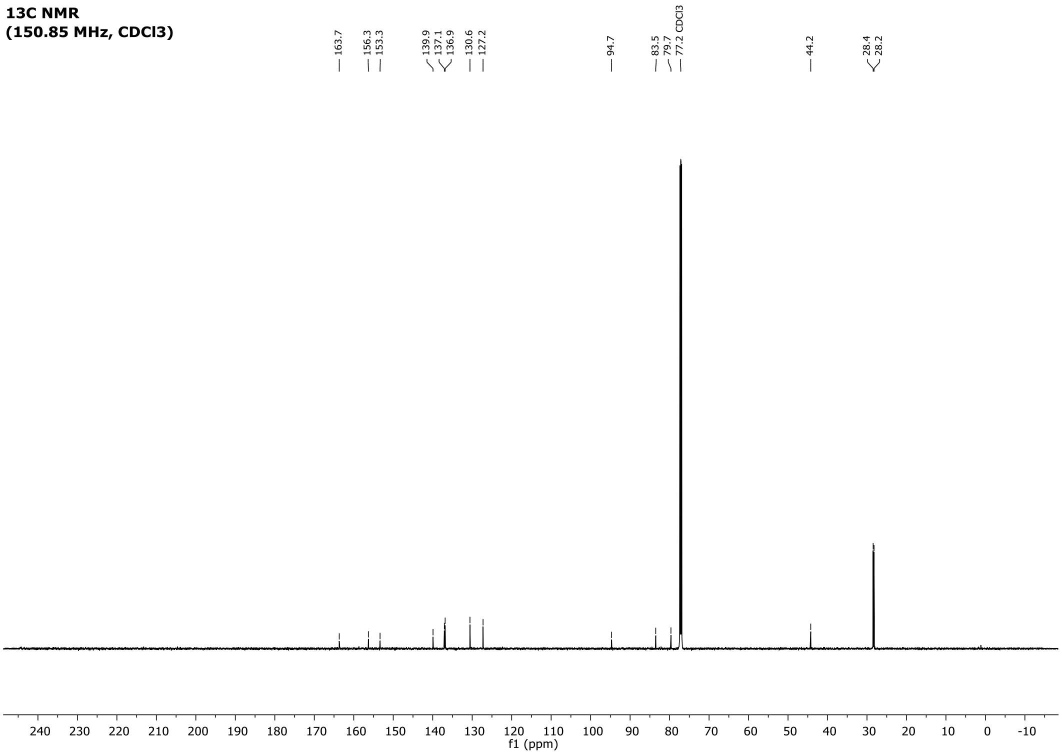

tert-Butyl-N-[(1Z)-{bis[(*tert*-butoxy)carbonyl]amino}({[(*tert*-butoxy)carbonyl][(3-iodophenyl) methyl]amino})methylidene]carbamate

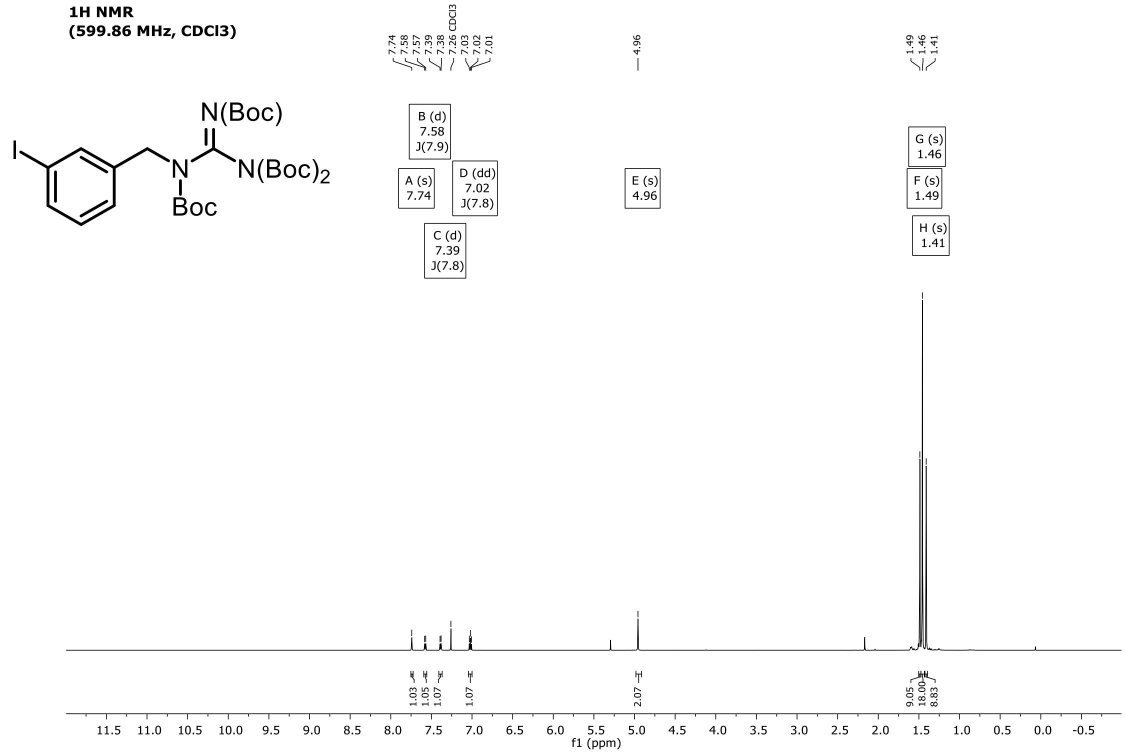

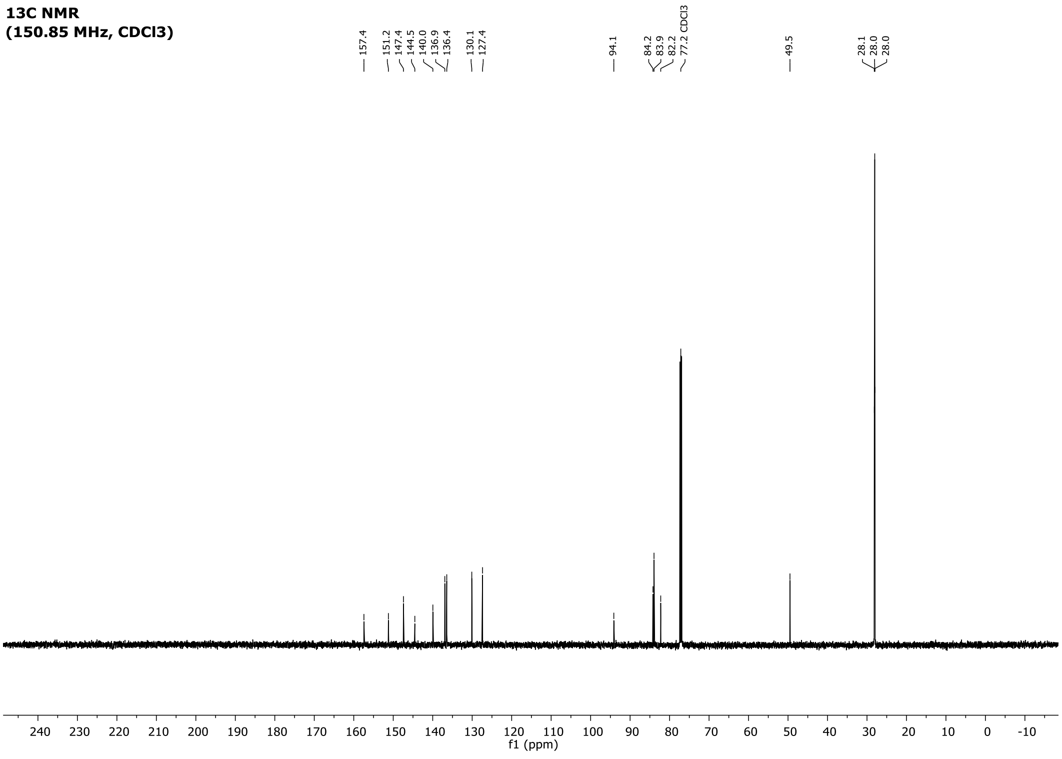

tert-Butyl-N-[(1Z)-{[(*tert*-butoxy)carbonyl]amino}({[(*tert*-butoxy)carbonyl][(3-(triethylgermanium)phenyl) methyl]amino})methylidene]carbamate

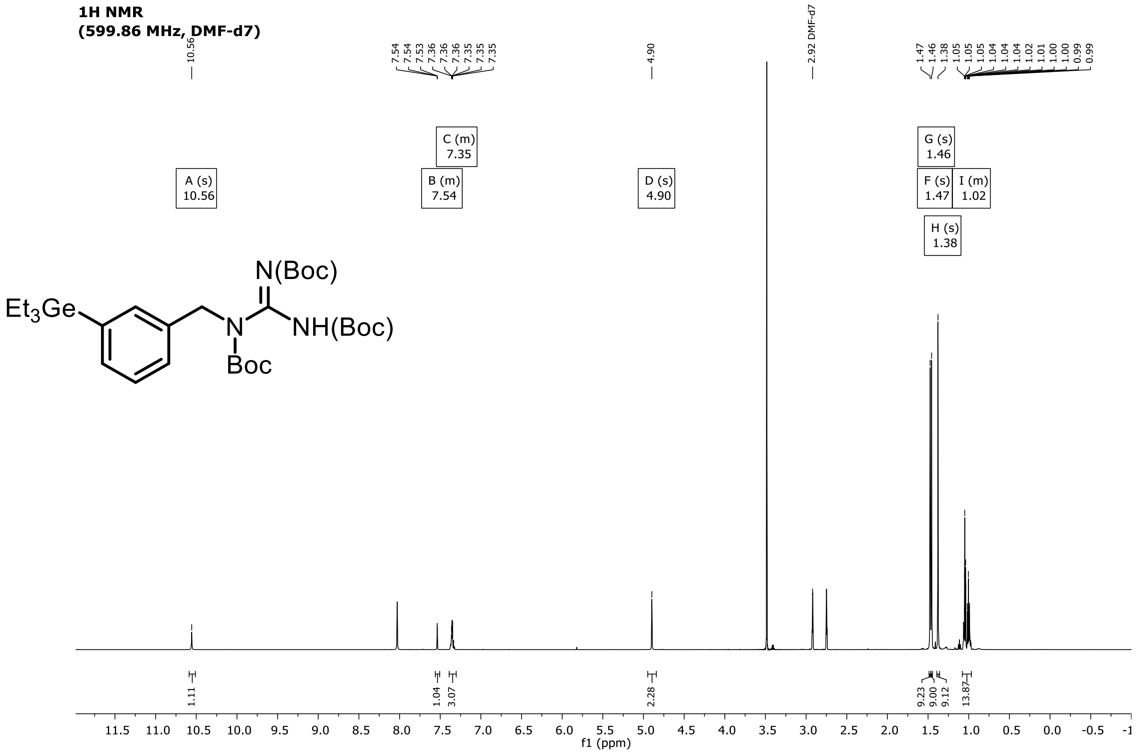

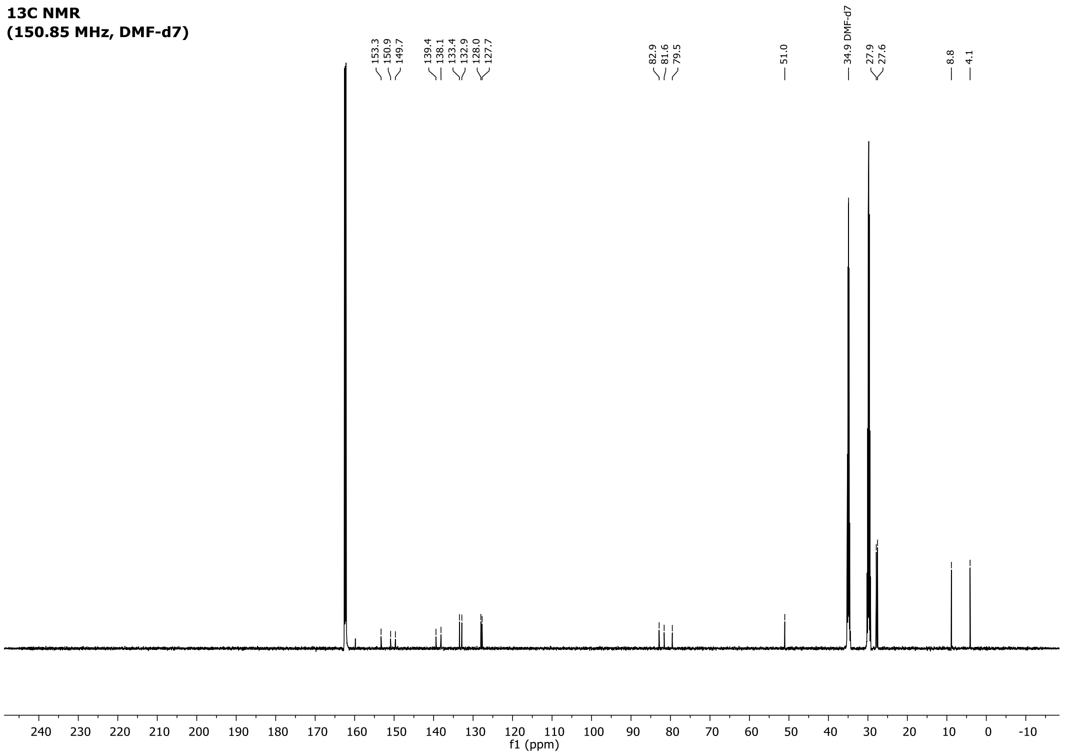

***tert*-Butyl{(Z)-[(3-bromobenzyl)amino][(*tert*-butoxycarbonyl)amino]methylidene} carbamate**

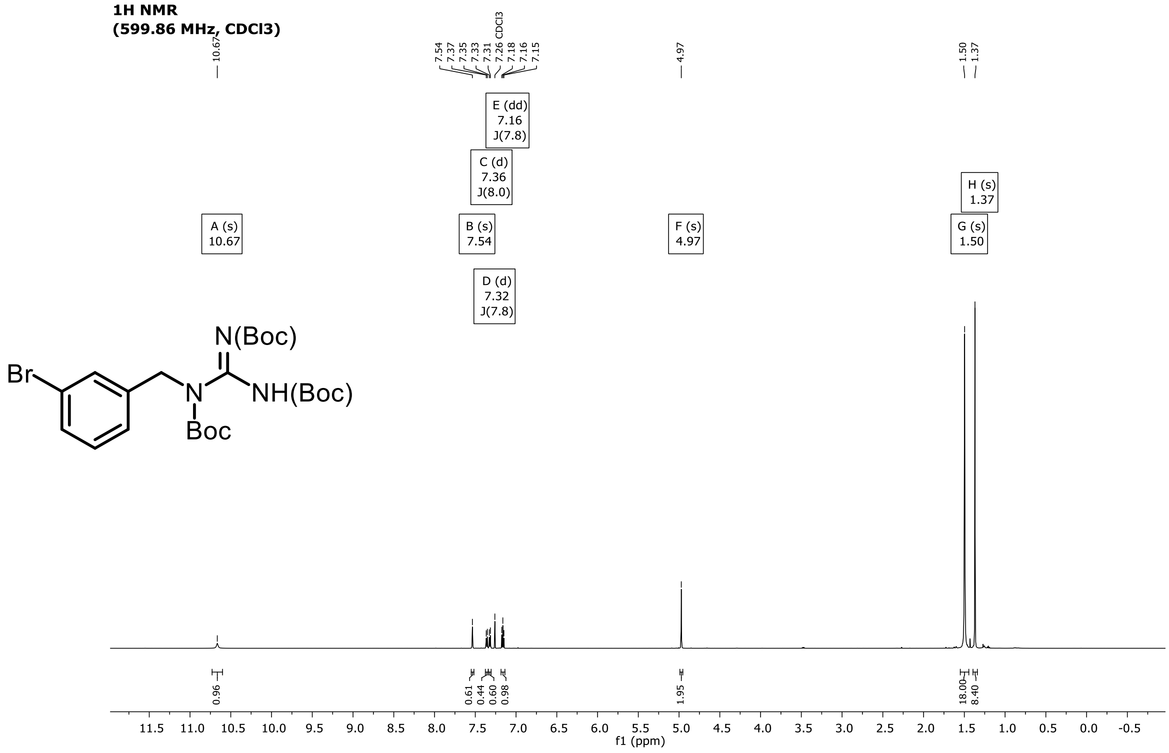

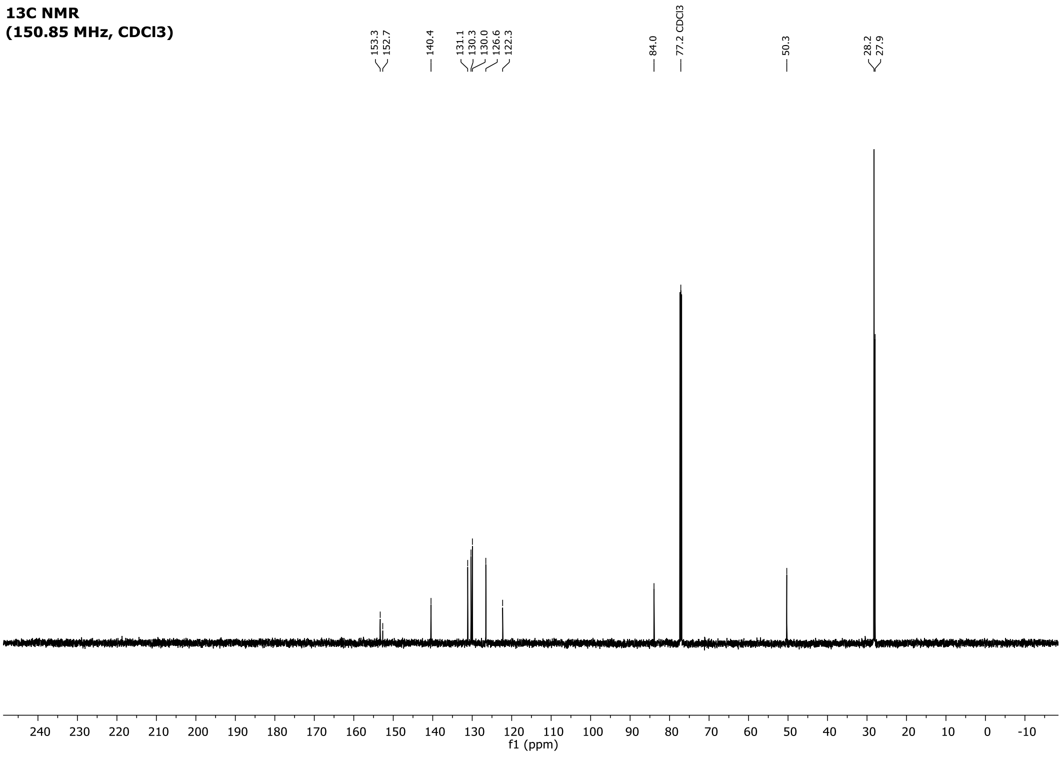

tert-Butyl-N-[(1Z)-{bis- [(_tert_-butoxy) carbonyl] amino} ({[(_tert_-butoxy) carbonyl] [(3-(triethylgermanium)-phenyl) methyl]amino})methylidene]carbamate

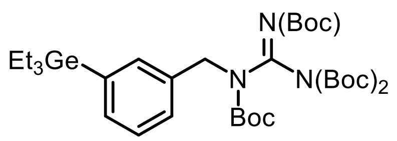

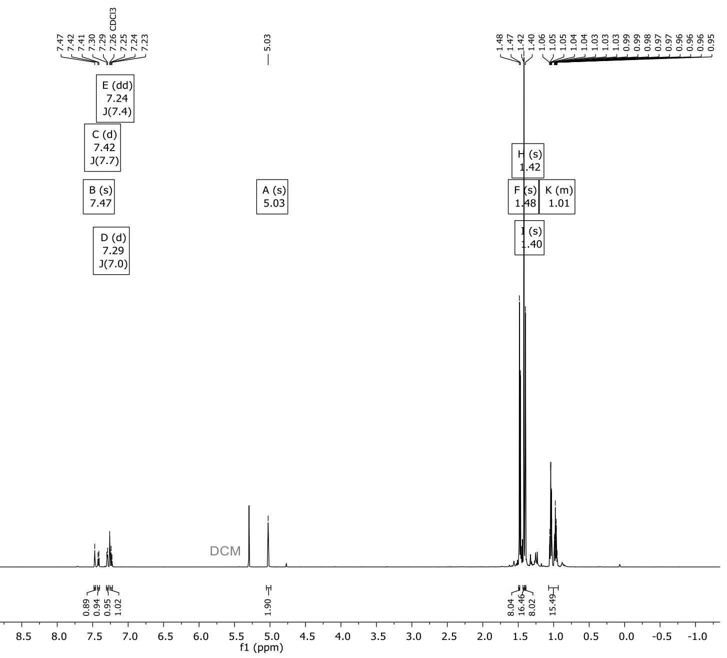

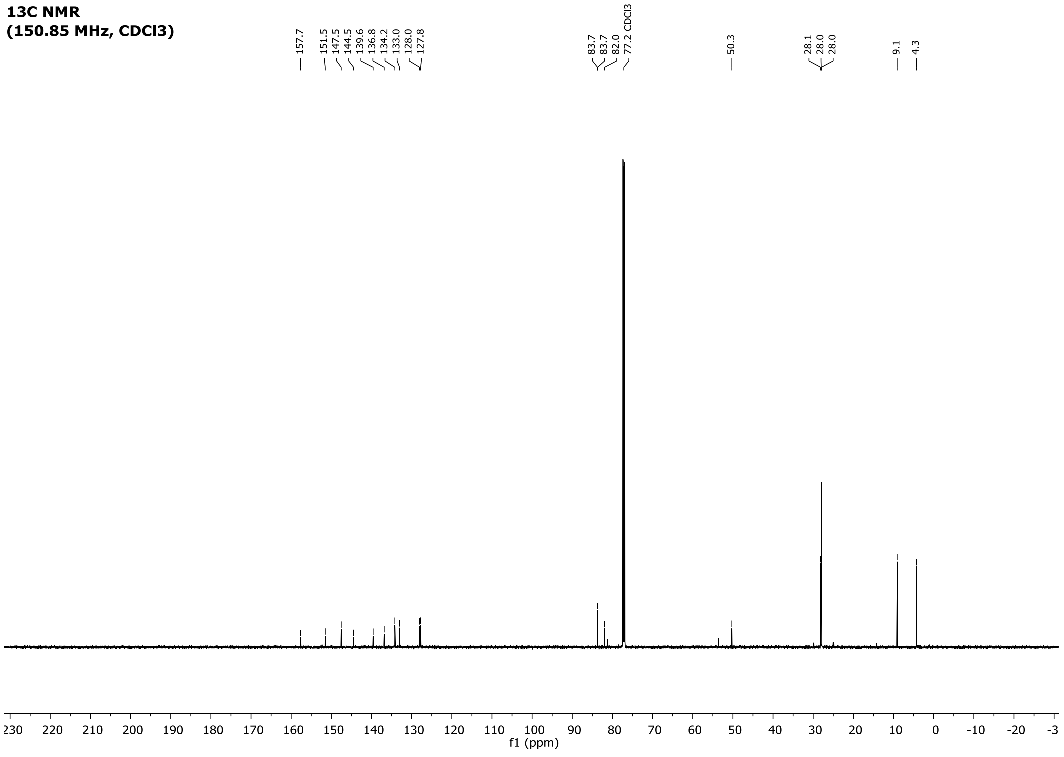

References

[1] V. Percec, M. N. Holerca, S. Nummelin, J. J. Morrison, M. Glodde, J. Smidrkal, M. Peterca, B. M. Rosen, S. Uchida, V. S. Balagurusamy, M. J. Sienkowska, P. A. Heiney, *Chem. Eur. J.* **2006**, *12*, 6216-6241.

[2] S. Orbisaglia, B. Jacques, P. Braunstein, D. Hueber, P. Pale, A. Blanc, P. de Fremont, *Organometallics* **2013**, *32*, 4153-4164.

[3] R. J. Tang, T. Milcent, B. Crousse, *J. Org. Chem.* **2018**, *83*, 930-938.

[4] V. P. Singh, J. Yan, J. F. Poon, P. J. Gates, R. J. Butcher, L. Engman, *Chem. Eur. J.* **2017**, *23*, 15080-15088.

[5] Z. Fu, Z. Li, Y. Song, R. Yang, Y. Liu, H. Cai, *J. Org. Chem.* **2016**, *81*, 2794-2803.

[6] M. Bergstrom, G. Suresh, V. R. Naidu, C. R. Unelius, *Eur. J. Org. Chem.* **2017**, *2017*, 3234-3239.

[7] S. Mukhopadhyay, S. Batra, *Chem. Eur. J.* **2018**, *24*, 14622-14626.

[8] S. Fredrich, A. Bonasera, V. Valderrey, S. Hecht, *J. Am. Chem. Soc.* **2018**, *140*, 6432-6440.

[9] M. Chen, S. Ichikawa, S. L. Buchwald, *Angew. Chem. Int. Ed.* **2015**, *54*, 263-266.

[10] G. J. P. Perry, J. M. Quibell, A. Panigrahi, I. Larrosa, *J. Am. Chem. Soc.* **2017**, *139*, 11527-11536.

[11] H. Fu, M. Jiang, H. Yang, Y. Jin, L. Ou, *Synlett* **2018**, *29*, 1572-1577.

[12] C. Fricke, G. J. Sherborne, I. Funes-Ardoiz, E. Senol, S. Guven, F. Schoenebeck, *Angew. Chem. Int. Ed.* **2019**, *58*, 17788-17795.

[13] C. Fricke, A. Dahiya, W. B. Reid, F. Schoenebeck, *ACS Catal.* **2019**, *9*, 9231-9236.

[14] A. Dahiya, C. Fricke, F. Schoenebeck, *J. Am. Chem. Soc.* **2020**, *142*, 7754-7759.

[15] T. C. Wilson, G. McSweeney, S. Preshlock, S. Verhoog, M. Tredwell, T. Cailly, V. Gouverneur, *Chem. Commun.* **2016**, *52*, 13277-13280.

[16] a) Y. Ye, S. D. Schimler, P. S. Hanley, M. S. Sanford, *J. Am. Chem. Soc.* **2013**, *135*, 16292-16295; b) T. Furuya, A. E. Strom, T. Ritter, *J. Am. Chem. Soc.* **2009**, *131*, 1662-1663.

[17] I. Kalvet, T. Sperger, T. Scattolin, G. Magnin, F. Schoenebeck, *Angew. Chem. Int. Ed.* **2017**, *56*, 7078-7082.

[18] R. Mockel, J. Hille, E. Winterling, S. Weidemuller, T. M. Faber, G. Hilt, *Angew. Chem. Int. Ed.* **2018**, *57*, 442-445.

[19] L. Candish, M. Teders, F. Glorius, *J. Am. Chem. Soc.* **2017**, *139*, 7440-7443.

[20] M. J. Harper, E. J. Emmett, J. F. Bower, C. A. Russell, *J. Am. Chem. Soc.* **2017**, *139*, 12386-12389.

[21] D. H. McDaniel, H. C. Brown, *J. Org. Chem.* **1958**, *23*, 420-427.

[22] a) F.-L. Lu, Y. M. A. Naguib, M. Kitadani, Y. L. Chow, *Can. J. Chem.* **1979**, *57*, 1967-1976; b) S. Shimizu, Y. Imamura, T. Ueki, *Org. Process Res. Dev.* **2014**, *18*, 354-358.

[23] M. J. Frisch, G. W. Trucks, H. B. Schlegel, G. E. Scuseria, M. A. Robb, J. R. Cheeseman, G. Scalmani, V. Barone, G. A. Petersson, H. Nakatsuji, X. Li, M. Caricato, A. V. Marenich, J. Bloino, B. G. Janesko, R. Gomperts, B. Mennucci, H. P. Hratchian, J. V. Ortiz, A. F. Izmaylov, J. L. Sonnenberg, Williams, F. Ding, F. Lipparini, F. Egidi, J. Goings, B. Peng, A. Petrone, T. Henderson, D. Ranasinghe, V. G. Zakrzewski, J. Gao, N. Rega, G. Zheng, W. Liang, M. Hada, M. Ehara, K. Toyota, R. Fukuda, J. Hasegawa, M. Ishida, T. Nakajima, Y. Honda, O. Kitao, H. Nakai, T. Vreven, K. Throssell, J. A. Montgomery Jr., J. E. Peralta, F. Ogliaro, M. J. Bearpark, J. J. Heyd, E. N. Brothers, K. N. Kudin, V. N. Staroverov, T. A. Keith, R. Kobayashi, J. Normand, K. Raghavachari, A. P. Rendell, J. C. Burant, S. S. Iyengar, J. Tomasi, M. Cossi, J. M. Millam, M. Klene, C. Adamo, R. Cammi, J. W. Ochterski, R. L. Martin, K. Morokuma, O. Farkas, J. B. Foresman, D. J. Fox, *Gaussian 16 Rev. B.01,* **2016**, Wallingford, CT.

[24] C. Y. Legault, *CYLview,* Version 1.0b, **2009**, Université de Sherbrooke, (http://www.cylview.org).
